# Design, synthesis and structure of novel G-2 melamine-based dendrimers incorporating 4-(*n*-octyloxy)aniline as a peripheral unit

**DOI:** 10.3762/bjoc.14.145

**Published:** 2018-07-09

**Authors:** Cristina Morar, Pedro Lameiras, Attila Bende, Gabriel Katona, Emese Gál, Mircea Darabantu

**Affiliations:** 1 Department of Chemistry, Babes-Bolyai University, 11 Arany János St., 400028 Cluj-Napoca, Romania; 2 University of Reims Champagne-Ardenne, ICMR, UMR 7312, BP 1039, 51687 Reims, France; 3 National Institute for Research and Development of Isotopic and Molecular Technologies, 67-103, Donath St., PO Box 700, 400293 Cluj-Napoca 5, Romania; 4 Department of Chemistry and Chemical Engineering, Hungarian Line of Study, Babes-Bolyai University, 11 Arany János St., 400028 Cluj-Napoca, Romania

**Keywords:** amination, dendrimers, melamines, nano-aggregates, 4-(*n*-octyloxy)aniline

## Abstract

**Background:** 4-(*n*-Octyloxy)aniline is a known component in the elaboration of organic materials with mesogenic properties such as *N*-substituted Schiff bases, perylene bisimide assemblies with a number of 2-amino-4,6-bis[4-(*n*-octyloxy)phenylamino]-*s*-triazines, amphiphilic azobenzene-containing linear-dendritic block copolymers and G-0 monomeric or dimeric dendritic liquid crystals with photochromic azobenzene mesogens. The present ab initio study explores a previously unknown use of 4-(*n*-octyloxy)aniline in the synthesis, structure and supramolecular behaviour of new dendritic melamines.

**Results:** Starting from 4-(*n*-octyloxy)aniline, seven G-2 melamine-based dendrimers were obtained in 29–79% overall yields. Their iterative convergent- and chemoselective synthesis consisted of S_N_2-Ar aminations of cyanuric chloride and final triple *N*-acylations and Williamson etherifications (→ G-2 covalent trimers) or stoichiometric carboxyl/amino 1:3 neutralisations (→ G-2 ionic trimers). These transformations connected G-1 chloro- and amino-termini dendrons to *m*-trivalent cores (triazin-2,4,6-triyl and benzene-1,3,5-triyl units) or tripodands (central building blocks), such as *N*-substituted melamines with 4-hydroxyphenyl or phenyl-4-oxyalkanoic motifs. Owing to the diversity of cores and central building blocks, the structural assortment of the dendritic series was disclosed by solvation effects (affecting reactivity), rotational stereodynamism and self-organisation phenomena (determining a vaulted and/or *propeller* macromolecular shape in solution). DFT calculations (in solution), (VT) NMR and IR (KBr) spectroscopy supported these assignments. TEM analysis revealed the ability of the title compounds towards self-assembling into homogeneously packed spherical nano-aggregates.

**Conclusions:** The (non)covalent synthesis and step-by-step structural elucidation of novel G-2 melamine dendrimers based on 4-(*n*-octyloxy)aniline are reported. Our study demonstrates the crucial influence of the nature (covalent vs ionic) of the dendritic construction in tandem with that of its central building blocks on the aptitude of dendrimers to self-organise in solution and to self-assembly in the solid state.

## Introduction

*N*-Substituted melamine (2,4,6-triamino-1,3,5-triazine)-based dendrimers are a class of macromolecules reported as early as 2000 by E. E. Simanek and co-workers [1] and then by K. Takagi and co-workers [2] as part of an innovative development of convergent [1,3–4] and divergent [1,5–6] strategies towards iterative dendritic synthesis. Along with their expansion, both the biological impact of the above arborescent structures, mainly as drug delivery systems [4,7–12], and their utilisation as organic materials have constantly been highlighted [13–17]. In the latter context, dendritic liquid crystals defines a well-established area in the organic materials domain [18], including few examples of *s*-triazine dendrimers exhibiting mesogenic behaviour. The first cases known so far refer to *N*-substituted G-0-3 dendritic melamines [19–21] with *n*-octyl peripheral groups as unconventional columnar liquid crystals and G-0 tris(triazolyl)triazines (available via the “click” reaction between 2,4,6-tris(ethynyl)-*s*-triazine and various icosanyloxyphenylazides) with liquid crystalline and luminescent properties [22].

On the other hand, mesogenic supramolecular perylene bisimide assemblies with a number of 2-amino-4,6-bis[(4-alkoxy)phenylamino]-*s*-triazines [23], amphiphilic azobenzene-containing linear-dendritic block copolymers [24] and G-0 monomeric or dimeric dendritic liquid crystals with photochromic azobenzene mesogens [25] called attention on the use of 4-(*n*-octyloxy)aniline as key building block in the above macromolecules’ elaboration. These recent findings can be seen as well as advances concerning the utilisation of 4-(*n*-octyloxy)aniline, a “traditional” source for mesogenic *N*-substituted Schiff bases [26–29].

Following up our contributions in the field of dendritic melamines’ synthesis, structural analysis [30–33] and electrochemistry [34–36], we recently become interested in the inclusion of 4-aminophenol, playing the role of peripheral unit, in G-0-2 dendritic melamines’ preparation, by applying the classic S_N_2-Ar amination of cyanuric chloride in iterative-convergent strategies. Depending on several factors such as (i) the variable π-deficiency of the *s*-triazine branch-cells, (ii) basicity and conformational nature of the diaza-six-membered saturated heterocycle as linker and (iii) the global molecular shape, the resulted 4-aminophenol-based melamines displayed relevant redox properties [36] and, in some cases, selective aptitudes to produce MOFs (metal-organic frameworks) [35].

All the above information prompted us towards an ab initio exploration of 4-(*n*-octyloxy)aniline (seen as the *n*-octyl ether derivative of 4-aminophenol) as starting material towards new melamine-based dendrimers as synthesis (feasibility and limits), structure and self-assembly propensity. To the best of our knowledge, no similar approach has been reported so far.

## Results and Discussion

### 1 Design

**Figure 1:**
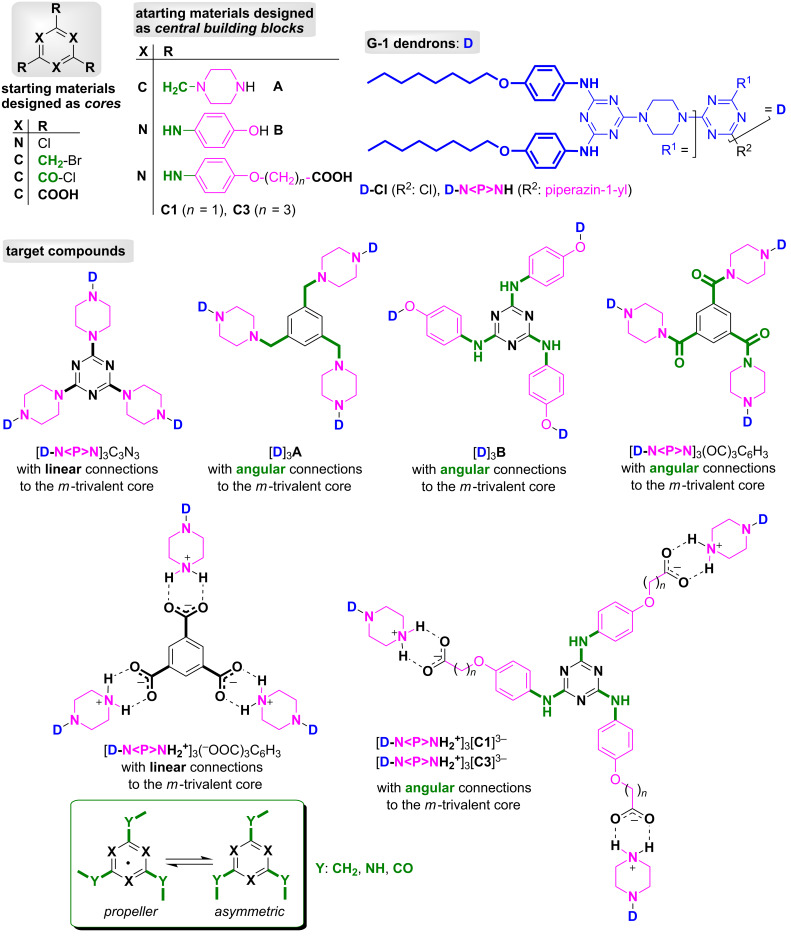
The key elements for design and construction of the targeted G-2 dendrimers.

The key elements for the construction and design of the targeted G-2 dendrimers are shown in Figure 1. Cyanuric chloride [1–17], 1,3,5-tris(bromomethyl)benzene [37] and trimesic acid trichloride [38] are commercial chemicals, classically known as reactive *m*-trivalent (hetero)aryl halo compounds, that are easily convertible into dendritic cores in reactions with various dendrons as *N*- and *O*-nucleophiles.

**Scheme 1:**
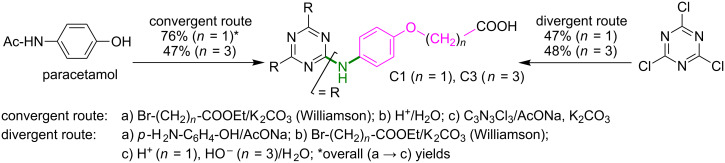
Convergent versus divergent three steps (a–c) synthesis of central building blocks **C1** and **C3**.

The preparation of the central building block **A** [1,3,5-tris(piperazinomethyl)benzene] consisted of the amination of 1,3,5-tris(bromomethyl)benzene with commercially available *N*-Boc-piperazine followed by deprotection, and was achieved according to the literature [39] (97% overall yield in our hands). Similarly, 2,4,6-tris[(4-hydroxy)phenylamino]-*s*-triazine **B**, a known starting material for plastics manufacturing [40] and divergent G-1 dendritic synthesis [41], was obtained using 4-aminophenol as amine nucleophile and reacting it with cyanuric chloride by applying a previously patented protocol [40] completed with our own subsequent improvements [36]. With regard to (4-aminophenoxy)alkanoic acid-based tripodal melamines **C1** and **C3**, we recently reported their synthesis in three steps via convergent (starting from *N*-acetyl-4-aminophenol, also known as paracetamol) and divergent (via **A**) strategies [42] which are summarised in Scheme 1.

All central building blocks (**A–C**) already contain a standard *m*-trivalent core (benzene-1,3,5-triyl or *s*-triazin-2,4,6-triyl) connected to 1,4-disubstituted six-membered (hetero)cycle linkers. These linkers were chosen based on their intimate nature: flexible (piperazin-1-yl)methyl (in **A**), rigid 1,4-phenylene (in **B**) and rigid 1,4-phenylene with adjustable alkoxy-spacers (*n* = 1, 3, in **C1** and **C3**, respectively).

G-1 chloro- and piperazine-dendrons, **D-Cl** and **D-N<P>NH**, as well as central building blocks **B**, **C1** and **C3**, had *s*-triazine rings linked by C(*s*-triazine)–N(exocyclic) partial double bonds, which exist due to classic LP(N, exocyclic) → π(C=N, *s*-triazine) delocalisation. A well-documented restricted rotation effect that causes diastereomerism is thus induced [43–45]. In the case of some dendritic melamines, this intrinsic feature can promote specific spatial arrangements at room temperature in solution, for example, *asymmetric* vs *propeller* (*C*_3_-symmetric) [30,36,45–46], “dendritic choreography” [47], “open-gates 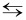 closed-gates” frontier rotamerism [31] and “in 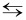 out” axial chirality [33].

In addition, in the targeted G-2 dendrimers (Figure 1), different connections of branches around the core (or central building blocks) were imagined, i.e., linear or angular (covalent vs ionic by carboxyl/amino neutralisation) and were seen as a complementary focus to our work towards structural diversity.

### 2 Synthesis: feasibilities and failures

#### 2.1 Synthesis of G-1 dendrons

**Scheme 2:**
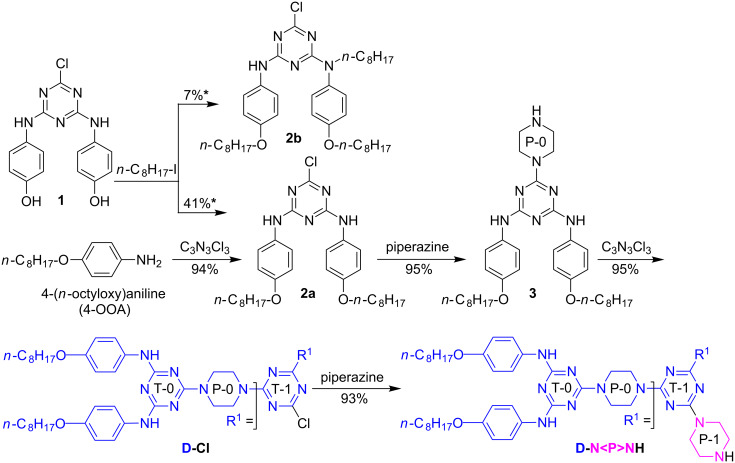
Synthesis of G-1 dendrons **D-Cl** and **D-N<P>NH**. *As partial conversions of **1** into **2a** and **2b** based on the effective amounts of these products isolated after column chromatography.

G-1 dendrons **D-Cl** and **D-N<P>NH** (Figure 1) were prepared according to the chemistry depicted in Scheme 2. Reaction conditions and quantitative results are listed in Table 1.

**Table 1:** Reagents and conditions in the synthesis of compounds **2a**, **2b**, **3**, **D-Cl** and **D-N<P>NH**.

Entry	Reaction	Conditions^a^

1	**1** → **2a**, **2b**	(**i**) 1.00 equiv *n*-C_8_H_17_-I, 8.00 equiv K_2_CO_3_, acetone, −13 °C, 3 h; rt, 12 h; (**ii**) 1.00 equiv *n*-C_8_H_17_-I, rt, 72 h; 50 °C, 8 h/N_2_
2	4-OOA → **2a**^b^	(**i**) 0.50 equiv C_3_N_3_Cl_3_, acetone, 0–5 °C, 2 h; (**ii**) 1.00 equiv NaHCO_3_, H_2_O, 0–5 °C; 45 °C, 3 h; rt, 18 h/N_2_
3	**2a** → **3**	4.00 equiv piperazine, 1.00 equiv K_2_CO_3_, THF, rt, 34 h
4	**3** → **D-Cl**	0.50 equiv C_3_N_3_Cl_3_, 1.00 equiv K_2_CO_3_, THF, −10 °C, 3 h; rt, 36 h; reflux, 24 h
5	**D-Cl** → **D-N<P>NH**	4.00 equiv piperazine, 1.00 equiv K_2_CO_3_, THF, rt, 40 h; reflux, 15 h

^a^For comments about these conditions, see section 3.1. ^b^The same double amination of cyanuric chloride with 4-(*n*-octyloxy)aniline was reported by S. Yagai and co-workers in 2012 [23], with no isolation of **2a**, as it was submitted in situ to ammonolysis to give 2-amino-4,6-bis[4-(*n*-octyloxy)phenylamino]-*s*-triazine (75% overall yield).

We initially considered using the previously reported 4-hydroxyphenyl *N*-substituted 2-chloro-4,6-diamino-*s*-triazine **1** [36,48–49] as starting material. To our disappointment, the attempted *O*,*O*’-bis-alkylation of **1** with 1-iodooctane (Williamson etherification, Table 1, entry 1), afforded a multicomponent reaction mixture (TLC monitoring, 100% conversion of **1**), from which we succeeded in isolating only G-0 chloro-dendron **2a** and the *O*,*O*’,*N*-tris-alkylated side product **2b** by column chromatography. Other efforts (not detailed in the present report), such as manipulation under dark conditions or replacing the iodine in the alkylating reagent by bromine, provided even more unsatisfactory results, e.g., the *O*-mono-alkylated product. By contrast, the double amination of cyanuric chloride with commercial 4-(*n*-octyloxy)aniline (Table 1, entry 2) gave the desired G-0 chloro-dendron **2a** with excellent yield. We note the choice of several authors towards the synthesis of 4-(*n*-octyloxy)aniline by Williamson etherification of 4-nitrophenol or of *N*-acetyl-4-aminophenol (paracetamol) and subsequent reduction of the nitro group [23,25,27,29] or acidolysis of the amide sequence [24,26,28]. Our expertise on the use of paracetamol under Williamson etherification conditions [42] (Scheme 1, convergent route) did not impel us in the direction of preliminary 4-(*n*-octyloxy)aniline preparation; we also took into account its convenient price.

Using G-0 dendron **2a**, we synthesised G-1 dendrons in two (→ **D-Cl**) or three (→ **D-N<P>NH**) orthogonal transformations, with excellent overall yields (90% and 84% from **2a**, respectively). The complete chemoselectivity observed during the installation of the piperazine linkers on **2a** (Table 1, entry 3) and on **D-Cl** (Table 1, entry 5) was ensured by the use of a 300% molar excess of this inexpensive reagent. We note the long-reaction time and large temperature domains (Table 1) required in order to obtain the quantitative results shown in Scheme 2 (to be discussed in section 3.1). Conversely, all G-0 and G-1 dendrons were easily purified by simple crystallisations from boiling methanol or ethanol (Supporting Information File 1, pp. S3–S6; pp. S12–S23, Figures S1–S24).

#### 2.2 Synthesis of G-2 dendrimers

**Scheme 3:**
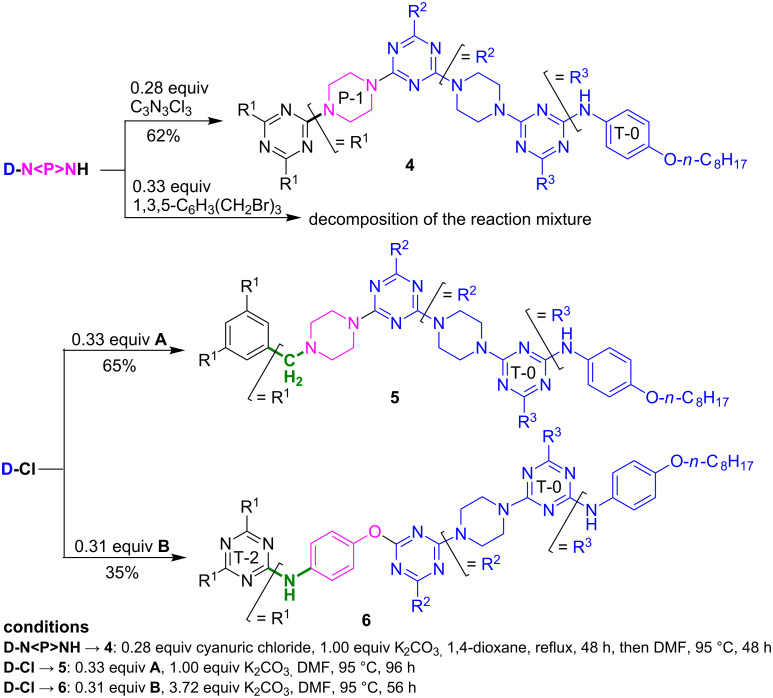
Synthesis of G-2 dendrimers **4**–**6** by *m*-trimerisations of G-1 dendrons **D-Cl** and **D-N<P>NH**.

The synthesis of G-2 dendrimers **4–6** (Scheme 3) revealed a moderate reactivity of G-1 dendrons **D-N<P>NH** and **D-Cl** with cyanuric chloride and central building blocks **A** and **B**. Therefore, in addition to TLC, all reactions were monitored by HRMS as well. They were stopped when no further reaction was observed by TLC and when the expected molecular peak was identified by HRMS (ESI^+^, ACN + TFA).

Under demanding thermal conditions, the complete amination of cyanuric chloride by **D-N<P>NH** gave the G-2 dendrimer **4** with satisfactory yield. By contrast, treatment of 1,3,5-tris(bromomethyl)benzene with **D-N<P>NH** (3 equiv, 72 h in refluxing 1,4-dioxane in the presence of K_2_CO_3_ as proton scavenger) resulted in no dendrimer formation; decomposition of **D-N<P>NH** was observed instead (as determined by additional NMR monitoring).

Nevertheless, the desired G-2 dendrimer **5** could be obtained through an alternative route, namely, by amination of G-1 chloro-dendron **D-Cl** with building block **A** (Figure 1) under harsh conditions.

To obtain dendrimer **6**, we expected to exploit the nucleophilicity of the phenoxide groups in the *N*,*N*’,*N*”-tris(4-hydroxyphenyl)melamine **B** (Figure 1) based on the previous example of P. Gamez and co-workers [41], which involved reaction with 2-chloro[4,6-di(pyridin-2-ylamino)]-*s*-triazine in the presence of DIPEA (69% yield after 48 h at 85 °C in pyridine). However, when dendrimer **6** was obtained by reacting **B** (0.31 equiv) with **D-Cl** (1.00 equiv), the reaction only proceeded properly when 4.00 equiv K_2_CO_3_ per phenolic OH group in **B** was used as the proton scavenger and deprotonating reagent, i.e., in an identical acid/base molar ratio as that used earlier in the triple Williamson etherification of **B** (Figure 1, Scheme 1, divergent route) with bromoalkanoic acid ethyl esters [42]. Even so, the yield of **6** was modest, 35%.

**Scheme 4:**
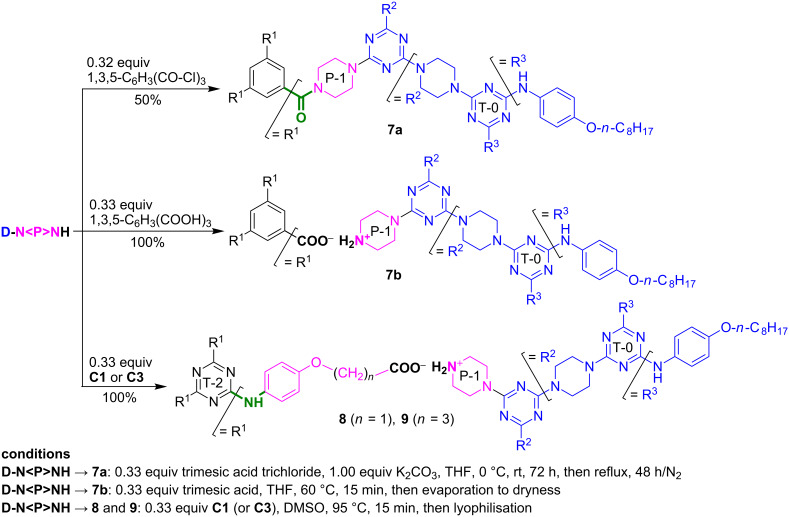
Synthesis of G-2 dendrimers **7**–**9** by *m*-trimerisations of G-1 dendron **D-N<P>NH**.

Furthermore, we obtained G-2 dendrimers **7a** and **7b** (Scheme 4) by the triple amidation of trimesic acid trichloride upon treatment with G-1 dendron **D-N<P>NH** (covalent *m*-trimerisation →**7a**) or by simple neutralisation of the latter with trimesic acid (ionic *m*-trimerisation →**7b**), the product of which was symbolised as [**D-N<P>NH_2_^+^**]_3_(^−^OOC)_3_C_6_H_3_ (Figure 1).

In the synthesis of **7a**, unexpectedly difficult conditions for this type of reaction were, once again, required.

Similar amidations run in triplicate using instead the acid chlorides of **C1** and **C3** provided negative results. Thus, although the protocol for the conversion of these (4-aminophenoxy)alkanoic acid-based tripodal melamines into the corresponding tri acid chlorides (not depicted in Scheme 4) followed typical procedures (in refluxing thionyl chloride with SO_2_ and HCl generation), a long reaction time (up to 24 h, as determined by HRMS monitoring) was mandatory for this transformation to go to completion. The crude isolated products exhibited very low solubility in solvents usually recommended for standard *N*-acylations (acetone, THF, 1,4-dioxane, pyridine, etc.). Treatment of these acid chlorides with G-1 dendron **D-N<P>NH** (3 equiv) under various conditions (microwave-assisted organic reactions included [50]) resulted in no reaction and the starting materials, **D-N<P>NH** and **C1** (or **C3**) being recovered in good yields after obligatory aqueous work-up. To our surprise, even the trivial derivatisation of the acid chlorides as ethyl esters (precursors of **C1** and **C3**, Scheme 1, divergent route) required at least 24 h in absolute EtOH and only proceeded upon heating. Therefore, we had to limit our efforts to the stoichiometric salts (Figure 1), symbolised as [**D-N<P>NH_2_^+^**]_3_[**C1**]^3−^ (**8**) and [**D-N<P>NH_2_^+^**]_3_[**C3**]^3−^ (**9**). These dendritic constructions by ionic *m*-trimerisations occurred smoothly and in quantitative yields (Scheme 4) (Supporting Information File 1, pp. S6–S11; pp. S24–S39, Figures S25–S54).

### 3 Structural assignments

Our research continued with a structural study, implemented by means of DFT calculations, solution (VT) NMR techniques, IR (KBr) spectroscopy and TEM analysis.

#### 3.1 Assignments based on DFT calculations of G-0 and G-1 dendrons: optimal geometry and solvation effects

**Figure 2:**
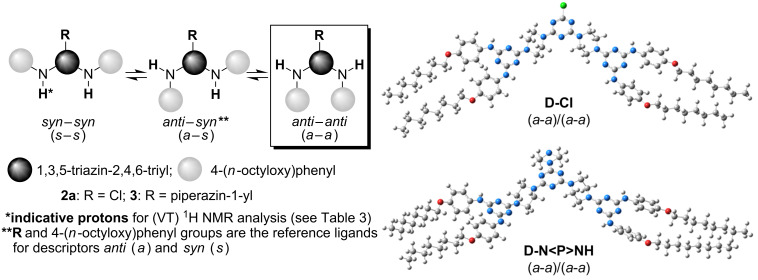
The three terms rotamerism of G-0 dendrons **2a** and **3** about the C(*s*-triazine)–N(exocyclic) partial double bonds.

First, we attempted to explain the generally difficult conditions encountered during synthesis (Schemes 2–4) based on DFT calculations [50–56] (Figure 2 and Table 2).

**Table 2:** Full geometry optimisation of G-0 dendrons **2a**, **3** [51–54], solvation energies of G-0 and G-1 dendrons **2a**, **3**, **D-Cl**, **D-N<P>NH** [54] and lone pair (LP) electron occupation of piperazine N^sp3^ nitrogens in dendrons **3** and **D-N<P>NH** [56–57].

No.	Relative conformational total electronic Energy Δ*E* (kJ mol^−1^)^a^ in the gas phase in DMSO			Δ*G* of solvation (kJ mol^−1^) for the (*a–a*) major rotamer^a^		LP electron occupation of piperazine N^sp3^ nitrogens (*e*)^b^	

	(*s–s*)	(*a–s*)	(*a–a*)	THF^c^	1,4-dioxane^d^	THF	1,4-dioxane

**2a**	10.464 17.371	8.329 15.558	0.000 0.000	−146.37	−104.39	–	–
**3**	4.395 5.923	4.730 4.504	0.000 0.000	−167.54	−122.51	1.92	1.92
**D-Cl**	–	–	–	−378.66	−284.28	–	–
**D-N<P>NH**	–	–	–	−401.81	−301.36	1.93	1.92

^a^See Experimental and Supporting Information File 1 (pp. S40 ff) for details of this DFT calculation. ^b^Elementary electric charge carried by a single electron. ^c^Dielectric constant, ε = 7.4257. ^d^Dielectric constant, ε = 2.2099.

As in the case of other amino-*s*-triazines [30–33,36,45–46], for G-0 dendrons **2a** and **3**, a topologically idealised model predicted a three terms (*syn–syn* 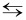 *anti–syn* 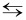 *anti–anti*) rotational equilibrium about the C(*s*-triazine)–N(exocyclic) (Figure 2) partial double bonds, at room temperature. Similar to simpler 4-hydroxyphenyl-based amino-*s*-triazine analogues of **2a** and **3** [35–36], the optimised (*a–a*) geometry was found to be largely dominant, both in the gas phase as well as in solution, e.g., DMSO, which was the most appropriate solvent for (VT) ^1^H NMR and TEM investigations (see section 3.2).

As expected, the relative Δ*E* values for the rotational diastereomerism decreased (i) with the decrease of the steric hindrance between the three (hetero)aryl rings in rotamers (*s–s*) > (*a–s*) > (*a–a*), and (ii) with the weakening of the π-deficiency of the *s*-triazine, **2a** > **3**. The same optimal geometry (*a–a*) was found in duplicate, (*a–a*)/(*a–a*), in G-1 dimers **D-Cl** and **D-N<P>NH** (Figure 2).

Furthermore, for the major (*a–a*) rotamers, calculations also revealed high solvation energies in the case of all four G-0 and G-1 dendrons. For a single compound, the solvation energy was 33–40% higher in THF vs 1,4-dioxane. Unsurprisingly, piperazine dendrons were more solvated than their chloro precursors, e.g., **3** vs **2a** (+14% in THF, +17% in 1,4-dioxane), and **D-N<P>NH** vs **D-Cl** (about +6% in both solvents). Taking into account the negligible decrease in LP electron occupation of the piperazine N^sp3^ nitrogens in **3** and **D-N<P>NH** as well, we ascribed the low reactivity of G-1 dendrons **D-Cl** and **D-N<P>NH** in our S_N_2-Ar amination conditions to the increased solvation of their (*a–a*)/(*a–a*) rotamers (+140–170%, i.e., more than double) compared to those of the (*a–a*) rotamers of G-0 **3** and **2a**.

In addition to all the above, due to the steric accommodation of 12 *n*-octyloxy chains, the occurrence of a starburst effect [18,58–59] during the covalent *m*-trimerisations (Scheme 3), was also presumed (see section 3.2.1).

#### 3.2 Assignments by means of NMR and IR spectroscopy

The ^1^H NMR data deserving of comment are listed in Table 3.

**Table 3:** Relevant (VT) ^1^H NMR, temperature gradients and 2D-^1^H-DOSY NMR data (500 MHz, DMSO-*d*_6_) of G-0 dendrons **2a**, **3**, G-1 dendrons **D-Cl**, **D-N<P>NH** and G-2 dendrimers **4–6**, **7a**, **7b**, **8**, **9**.

No.	δ_H_ (ppm) values of indicative NH protons (see Figure 2)				temperature gradients (TGs) as (Δδ_H_/Δ*Τ*) × 10^3^ (ppb K^−1^)^a^		*D*^b^ (µm^2^ s^−1^)	*d*_H_^b^ (nm)

	NH adjacent to *s*-triazine T-0		NH adjacent *s*-triazine T-2		NH adjacent to *s*-triazine T-0	NH adjacent *s*-triazine T-2		

	298 K	363 K	298 K	363 K				

**2a**^c^	10.03 9.96 9.85	9.63	–	–	−6.15 −5.08 −3.38		198	1.10
**3**	8.87	8.50	–	–	−5.69		191	1.14
**D-Cl**	9.08	8.70	–	–	−5.85		129	1.69
**D-N<P>NH**	8.93	8.57	–	–	−5.54		110	1.98
**4**	9.71	8.93	–	–	−12.00		89	2.45
**5**	8.92	8.51 8.53 8.55 8.60	–	–	−6.31 −6.00 −5.69 −4.92		88	2.48
**6**^d^	–	8.55	–	9.02	–		–	–
**7a**	8.94	8.55	–	–	−6.00		107	2.04
**7b**	8.93	8.53	–	–	−6.15		105 225	2.08 0.97
**8**	8.93	8.54	8.93	8.60	−6.00	−5.08^e^	113	1.93
**9**	8.94	8.54	8.70	8.57	−6.15	−2.00^f^	115	1.90

^a^TGs calculated as [(*δ*_H_^298 K^ − *δ*_H_*^T ^*^K^)/(298 K – *T* K)] × 10^3^ < 0 (where *T* = 363 K). ^b^*d*_H_ (hydrodynamic diameter) issued from *D* [diffusion coefficient observed in 2D-^1^H-DOSY NMR charts as 5.0 mM (**2a**, **3**, **D-Cl**, **D-N<P>NH**, **5**, **8** and **9**) or 2.5 mM (**4**, **7a** and **7b**) in DMSO-*d*_6_ (*η*, dynamic viscosity 2.00 × 10^−3^ kg m^−1^ s^−1^) at 298 K] by applying the Stokes–Einstein equation. ^c^Multiple *δ*_H_ and TG values due to more than one (*anti–anti* major 64% vs *anti–syn* minor 36%) species found in a frozen rotational equilibrium in agreement with the highest π-deficiency of the *s*-triazine ring in the analysed series (Figure 2; Supporting Information File 1, p. S12, Figure S1). ^d^Due to the low solubility of compound **6** in DMSO-*d*_6_, its convincing NMR spectra could be obtained at 363 K only. ^e^TG as −6.92 ppb K^−1^ in the starting material **C1**. ^f^TG as −6.07 ppb K^−1^ in the starting material **C3**.

**Figure 3:**
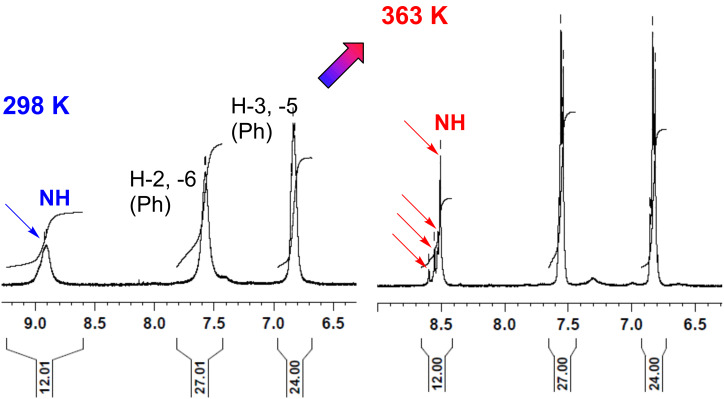
Comparative details from ^1^H NMR spectra of G-2 dendrimer **5** (500 MHz, 5.0 mM in DMSO-*d*_6_).

**3.2.1 Rotational diastereomerism at dendritic level:** As expected, due to it having the highest π-deficient *s*-triazine group, at room temperature only G-0 chloro dendron **2a** exhibited the nearly frozen nature of the *anti* 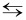 *syn* (Figure 2; Supporting Information File 1, pp. S12, Figure S1) rotational equilibrium on a 500 MHz ^1^H NMR timescale. In contrast, under the same conditions, the rotational diastereomerism of all other compounds (G-0, G-1 and G-2) was identified, as the classically entitled “slow exchange status between unequally populated sites” [60–61]. At room temperature, dendrimers **5**, **7a**, **8** and **9** displayed unique signals for the topologically equivalent ^1^H and ^13^C nuclei of the *m-*trivalent core and their adjacent angular connections, i.e., consistent with a *propeller* as local dominant arrangement (Figure 1) [45–46]. Except for G-2 dendrimer **5**, upon heating to 90 °C, all compounds became single mediated structures, in states with rapid and free rotation about all the C(*s*-triazine)–N(exocyclic) bonds. In contrast, in the ^1^H NMR spectrum of dendrimer **5** recorded at 90 °C, multiple resonances (Table 3 and Figure 3) were displayed by the indicative NH protons, suggesting the occurrence of a significant peripheral steric hindrance, starburst effect [18,58–59], against authentic free rotation upon heating. This fact was not quite surprising because we previously reported a related situation in the case of some G-0 dendrons as *N*,*N*’-disubstituted 2-chloro-4,6-diamino-*s*-triazines with bulky azaspirodecane and propane-1,3-diol ligands and entitled this “abnormal” behaviour “pseudo freely rotating*”* status [31].

**3.2.2 Dendritic solvation:** In line with solvation energy calculations (Table 2), the temperature gradients (TGs) of the indicative NH protons provided additional information on the solvation in the regions surrounding the *s*-triazine units (branch-cells, T-0 or cores T-2, Scheme 3 and Scheme 4). According to H. Kessler (in 1982, [62]), this parameter is appropriate for describing amide protons solvation in p→π conjugated systems, such as –N(H)–C(=O)– ↔ –N**^+^**(H)=C(–O**^−^**)– in peptides and proteins, in water. Later on (in 2008), X. K. Moreno and E. E. Simanek [47] demonstrated the validity of the above TGs in the case of amino-*s*-triazines, –N(H)–C(=N–)– ↔ –N**^+^**(H)=C(–N**^−^**–)–, as well, in DMSO-*d*_6_. Following this new insight, if the TG values of “amidine-like” protons in amino-*s*-triazines are more negative than −4 ppb K^−1^ in strong hydrogen bond acceptor solvents, such as DMSO-*d*_6_ [45], then the NH groups are exposed to the solvent rather than developing intramolecular hydrogen bonds. Conversely, a TG value less negative than −4 ppb K^−1^ discloses the NH group preference towards intramolecular hydrogen bonds formation, at room temperature, e.g., with *s*-triazine LPs N-1, -3, -5. If so, the compounds under investigation (Table 3) exhibited TG values denoting significant (peripheral) >N–H···O=SMe_2_ hydrogen bond interactions. TGs moderately increased, from −5.55 ppb K^−1^ in G-1 **D-N<P>NH** to about −6.00 ppb K^−1^ in G-2 dendrimers, together with the hydrodynamic diameters, *d*_H_, except dendrimers **4** and **5**. Dendrimer **4** was by far the most NH/DMSO solvated of the series (TG around −12.00 ppb K^−1^) in conjunction with one of the highest *d*_H_ values, of 2.45 nm.

On the other hand, an evaluation of the TG values of the inner (T-2) vs the peripheral (T-0) NH groups in ionic dendrimer **8** showed a comparable aptitude for NH/DMSO H-binding, −5.08 ppb K^−1^ (−6.92 ppb K^−1^ in the starting material **C1**) vs −6.00 ppb K^−1^, respectively. Furthermore, the replacement of the central methylene spacer (*n* = 1, in **8**) with a trimethylene one (*n* = 3, in **9**) had no influence on the peripheral (T-0) NH groups/DMSO solvation as they maintained almost the same TG (−6.00 ppb K^−1^ in **8** vs −6.15 ppb K^−1^ in **9**). In contrast, the inner (T-2) NH groups of **9** appeared “protected” against DMSO (as a hydrogen bond acceptor) because their TG was only −2.00 ppb K^−1^, (much less negative than in the starting material **C3**, −6.00 ppb K^−1^), i.e., the three identical intramolecular >N–H···LP(N-1, -3, -5 of *s*-triazine T-2 core) interactions prevailed, being, most likely, a consequence of the aforementioned (Figure 1) *C*_3_ symmetric *propeller* as local arrangement.

**3.2.3 Assignment of ionic interactions in dendrimers 7b, 8 and 9:** On the ^1^H NMR timescale in DMSO-*d*_6_, the triple ionic interactions in dendrimers **7b**, **8** and **9** (Scheme 4) could only be indirectly assigned_,_ due to rapid proton interchange, symbolised as:

3 **D-N<P>NH** + (**H**OOC)_3_R 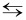 [**D-N<P>NH_2_^+^** ^−^OOC]_3_R

As a consequence of the triple ionisation of the carboxyl groups in tripodal melamines **C1** and **C3**, only a remote upfield shift of their ^1^H δ_NH_ values was observed: from 9.47 ppm (in **C1**, [42]) to 8.93 ppm (in the tris-anion of **8**) and from 8.95 ppm (in **C3**, [42]) to 8.70 ppm (in the tris-anion of **9**) (Table 3). A different diagnosis, based on aliphatic carboxyl group ionisation thus promoting ^1^H shielding of proximal (α and β) methylene protons, was reported in the case of some G-2 PAMAM ionic dendrimers obtained by –COOH/H_2_N– neutralisation (NMR solvent, CDCl_3_) [63].

However, in the case of the cationic counterpart, if a more illustrative series was examined, i.e., **D-N<P>NH**, **4**, **7a**, **7b**, **8** and **9** (Table 4), δ_H_ resonances of the methylene protons α-to the N^sp3^ nitrogen of P-1 piperazine linker (in **D-N<P>NH** vs **7b**, **8** and **9**) revealed minor fluctuations, between 2.97 and 3.15 ppm. These resonances were found much further upfield with respect to the shifts of the methylene protons α-situated vs the piperazine P-1 N^sp2^ nitrogen involved in p→π LP(N)→π(C=N, *s*-triazine; C=O, amide) conjugation (3.78–3.91 ppm).

**Table 4:** ^1^H NMR discrimination of methylene protons in piperazine P-1 linkers of compounds **D-N<P>NH**, **4**, **7a**, **7b**, **8** and **9**.

Methylene locations	Compound δ_H_ (ppm) (500 MHz, DMSO-*d*_6_, 298 K)					

	**D-N<P>NH**	**4**	**7a**	**7b^a^**	**8^a^**	**9^a^**

α-to N^sp3^	3.13	–	–	3.09	2.97	3.15
α-to N^sp2^	3.91	3.80	3.78	3.78	3.78	3.78

^a^Downfield shifted resonances (Δδ as +0.25 and +0.33 ppm) of α-CH_2_ protons to –NH_3_^+^ group were reported in the case of some G-2 PAMAM dendrimers obtained by –COOH/H_2_N– neutralisation (CDCl_3_) [63].

To conclude, except the well-documented deshielding promoted by the magnetic anisotropy of π-delocalised systems, such as *s*-triazines and amides [64], no such vicinal effect created by a quaternary EWG of type >N^sp3^H_2_^+^ [63] was detected in the piperazine P-1 linker of compounds **7b**, **8** and **9**, even in the case of the strongest proton donor, the trimesic acid (p*K*_a_ = 3.12, 3.89 and 4.70).

In addition, only in G-1 amino-dendron **D-N<P>NH** the ^1^H NMR integration of its termini piperazine P-1 linker disclosed the presence of just 4 methylene protons (instead of 8; Supporting Information File 1, pp. S21, S22, Figures S20 and S21) at room temperature as well as at 90 °C, presumably due to their longer *T*_1_ value (longitudinal relaxation time) [60]. No such spectral appearance was observed in the case of G-0 precursor **3** (Supporting Information File 1, p. S17, Figures S11 and S12). Subsequent to the use of **D-N<P>NH** in covalent (→ **4**, **7a**, **5**) or ionic (→ **7b**, **8** and **9**) *m*-trimerisations, the same ^1^H NMR integration evidenced, throughout, the existence of the expected 8 H/P-1 unit.

**Figure 4:**
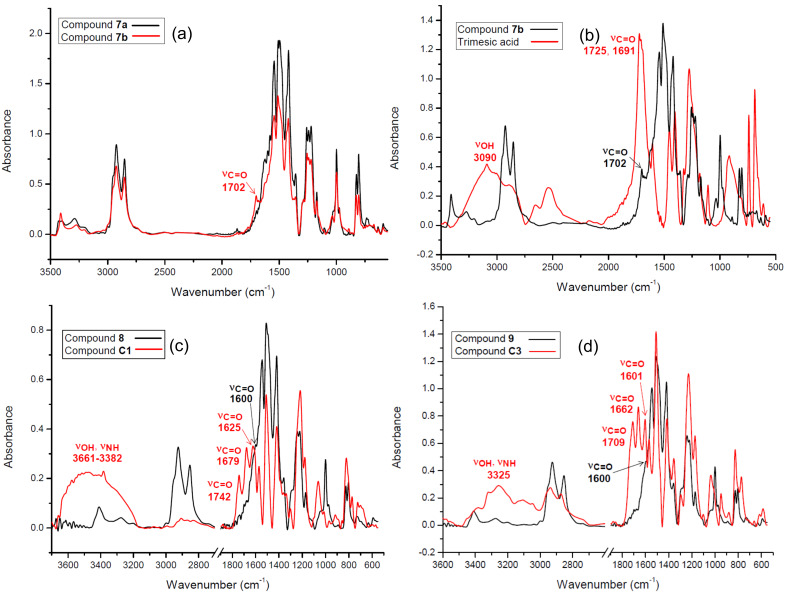
Comparative IR spectra (KBr) of compounds **7a** vs **7b** (a), **7b** vs trimesic acid (b), **8** vs **C1** (c) and **9** vs **C3** (d).

On the IR timescale (Figure 4a–d), the comparative spectra of compounds **7a**/**7b**, **7b**/trimesic acid, **8**/**C1** and **9/C3** fully proved the presence of only the tris-carboxylate anions [63,65–66].

(i) The spectrum of dendritic salt **7b** was almost the duplicate of that of covalent **7a** (Figure 4a), except for the presence of a weak band located at 1702 cm^−1^ which was attributed to the ν_C=O_ stretching absorption of the tris-carboxylate anion in **7b** [65–66].

(ii) Indeed (Figure 4b), the shift to a lower field and weaker intensity of the strong ν_C=O_ band (from 1725 cm^−1^ for the COOH groups of trimesic acid to 1702 cm^−1^ for the COO^−^ groups of **7b**), together with the disappearance of the ν_OH_ stretching band (3090 cm^−1^) of the carboxyl groups (strongly H-associated in trimesic acid) confirmed the existence of the trimesic tris-carboxylate anion in **7b**. Nevertheless, the stretching (ν_NH_, 3000–2700 cm^−1^) or deformation (δ_NH_, 1620–1560 cm^−1^) bands [66–67] of the protonated >NH_2_^+^ group of the P-1 piperazine linker could not be definitely allocated due to the overlapping in the above regions between the absorptions of the piperazine methylene groups (ν_CH2_), carbonyl bonds (ν_C=O_) in the COO^−^ groups, aryl bonds (ν_C=C_) and *s*-triazine bonds (ν_C=N_).

(iii) Similar attributions were applied in the case of ionic dendrimers **8** (Figure 4c) and **9** (Figure 4d). The vanishing of the carboxyl ν_OH_ bands from **C2** and **C3** in the dendritic environment of **8** and **9** established the ionic nature of the latter. Due to their overall lower symmetry in comparison with trimesic acid, tripodal aminophenoxy-acids-based *N*-substituted melamines **C2** and **C3** exhibited multiple (3–4) carboxyl ν_C=O_ stretching absorptions, in agreement with their different degrees of H-bond association, i.e., dimers (1742 and 1709 cm^−1^), polymers (1679, 1625, 1622 and, presumably, even 1600 cm^−1^). Succeeding the ionic trimerisations, all these bands were absent in the IR spectra of **8** and **9**, with the remaining carboxylate ν_C=O_ stretching band being located most likely at approximately 1600 cm^−1^.

In conclusion, the exact protonated site in ionic dendrimers **7b**, **8** and **9** could not be attributed accurately either in solution (^1^H NMR), or in the solid state (IR). Consequently, their proposed cationic structures shown in Figure 1 and Scheme 4 (linker P-1 as piperazin-1-ium moiety) should be seen intuitively, a proton sponge-like comportment of G-1 piperazine dendron **D-N<P>NH** being not entirely ruled out [68]. In line with this hypothesis, we recently described [35] the aptitude of the non-*O*-*n*-octylated analogue of G-2 dendrimer **4** (Scheme 3) to deprotonate Hemin completely (1:5 molar ratio), thus generating a new MOF for H_2_O_2_ amperometric detection. More insights on this debate were obtained by means of DFT calculations only (see section 3.3).

#### 3.3 Assignments by means of 2D-^1^H-DOSY NMR in tandem with DFT calculations at dendritic level

Except for dendrimers **4** (*d*_H_ 2.45 nm) and **5** (*d*_H_ 2.48 nm) (Table 3), in the series of trimers **7*–*9** the expected increase in the hydrodynamic diameters (*d*_H_) with respect to their G-1 monomeric precursor, **D-N<P>NH** (1.98 nm), was negligible (1.90*–*2.04 nm).

**Figure 5:**
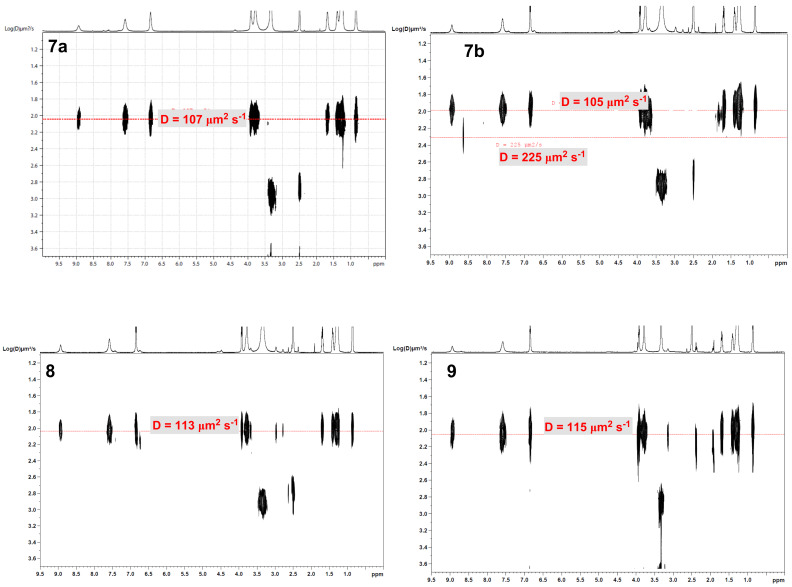
2D-^1^H-DOSY NMR charts (DMSO-*d*_6_, 500 MHz, 298 K) of compounds **7a**, **7b** (2.5 mM), **8** and **9** (5.0 mM).

2D-^1^H-DOSY NMR charts of G-2 dendrimers **7a** (covalent) vs ionic **7b**, **8** and **9** (Figure 5) displayed unique structures, with a partial dissociation of **7b**, presumably due to the sterically induced imperfect accommodation of the three G-1 cationic dendrons **D-N<P>NH_2_^+^** around the smallest central tris-anion of the series, trimesic tris-carboxylate.

In other words, the 2D-^1^H-DOSY NMR charts were consistent with the envisaged 3(**D-N<P>NH**):1 (trimesic acid, **C1** or **C3**) stoichiometric assembly of dendrimers **7b**, **8** and **9**. That is, we had to assume that in series **D-N<P>NH**, **7–9**, the correlation between *D* values (by means of hydrodynamic diameters *d*_H_, Table 3) and the macromolecular size was not direct, i.e., dendritic structures with different molecular weight displaced related volumes of solvent. The above encountered situation was comparable to that of some PAMAM dendrimers exhibiting *D* values around 100 μm^2^ s^−1^ [69].

**Figure 6:**
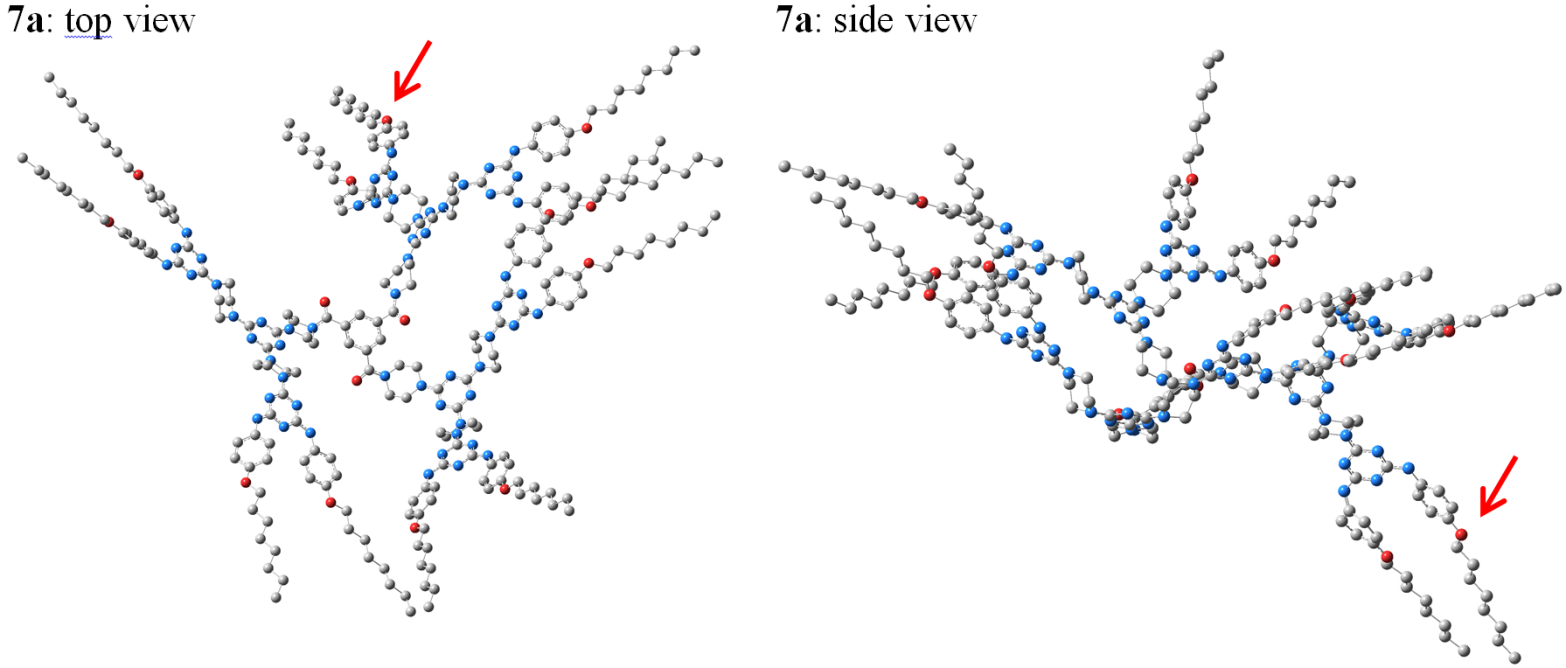
The DFT optimised geometry at M062X/def2-TZVP level of theory of G-2 dendrimer **7a** in DMSO (hydrogen atoms were omitted for reasons of simplicity).

**Figure 7:**
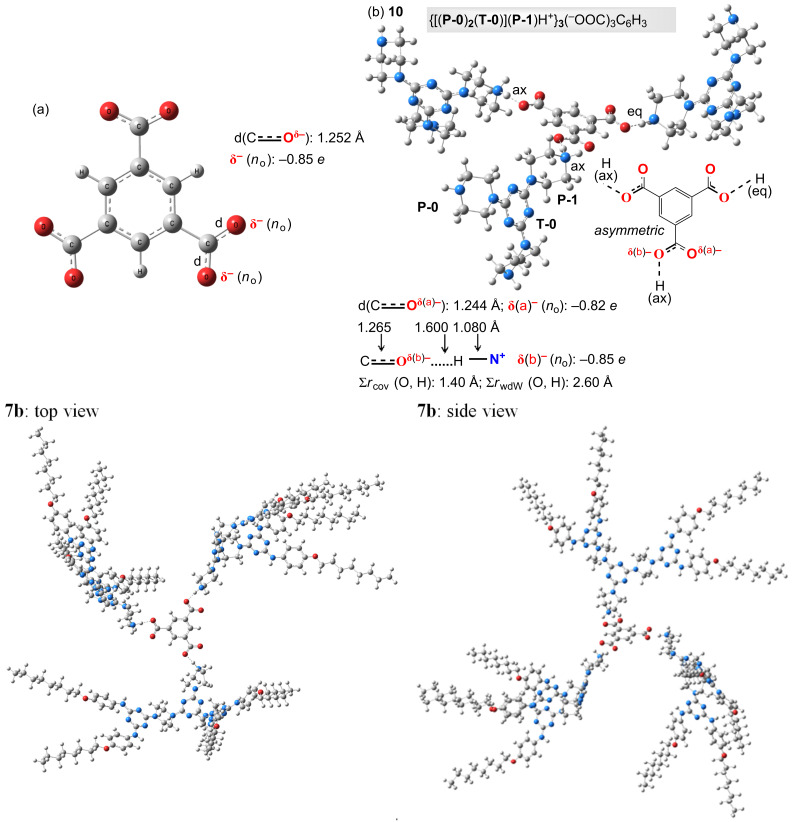
The DFT optimised geometry at M062X/def2-TZVP level of theory of trimesic tris-carboxylate anion (a), of model G-1 dendrimer **10** (b) and of G-2 dendrimer **7b** in DMSO.

**Figure 8:**
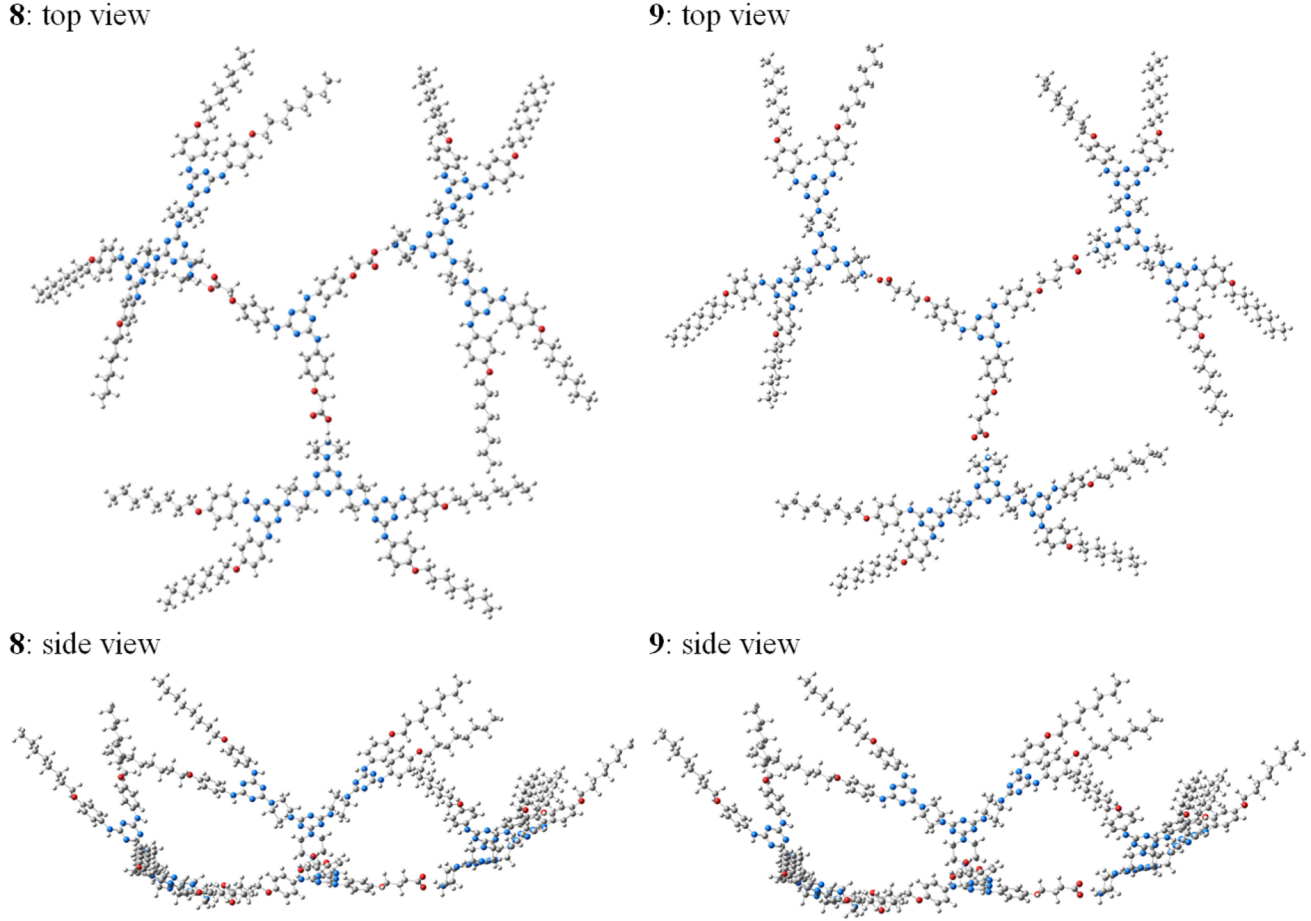
The DFT optimised geometry at M062X/def2-TZVP level of theory of G-2 dendrimers **8** and **9** in DMSO.

Therefore, an additional effort to estimate the above situation in the case of dendrimers **7–9**, by means of DFT calculations in solution (DMSO) (Figures 6–8), was straightforward.

First, the optimisation of the geometry of G-2 dendrimer **7a** (Figure 6) identified its global form to be almost vaulted although the degree of freedom of the six G-1 dendritic units appeared restricted. Indeed, one of them (marked by the red arrow) had an opposite direction to the other five, caused, probably, by the *propeller* orientation (Figure 1) of the three covalent junctions around the benzene core.

In contrast, the energetic minimum of the ionic analogue **7b** could be accessed in a gradual approach only. Thus, we had to start from a preliminary DFT calculation of the trimesic tris-carboxylate anion (Figure 7a) because its identical partial double bond lengths (dC
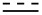
O^δ−^) and oxygen atoms negative charges values (δ^−^, as Natural Population (*n*_o_) according to the NBO analysis) were then taken as references with regard to those of the same tris-anion installed in a model G-1 ionic environment, **10** (Figure 7b). We considered dendrimer **10** as a simplified version of **7b** from which the 4,6-bis[4-(*n*-octyloxy)phenylamino]-*s*-triazin-2-yl G-0 branches were removed. Oxygen atoms negative charges in **10** (Figure 7b), δ(a)^–^ and δ(b)^–^, did not differ significantly vs those of the initial trimesic tris-carboxylate anion (δ^–^, Figure 7a). Besides the electrostatic attraction between the tris-carboxylate anion and the P-1 piperazin-1-ium melamine G-1 cations, the occurrence of important H-bonding three interactions (>C
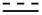
O^δ(b)–^···H(ax or eq)-NH^+^<) were identified, being supported by the bond lengths decreasing order as 1.265 Å (d(C
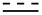
O^δ(b)–^) in **10**) > 1.252 Å (d(C
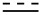
O^δ–^) in trimesic tris-carboxylate anion) > 1.244 Å (d(C
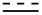
O^δ(a)–^) in **10**) together with Σ*r*_cov_ (O, H): 1.40 Å < d(O^δ(b)–^···H): 1.60 Å < Σ*r*_wdW_ (O, H): 2.60 Å [70]. One must observe that, contrarily to our planned design of **7b**, as [**D-N<P>NH^2+^**]_3_(^–^OOC)_3_C_6_H_3_ (Figure 1, linear connection, as a six-membered chelate, to the *m*-trivalent core), the H-bonding network in model **10** determined simpler angular connections of the P-1 piperazin-1-ium melamine G-1 cations around the trimesic tris-carboxylate anion core in a statistically favoured *asymmetric* manner (Figure 1). In spite of this local “irregularity”, entirely re-found in the optimised geometry of **7b** (Figure 7), its global profile was of type *propeller* due to the adopted vault shape of its three G-1 branches (**D**, Figure 1), i.e., concavity vs convexity.

Extrapolation of the ionic relationships found for model **10** to compounds with higher mass **8** and **9** (Figure 8) provided their energetic minima as to correspond to (i) a local *propeller* orientation of the G-1 branches around the *s*-triazin-2,4,6-triyl core (initial design, Figure 1) and (ii) a global vaulted form (“candelabrum” allure) promoted, as in the case of **7b**, by the same arched G-1 branches.

#### 3.4 Assignments by means of TEM analyses

**Figure 9:**
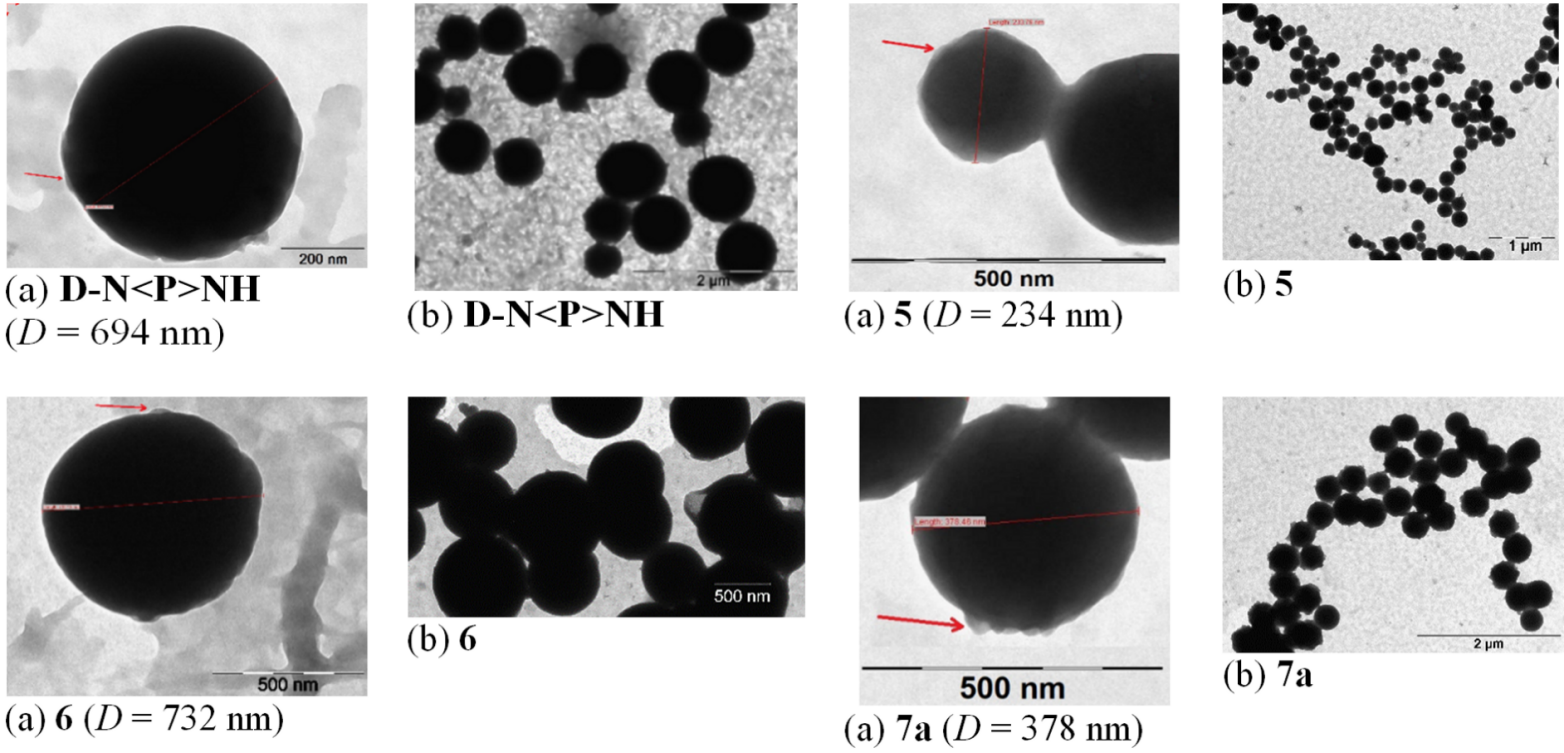
TEM images of homogeneously packed spherical nano-aggregates (a) and their agglomerations (b) in the case of G-1 amino-dendron **D-N<P>NH** and of covalent G-2 dendrimers **5–7a**. *D* is the average diameter of nanospheres; the red arrow shows the nano-particles that aggregate.

**Figure 10:**
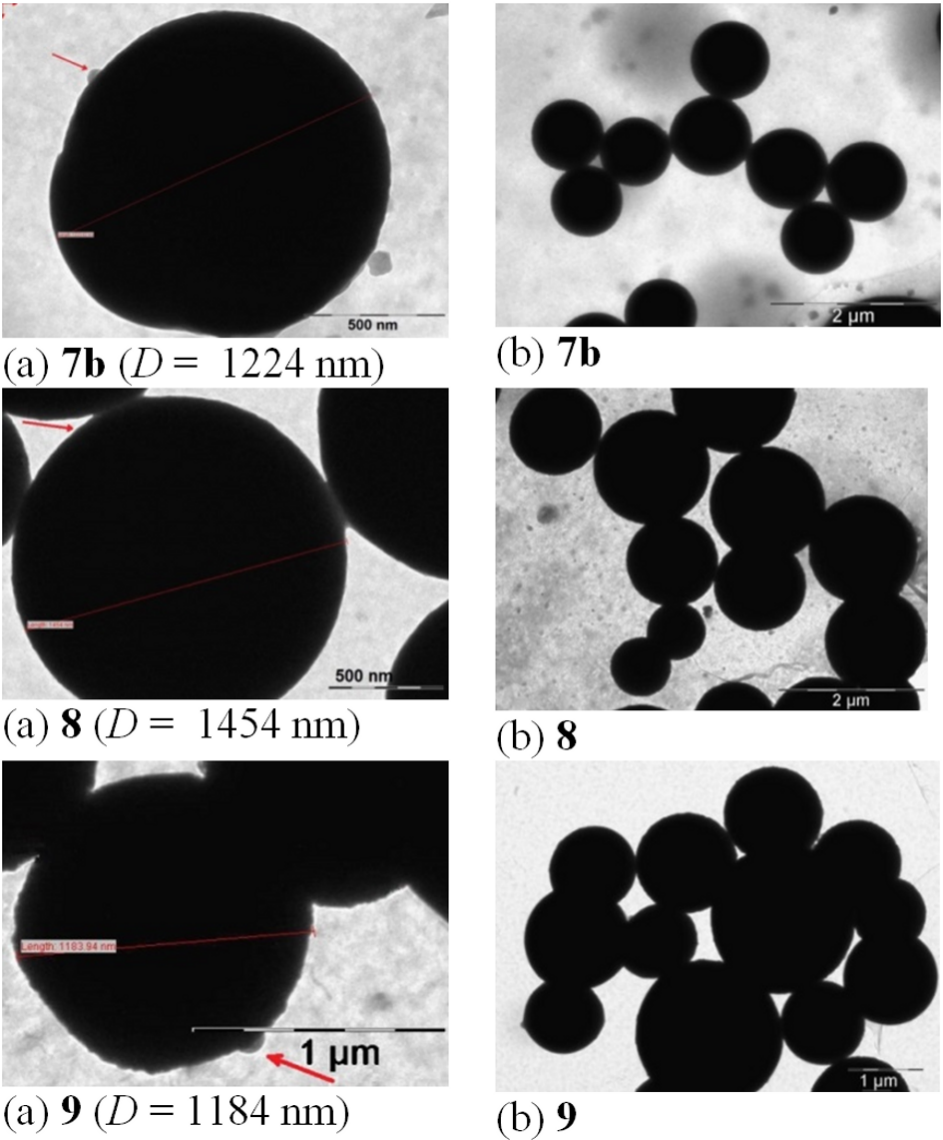
TEM images of homogeneously packed spherical nano-aggregates (a) and their agglomerations (b) in the case of G-2 ionic dendrimers **7b**, **8** and **9**; the red arrow indicates the nano-particles that aggregate. *D* is the average diameter of nanospheres.

We also considered of interest to corroborate our ab initio study with an introductory exploration by means of TEM (transmission electronic microscopy). In this purpose, the sampling was made by dissolving compounds **D-N<P>NH** and **4–9** (ca. 1 mg) in DMSO (1 mL, see section 3.1) under sonication. One drop of solution was deposited on a Formvar/Carbon coated copper grid (300 mesh) and let to free evaporate to dryness at room temperature (24 h). Except for compound **4**, agglomerations of homogeneously packed spherical nano-aggregates (Figure 9 and Figure 10) were thus obtained. They were comparable with those of the recently reported G-2 PAMAM ionic (–COO^−^/H_3_N^+^–) dendrimers, already mentioned [63]. Each sample preparation was repeated three times and the hereafter discussed numerical data were mediated.

**Figure 11:**
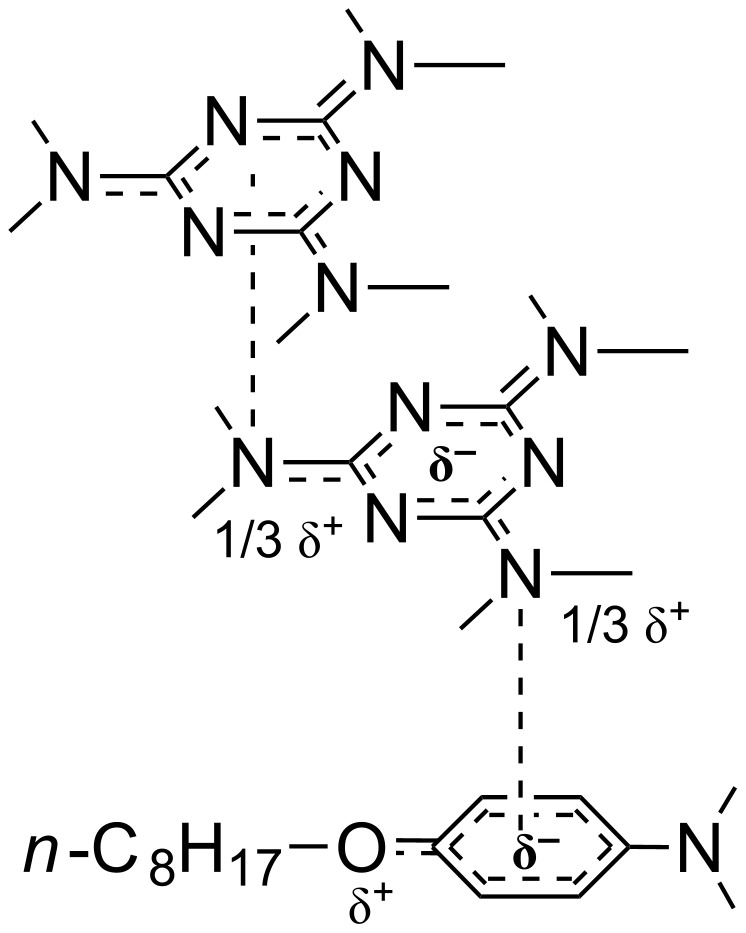
Proposed π-stacking interactions in compounds **D-N<P>NH** and **5**–**7a**.

The size of nanospheres (expressed by their average diameters, *D*) ranged in a large domain (234–1454 nm) with a quite wide distribution of *D* values in all situations (Supporting Information File 1, p. S41, Figure S55). With the exception of compound **6**, all other G-2 dendrimers were, in fact, trimers of the same G-1 dendron, **D-N<P>NH**. Therefore, for the present discussion, we will limit our assignments to the structural diversity impact of the central building blocks (or cores) together with that of the dendritic elaboration (covalent vs ionic), seen both accountable for the observed different propensity for self-assembly as nano-aggregates. Indeed, covalent dendrimers **5–7a** generated nanospheres with much smaller average diameter (259–732 nm, Figure 9) than the ionic **7b–9** (992–1454 nm, Figure 10). Moreover, according to the literature [71], the last ones appeared to be among the greatest previously reported nanosystems as polymeric nanoparticles. In covalent series, G-1 dendron **D-N<P>NH** itself (as unexpected reference) self-assembled into nanospheres with a high *D* value (694 nm). We ascribed this packing ability to three basic π stacking interactions [72] associating stratified parallel-displaced dendritic scaffolds (Figure 11):

(i) (π–π) between the electron-poor *N*-atoms, adjacent to *s*-triazine rings, as π-acceptors, from one hand, and the π-enriched (hetero)cycles, *s*-triazines and 4-(*n*-octyloxy)phenylamino peripheral units, on the other hand, as π-donors.

(ii) (π–H) of type (*s*-triazine) adjacent >N^sp2^–H···π-enriched (hetero)cycles.

Surprisingly, single trimer **4** ([**D-N<P>N**]_3_C_3_N_3_, Figure 1), containing *s*-triazine as linearly linked core, gave no distinct spherical aggregation (Supporting Information File 1, p. S41, Figure S56). In contrast, by replacing the P-1 piperazine linker in **4** with a 4-oxyphenylamino unit, the resulting dendrimer **6** ([**D_3_**]**B**, Figure 1), still encompassing *s*-triazine as core but *propeller* angularly coupled, evidenced a total opposite behaviour, i.e., aggregation under the form of nanospheres with the highest (732 nm) D value.

Trimers of **D-N<P>NH** angularly-*propeller* attached to 1,3,5-trisubstituted benzene cores, as π-electron-enhanced (1,3,5-tris(methylene)benzene, [**D**]_3_**A** (Figure 1), compound **5**, *D* = 234 nm) or π-poor (1,3,5-tris(formyl)benzene, [**D-N<P>N**]_3_(OC)_3_C_6_H_3_ (Figure 1), compound **7a**, *D* = 378 nm) self-assembled into the smallest spherical nano-aggregates.

By far, due to their electrostatic interactions (attractions vs repulsions) G-2 ionic dendrimers **7b**, **8** and **9** (Figure 10) produced the most developed spherical nano-aggregates. The ionic relationships masked those deduced in covalent series, namely the π–π and π–H stacking and angular-*propeller* connectivity of the G-1 dendritic branches to the *m*-trivalent core.

## Conclusion

Starting from 4-(*n*-octyloxy)aniline, the five-step orthogonal convergent synthesis of a new seven terms series of G-2 melamine based-dendrimers was achieved in overall yields ranging between 29 and 79%. By means of DFT calculation in solution, the reaction conditions were found mandatory to the high solvation of the G-0 and G-1 dendrons exhibiting a major (*anti–anti*) (parallel) rotamerism of the peripheral 4-(*n*-octyloxy)phenyl units about the C(*s*-triazine)–N(exocyclic) partial double bonds. The final iterative synthetic step was realised by covalent and/or (carboxyl/amino) ionic trimerisations, to recommend two G-1 *N*-substituted melamine dendrons with piperzine-1,4-diyl (linkers) and 4-(*n*-octyloxyphenyl)amino (peripheral units), **D-Cl** and **D-N<P>NH**, as promising scaffolds for future dendritic elaborations.

Tandem DFT-(VT) NMR investigations revealed (i) the regular shape in solution of the terminal 4-(*n*-octyloxy)phenyl units (“parallel”, *anti–anti*), (ii) the *propeller* arrangement, in the case of the angular connections of G-1 dendrons around the *m*-trivalent core, (iii) the vaulted shapes of G-2 dendrimers and (iv) in one case, the occurrence of a starburst effect. The “salt”-like nature of the G-2 dendrimers (obtained by a carboxyl/amino 1:3 stoichiometric trimerisation) could be unambiguously assigned by means of ^1^H NMR (in solution) and IR (solid state), confirming the existence of the only tris-carboxylate anions. TEM analysis indicated the aptitude of our G-2 vaulted melamines for π–π and π–H stacking self-assembly into homogeneously packed spherical nano-aggregates. Their size was tailored primarily by the covalent vs ionic nature of dendrimers, i.e., the last ones producing nanospheres with more than 1000 nm averaged *D* values. The structural variety of the covalent dendritic elaboration around the *m*-trivalent cores (1,4-phenylene over piperazin-1,4-diyl adjacent linkers, *propeller* over *asymmetric* rotamerism of the angular over linear connectivity) modulated the degree of nano-aggregation.

## Experimental

### General

All reagents and solvents were of commercial quality and required no purification prior to use. Melting points were carried out on an ELECTROTHERMAL instrument and were not corrected. Microanalyses were performed on a Carlo Erba CHNOS 1160 apparatus.

TLC monitoring was performed by using aluminium sheets with silica gel 60 F_254_ (Merck) (visualisation under UV at λ = 254 nm).

Column chromatography was conducted on silica gel Si 60 (0.063–0.200 mm, Merck).

IR spectra were recorded on a JASCO FTIR 6100 Spectrometer. Only relevant absorption maxima (in cm^−1^) are listed throughout as being s (strong), m (medium) or w (weak).

NMR spectra were recorded on a Bruker AM 500 instrument operating at 500 or 125 MHz for ^1^H and ^13^C nuclei, respectively. All chemical shifts (δ values) are given in parts per million (ppm); all homocoupling patterns (*^n^J*_H,H_ values) are given in Hertz. In the NMR descriptions, some specific abbreviations were used: “br s” (broad singlet), “br t” (broad triplet), “br m” (broad multiplet), “dd” (doublet of doublets), “tt app. q” (triplet of triplets app. as a quartet), “tt app. qi” (triplet of triplets app. as a quintet), “tt app. s” (triplet of triplets app. as a sextet), “tt app. sp” (triplet of triplets app. as a septet), “ddd app. td” (doublet of doublets of doublets app. as a triplet of doublets), T-0, T-1 and T-2 (*s*-triazine branch cell according to generations 0, 1 and 2), P-0 and P-1 (piperazine as linker according to generations 0 and 1).

Data for 2D-^1^H-DOSY NMR spectra of compounds **D-N<P>NH**, **4**, **5**, **7–9** (2.5 or 5 mM in DMSO-*d*_6_ at 298 K on 500 MHz timescale) were acquired by means of the ledbpgp2s pulse sequence. The diffusion time (Δ) ranged from 150 to 220 ms and the gradient pulse length (δ) ranged from 1.7 to 1.9 ms. The size of the raw data set was 32 × 16 k. The gradient intensity values were equally spaced from 2% to 95%. The DOSY spectrum was calculated using the Bruker TOPSPIN Software. The inverse Laplace transformation in the indirectly detected dimension was carried out by means of the MaxEnt algorithm. Log (*D*) was calculated with *D* expressed in µm^2^ s^−1^: **D-N<P>NH** (5 mM, δ = 1.7 ms, Δ = 220 ms), **4** (2.5 mM, δ = 1.8 ms, Δ = 150 ms), **5** (5 mM, δ = 1.9 ms, Δ = 190 ms), **7a** (2.5 mM, δ = 1.7 ms, Δ = 150 ms), **7b** (2.5 mM, δ = 1.8 ms, Δ = 150 ms), **8** (5 mM, δ = 1.9 ms, Δ = 150 ms), **9** (5 mM, δ = 1.7 ms, Δ = 150 ms).

Mass spectra were obtained on an LTQ ORBITRAP XL (Thermo Scientific) instrument which was externally calibrated using the manufacturer’s ESI(+) calibration mix. The samples were introduced into the spectrometer by direct infusion.

### DFT calculations

The full geometry optimisation of compounds **2a**, **3**, **7a** and **7b** were carried out using DFT level of theory considering the M06-2X exchange-correlation functional together with the def2-TZVP basis set in the presence of a solvent environment implemented in the Gaussian 09 program package. The solvent effects were taken into account via the Polarisable Continuum Model (PCM) using the integral equation formalism variant (IEFPCM) and considering DMSO (ε = 46.826) as the solvent environment; the total electronic energies of the *anti–anti* conformations were taken as the reference value.

Free energies of solvation (Δ*G*) of compounds **2a**, **3**, **D-Cl** and **D-N<P>NH** were calculated at the M06-2X/def2-TZVP level of theory (solvent model: THF and 1,4-dioxane) through the SMD solvation model of Truhlar.

The LP electron occupation of piperazine N^sp3^ nitrogen in compounds **3** and **D-N<P>NH** was obtained by considering the NBO electron population analysis at the M06-2X/def2-TZVP level of theory, where *e* is the elementary electric charge carried by a single electron.

The molecular graphics (figures) were created by using GaussView software.

### TEM analysis

TEM images were obtained with a Hitachi H-7650 transmission electron microscope operating at 80 keV.

## Supporting Information

File 1 All procedures for the synthesis of compounds **2a**, **2b**, **3**, **D-Cl**, **D-N<P>NH**, **4-9** together with their full analytical data, (VT) NMR and MS spectra.
