# Supplementary material for: Design, synthesis and structure of novel G-2 melamine-based dendrimers incorporating 4-(n-octyloxy)aniline as a peripheral unit
Source: Beilstein J Org Chem. 2018 Jul 9;14:1704–22. doi: 10.3762/bjoc.14.145 (PMC6071710; doi:10.3762/bjoc.14.145)
Supplement: File 1 — All procedures for the synthesis of compounds 2a, 2b, 3, D-Cl, D-NNH, 4-9 together with their full analytical data, (VT) NMR and MS spectra. [file Beilstein_J_Org_Chem-14-1704-s001.pdf]

Supporting Information
for

Design, synthesis and structure of novel G-2 melamine-based dendrimers incorporating 4-(*n*-octyloxy)aniline as a peripheral unit

Cristina Morar[1], Pedro Lameiras[2], Attila Bende[3], Gabriel Katona[4], Emese Gál[4] and Mircea Darabantu[*,§,1]

Address: [1]Department of Chemistry, Babes-Bolyai University, 11 Arany János St., 400028 Cluj-Napoca, Romania, [2]University of Reims Champagne-Ardenne, ICMR, UMR 7312, BP 1039, 51687 Reims, France, [3]National Institute for Research and Development of Isotopic and Molecular Technologies, 67-103, Donath St., PO Box 700, 400293 Cluj-Napoca 5, Romania and [4]Department of Chemistry and Chemical Engineering, Hungarian Line of Study, Babes-Bolyai University, 11 Arany János St., 400028 Cluj-Napoca, Romania
*Corresponding Author
Email: Mircea Darabantu - darab@chem.ubbcluj.ro
[§]Tel. +40 264 59 38 33; Fax. +40 264 59 08 18

All procedures for the synthesis of compounds 2a, 2b, 3, D-Cl, D-N<P>NH, 4–9 together with their full analytical data, (VT) NMR and MS spectra

Overview pag.

Preparation of compounds **2a** *and* **2b** *by Williamson etherification of compound* **1** *with 1-iodooctane* S3
Preparation of compound **2a** *by amination of cyanuric chloride with 4-(n-octyloxy)aniline* S3
2-Chloro-4,6-bis[4-(n-octyloxy)phenylamino]-s-triazine **2a** S3
2-Chloro-4-{N-(n-octyl)-N-[4-(n-octyloxy)phenyl]}amino-6-[4-(n-octyloxy)phenylamino]-s-triazine **2b** S3
Preparation of compound **3** S4
2-(Piperazin-1-yl)-4,6-bis[4-(n-octyloxy)phenylamino]-s-triazine **3** S4
Preparation of G-1 dendron **D-Cl** S5
2-Chloro-4,6-bis{4-{4,6-bis[4-(n-octyloxy)phenylamino]-s-triazin-2-yl}-piperazin-1-yl}-s-triazine **D-Cl** S5
Preparation of G-1 dendron **D-N<P>NH** S5
2-(Piperazin-1-yl)-4,6-bis{4-{4,6-bis[4-(n-octyloxy)phenylamino]-s-triazin-2-yl}-piperazin-1-yl}-s-triazine **D-N<P>NH** S6
Preparation of G-2 dendrimer **4** S6
2,4,6-Tris{4-{4,6-bis{4-{4,6-bis[4-(n-octyloxy)phenylamino]-s-triazin-2-yl}-piperazin-1-yl}-s-triazin-2-yl}-piperazin-1-yl}-s-triazine **4** S6
Preparation of G-2 dendrimer **5** S7
1,3,5-Tris{{4-{4,6-bis{4-{4,6-bis[4-(n-octyloxy)phenylamino]-s-triazin-2-yl}-piperazin-1-yl}-s-triazin-2-yl}-piperazin-1-yl}methyl}benzene **5** S7
Preparation of G-2 dendrimer **6** S7
2,4,6-Tris{4-{4,6-bis{4-{4,6-bis[4-(n-octyloxy)phenylamino]-s-triazin-2-yl}-piperazin-1-yl}-s-triazin-2-oxy}-phenylamino}-s-triazine **6** S8
Preparation of G-2 dendrimer **7a** S8
1,3,5-Tris{4-{4,6-bis{4-{4,6-bis[4-(n-octyloxy)phenylamino]-s-triazin-2-yl}-piperazin-1-yl}-s-triazin-2-yl}-piperazin-1-ylcarbonyl}benzene **7a** S9
Preparation of G-2 dendrimer **7b** S9
Tris{4-{4,6-bis{4-{4,6-bis[4-(n-octyloxy)phenylamino]-s-triazin-2-yl}-piperazin-1-yl}-s-triazin-2-yl}-piperazin-1-ium}-1,3,5-benzene tricarboxylate **7b** S9
Preparation of G-2 dendrimer **8** S10
Tris{4-{4,6-bis{4-{4,6-bis[4-(n-octyloxy)phenylamino]-s-triazin-2-yl}-piperazin-1-yl}-s-triazin-2-yl}-piperazin-1-ium} 2,4,6-tris[4-(methoxyl)phenylamino]-s-triazine tricarboxylate **8** S10
Preparation of G-2 dendrimer **9** S10
Tris{4-{4,6-bis{4-{4,6-bis[4-(n-octyloxy)phenylamino]-s-triazin-2-yl}-piperazin-1-yl}-s-triazin-2-yl}-piperazin-1-ium} 2,4,6-tris[4-(prop-1-yloxy)]phenylamino-s-triazine tricarboxylate **9** S10
Figure S1: ^{1}H NMR spectrum of compound **2a** (500 MHz, 5.0 mM in DMSO-d_6, 298 K) S12
Figure S2: ^{1}H NMR spectrum of compound **2a** (500 MHz, 5.0 mM in DMSO-d_6, 363 K) S12
Figure S3: DEPT ^{13}C NMR spectrum of compound **2a** (125 MHz, 5.0 mM in DMSO-d_6, 298 K) S13
Figure S4: 2D-^{1}H-DOSY NMR chart of compound **2a** (500 MHz, 5.0 mM in DMSO-d_6, 298 K) S13

Figure S5: Mass spectrum of compound **2a** [HRMS (APCI+)] S14
Figure S6: ^{1}H NMR spectrum of compound **2b** (500 MHz, 5.0 mM in DMSO-d_6, 298 K) S14
Figure S7: ^{1}H NMR spectrum of compound **2b** (500 MHz, 5.0 mM in DMSO-d_6, 403 K) S15
Figure S8: DEPT ^{13}C NMR spectrum of compound **2b** (125 MHz, 5.0 mM in DMSO-d_6, 298 K) S15
Figure S9: 2D-^{1}H-DOSY NMR chart of compound **2b** (500 MHz, 5.0 mM in DMSO-d_6, 298 K) S17
Figure S10: Mass spectrum of compound **2b** [HRMS (APCI+)] S16
Figure S11: ^{1}H NMR spectrum of compound **3** (500 MHz, 5.0 mM in DMSO-d_6, 298 K) S17
Figure S12: ^{1}H NMR spectrum of compound **3** (500 MHz, 5.0 mM in DMSO-d_6, 363 K) S17
Figure S13: DEPT ^{13}C NMR spectrum of compound **3** (125 MHz, 5.0 mM in DMSO-d_6, 298 K) S18
Figure S14: 2D-^{1}H-DOSY NMR chart of compound **3** (500 MHz, 5.0 mM in DMSO-d_6, 298 K) S18
Figure S15: Mass spectrum of compound **3** [HRMS (APCI+)] S19
Figure S16: ^{1}H NMR spectrum of compound **D-Cl** (500 MHz, 5.0 mM in DMSO-d_6, 363 K) S19
Figure S17: DEPT ^{13}C NMR spectrum of compound **D-Cl** (125 MHz, 5.0 mM in DMSO-d_6, 298 K) S20
Figure S18: 2D-^{1}H-DOSY NMR chart of compound **D-Cl** (500 MHz, 5.0 mM in DMSO-d_6, 298 K) S20
Figure S19: Mass spectrum of compound **D-Cl** [HRMS (ESI+), ACN+TFA] S21
Figure S20: ^{1}H NMR spectrum of compound **D-N<P>NH** (500 MHz, 5.0 mM in DMSO-d_6, 298 K) S21
Figure S21: ^{1}H NMR spectrum of compound **D-N<P>NH** (500 MHz, 5.0 mM in DMSO-d_6, 363 K) S22
Figure S22: DEPT ^{13}C NMR spectrum of compound **D-N<P>NH** (125 MHz, 5.0 mM in DMSO-d_6, 298 K) S22
Figure S23: 2D-^{1}H-DOSY NMR chart of compound **D-N<P>NH** (500 MHz, 5.0 mM in DMSO-d_6, 298 K) S23
Figure S24: Mass spectrum of compound **D-N<P>NH** [HRMS (APCI+)] S23
Figure S25: ^{1}H NMR spectrum of compound **4** (500 MHz, 2.5 mM in DMSO-d_6, 298 K) S24
Figure S26: ^{1}H NMR spectrum of compound **4** (500 MHz, 2.5 mM in DMSO-d_6, 363 K) S24
Figure S27: ^{13}QC NMR of compound **4** (125 MHz, 2.5 mM in DMSO-d_6, 363 K) S25
Figure S28: 2D-^{1}H-DOSY NMR chart of compound **4** (500 MHz, 2.5 mM in DMSO-d_6, 298 K) S25
Figure S29: Mass spectrum of compound **4** [HRMS (ESI+) ACN+TFA] S26
Figure S30: ^{1}H NMR spectrum of compound **5** (500 MHz, 5.0 mM in DMSO-d_6, 298 K) S26
Figure S31: ^{1}H NMR spectrum of compound **5** (500 MHz, 5.0 mM in DMSO-d_6, 363 K) S27
Figure S32: ^{13}QC NMR of compound **5** (125 MHz, 5.0 mM in DMSO-d_6, 298 K) S27
Figure S33: 2D-^{1}H-DOSY NMR chart of compound **5** (500 MHz, 5.0 mM in DMSO-d_6, 298 K) S28
Figure S34: Mass spectrum of compound **5** [HRMS (ESI+), ACN+TFA] S28
Figure S35: ^{1}H NMR spectrum of compound **6** (500 MHz, 2.5 mM in DMSO-d_6, 363 K) S29
Figure S36: ^{13}QC NMR of compound **6** (125 MHz, 2.5 mM in DMSO-d_6, 363 K) S29
Figure S37: Mass spetrum of compound **6** [HRMS (ESI+), ACN+TFA] S30
Figure S38: ^{1}H NMR spectrum of compound **7a** (500 MHz, 2.5 mM in DMSO-d_6, 298 K) S30
Figure S39: ^{1}H NMR spectrum of compound **7a** (500 MHz, 2.5 mM in DMSO-d_6, 363 K) S31
Figure S40: ^{13}QC NMR spectrum of compound **7a** (125 MHz, 5.0 mM in DMSO-d_6, 298 K) S31
Figure S41: 2D-^{1}H-DOSY NMR chart of compound **7a** (500 MHz, 2.5 mM in DMSO-d_6, 298 K) S32
Figure S42: Mass spectrum of compound **7a** [HRMS (ESI+), ACN+TFA] S32
Figure S43: ^{1}H NMR spectrum of compound 7**b** (500 MHz, 2.5 mM in DMSO-d_6, 298 K) S33
Figure S44: ^{1}H NMR spectrum of compound 7**b** (500 MHz, 2.5 mM in DMSO-d_6, 363 K) S33
Figure S45: ^{13}QC NMR spectrum of compound **7b** (125 MHz, 2.5 mM in DMSO-d_6, 298 K) S34
Figure S46: 2D-^{1}H-DOSY NMR chart of compound 7**b** (500 MHz, 2.5 mM in DMSO-d_6, 298 K) S34
Figure S47: ^{1}H NMR spectrum of compound **8** (500 MHz, 5.0 mM in DMSO-d_6, 298 K) S35
Figure S48: ^{1}H NMR spectrum of compound **8** (500 MHz, 5.0 mM in DMSO-d_6, 363 K) S35
Figure S49: ^{13}QC NMR spectrum of compound **8** (125 MHz, 5.0 mM in DMSO-d_6, 298 K) S36
Figure S50: 2D-^{1}H-DOSY NMR chart of compound **8** (500 MHz, 5.0 mM in DMSO-d_6, 298 K) S36
Figure S51: ^{1}H NMR spectrum of compound **9** (500 MHz, 5.0 mM in DMSO-d_6, 298 K) S37
Figure S52: ^{1}H NMR spectrum of compound **9** (500 MHz, 5.0 mM in DMSO-d_6, 363 K) S37
Figure S53: ^{13}QC NMR spectrum of compound **9** (125 MHz, 5.0 mM in DMSO-d_6, 298 K) S38
Figure S54: 2D-^{1}H-DOSY NMR chart of compound **9** (500 MHz, 5.0 mM in DMSO-d_6, 298 K) S38
Extracted data from the output files of ΔG *of solvation calculation for compounds* **2a**, **3**, **D-Cl** *and* **D-N<P>NH** S39
Figure S55: Spherical nano-aggregates diameter distributions of compounds **D-N<P>NH** and **5-9** (from DMSO) S41
Figure S56: TEM image of compound **4** (from DMSO) S42

Preparation of compounds **2a** *and* **2b** *by Williamson etherification of compound* **1** *with 1-iodooctane*

Under inert atmosphere and with vigorous stirring, into an anhyd. acetone (40 mL) solution containing compound **1** (1.000 g, 3.04 mmol), anhyd. K_2CO_3 (3.350 g, 24.32 mmol) was suspended. To this suspension, an anhyd. acetone (5 mL) solution containing 1-iodooctane (0.865 g, 0.65 mL, 3.60 mmol) was added dropwise (1 h) at −13 °C. The reaction mixture was stirred at −13 °C for 2 h then for 12 h at room temperature (TLC monitoring eluent ligroin/acetone 4:1). Furthermore, an anhyd. acetone (5 mL) solution containing 1-iodooctane (0.865 g, 0.65 mL, 3.60 mmol) was injected and the reaction mixture was stirred at room temperature for additional 72 h. Finally, the reaction mixture was heated at 50 °C for 8 h. After cooling at room temperature, chloroform (50 mL) and distilled water (50 mL) were added with stirring. The resulted two layers were separated and the organic phase was washed with distilled water (2 × 25 mL), dried over anhyd. Na_2SO_4, filtered off and evaporated to dryness under reduced pressure. The solid residue was separated by column chromatography to provide pure compound **2a** (0.688 g, 41% partial conversion of **1**) as the first fraction. Next elution afforded pure compound **2b** (0.143 g, 7% partial conversion of **1**).

Preparation of compound **2a** *by amination of cyanuric chloride with 4-(n-octyloxy)aniline*

Under inert atmosphere and with vigorous stirring, to an anhyd. acetone (10 mL) solution containing cyanuric chloride (0.625 g, 3.39 mmol), an anhyd. acetone (26 mL) solution containing 4-(*n*-octyloxy)aniline (1.500 g, 6.78 mmol) was injected dropwise at 0–5 °C during 2 h. At the same temperature, a distilled water (8 mL) solution containing $NaHCO_3$ (0.569 g, 6.78 mmol) was then added. The resulted suspension was heated at 45 °C for 3 h then let to cool down to room temperature at which it was kept for additional 18 h with continuous stirring. After this period, TLC monitoring (eluent ligroin/acetone 4:1) indicated the complete consumption of the starting materials and formation of compound **2a** as a major spot. The reaction mixture was evaporated under reduced pressure to the complete removal of acetone. Distilled water (30 mL) was added and the resulted suspension was filtered off. After drying at room temperature, the crude product (1.85 g) was crystallised from boiling methanol (9 mL) to provide pure compound **2a** (1.760 g, 94% yield with respect to cyanuric chloride).

2-Chloro-4,6-bis[4-(n-octyloxy)phenylamino]-s-triazine (**2a**). White powder; m.p. 149-151 ^{o}C (MeOH). Yield 94%. R_f=0.58 (ligroin/acetone 4:1). Elemental analysis calcd (%) for $C_{31}H_{44}ClN_5O_2$: C 67.19, H 8.00, N 12.64; found: C 66.98, H 7.97, N 12.55. IR (KBr) ν_{max} 3347 (m), 3255 (m), 2958 (m), 2921 (s), 2852 (m), 1628 (m), 1573 (s), 1544 (s), 1525 (s), 1502 (s), 1419 (m), 1384 (m), 1212 (s), 1015 (m), 829 (m), 793 (m) cm^{-1}. ^{1}H and 2D-^{1}H,^{1}H-COSY NMR (500 MHz, 5.0 mM in DMSO-d_6, 298 K): δ_H=0.86 (6H, t, $^3J_{H,H}$=6.8 Hz; CH_3), 1.26-1.35 (16H, m; CH_2, *n*-octyl), 1.40 (4H, tt app. qi, $^3J_{H,H}$=7.5 Hz; CH_2, *n*-octyl), 1.69 (4H, tt app. qi, $^3J_{H,H}$=6.8 Hz; OCH_2CH_2, *n*-octyl), 3.93 (4H, t, $^3J_{H,H}$=6.5 Hz; OCH_2, *n*-octyl), 6.87 (4H, d, $^3J_{H,H}$=8.0 Hz; H-3, -5, Ph), 7.46, 7.65 (4H, d, br s, $^3J_{H,H}$=8.0 Hz; H-2, -6, Ph), 9.85, 9.96, 10.03 (2H, br s, br s, s; NH) ppm. ^{1}H and 2D-^{1}H,^{1}H-COSY NMR (500 MHz, 5.0 mM in DMSO-d_6, 363 K): δ_H=0.88 (6H, t, $^3J_{H,H}$=7.0 Hz; CH_3), 1.27-1.38 (16H, m; CH_2, *n*-octyl), 1.44 (4H, tt app. qi, $^3J_{H,H}$=7.0 Hz; CH_2, *n*-octyl), 1.72 (4H, tt app. qi, $^3J_{H,H}$=6.9 Hz; OCH_2CH_2, *n*-octyl), 3.97 (4H, t, $^3J_{H,H}$=6.8 Hz; OCH_2, *n*-octyl), 6.87 (4H, d, $^3J_{H,H}$=9.0 Hz; H-3, -5, Ph), 7.49 (4H, d, $^3J_{H,H}$=9.0 Hz; H-2, -6, Ph), 9.63 (2H, br s; NH) ppm. 2D-^{1}H-DOSY NMR (500 MHz, 5.0 mM in DMSO-d_6, 298 K): D=198 μm^2 s^{-1}. DEPT ^{13}C NMR (125 MHz, 5.0 mM in DMSO-d_6, 298 K): δ_C=14.4 (CH_3), 22.6 (CH_2CH_3), 26.0, 29.16, 29.19, 29.3, 31.7 (CH_2; *n*-octyl), 68.1 (OCH_2; *n*-octyl), 114.7, 114.8 (C-3, -5; Ph), 122.5, 123.3 (C-2, -6; Ph), 131.6, 132.0 (C-1; Ph), 155.3, 155.5 (C-4; Ph), 163.7, 164.2, 164.4 (C-4, -6; *s*-triazine), 168.2, 168.7 (C-2; *s*-triazine) ppm. HRMS-APCI (rel. int.): *m*/*z* (%) 554.3246 (100) $[M+H]^+$; $[M+H]^+$ calcd. for $C_{31}H_{45}ClN_5O_2$, 554.3262.

2-Chloro-4-{N-(n-octyl)-N-[4-(n-octyloxy)phenyl]}amino-6-[4-(n-octyloxy)phenylamino]-s-triazine (**2b**).

White powder; m.p. 74-75 °C (column chromatography, eluent ligroin/acetone 4:1; cryst. from MeOH). R_f=0.76. Partial conversion of **1**, 7%. Elemental analysis calcd (%) for $C_{39}H_{60}ClN_5O_2$: C 70.29, H 9.08, N 10.51; found: C 69.98, H 9.27, N 10.55. IR (KBr) ν_{max} 3258 (m), 2960 (s), 2921 (s), 2853 (s), 1575 (s), 1530 (s), 1505 (s), 1484 (s), 1390 (m), 1375 (m), 1248 (s), 1230 (s), 983 (m), 830 (m), 796 (m) cm^{-1}. ^{1}H and 2D-^{1}H,^{1}H-COSY NMR (500 MHz, 5 mM in DMSO-d_6, 298 K): δ_H=0.80-0.86 (9H, m; CH_3), 1.19-1.78 (36H, m; CH_2, *n*-octyl), 3.80 (2H, t, $^3J_{H,H}$=6.0 Hz; NCH_2, *n*-octyl), 3.85, 3.92, 3.97, 4.02 (4H, t, $^3J_{H,H}$=7.0, 6.5, 6.3, 6.3 Hz; OCH_2, *n*-octyl), 6.50, 6.87, 6.95, 7.03 (4H, d, $^3J_{H,H}$=8.5, 8.5, 9.0, 8.5 Hz; Ph), 7.16, 7.20, 7.35, 7.44, 7.61 (4H, d, $^3J_{H,H}$=8.0, 8.5 Hz, br s, br s, d, $^3J_{H,H}$=8.5 Hz; Ph), 9.43, 9.76, 9.89, 10.02 (1H, br s, br s, s, s; NH) ppm. ^{1}H and 2D-^{1}H,^{1}H-COSY NMR (500 MHz, 5.0 mM in DMSO-d_6, 403 K): δ_H=0.87-0.90 (9H, br m; CH_3), 1.27-1.50 (30H, br m; CH_2, *n*-octyl), 1.60 (2H, br t; CH_2, *n*-octyl), 1.71 (2H, br t, $^3J_{H,H}$=6.0 Hz; CH_2, *n*-octyl), 1.78 (2H, br t, $^3J_{H,H}$=5.8 Hz; CH_2, *n*-octyl), 3.87 (2H, t, $^3J_{H,H}$=6.5 Hz; NCH_2, *n*-octyl), 3.93 (2H, t, $^3J_{H,H}$=4.5 Hz; OCH_2, *n*-octyl), 4.05 (2H, t, $^3J_{H,H}$=5.0 Hz; OCH_2, *n*-octyl), 6.72 (2H, d, $^3J_{H,H}$=5.8 Hz; H-2, -6, Ph at C-4 *s*-triazine), 6.98 (2H, d, $^3J_{H,H}$=7.0 Hz; H-3, -5, Ph at C-6 *s*-triazine), 7.16 (2H, d, $^3J_{H,H}$=7.3 Hz; H-3, -5, Ph at C-4 *s*-triazine), 7.36 (2H, dd, $^3J_{H,H}$=7.0 Hz; H-2, -6, Ph at C-6 *s*-triazine), 9.25 (1H, br s; NH) ppm. 2D-^{1}H-DOSY NMR (500 MHz, 5.0 mM in DMSO-d_6, 298 K): *D*=197 μm^2 s^{-1}. DEPT ^{13}C NMR (125 MHz, 5.0 mM in DMSO-d_6, 298 K): δ_C=14.35, 14.41 (CH_3), 22.5, 22.6 (CH_2CH_3), 26.02, 26.05, 26.1, 26.4, 26.8, 27.2, 27.3, 27.6 (CH_2; *n*-octyl), 29.0, 29.1, 29.19, 29.2, 29.3, 29.4 (CH_2; *n*-octyl), 49.3, 49.34, 49.36, 51.4 (NCH_2; *n*-octyl), 67.8, 67.97, 68.02, 68.06, 68.1, 68.3, 68.4 (OCH_2; *n*-octyl), 114.1, 114.2, 114.7, 114.8 (C-3, -5; Ph at C-6 *s*-triazine), 115.2, 115.3, 115.4 (C-3, -5; Ph at C-4, *s*-triazine), 121.21, 122.17, 122.3 (C-2, -6; Ph at C-6 *s*-triazine), 129.2, 129.6 (C-2, -6; Ph at C-4 *s*-triazine), 132.2, 132.3 (C-1; Ph at C-6 *s*-triazine), 135.0, 135.1, 135.2 (C-1; Ph at C-4 *s*-triazine), 154.5, 154.6, 155.2, 155.3 (C-4; Ph at C-6 *s*-triazine), 157.8, 158.0 (C-4; Ph at C-4 *s*-triazine), 162.6, 162.7, 163.7, 163.8 (C-6; *s*-triazine), 165.4, 165.5, 165.9 (C-4; *s*-triazine), 168.4, 168.6 (C-2; *s*-triazine) ppm. HRMS-APCI (rel. int.) *m*/*z*: 666.4500 (100) $[M+H]^+$; $[M+H]^+$ calcd. for $C_{39}H_{61}ClN_5O_2$, 666.4514.

Preparation of compound **3**

At room temperature, into an anhyd. THF (62 mL) solution containing anhyd. piperazine (1.057 g, 12.28 mmol), anhyd. K_2CO_3 (0.424 g, 3.07 mmol) was suspended with vigorous stirring. Compound **2a** (1.700 g, 3.07 mmol) was then added gradually as five equal portions, every 2 h. After each portion (0.340 g), TLC monitoring (eluent EtOH/aq. NH_3 25% 9:0.3) indicated the complete consumption of the starting material **2a** and formation of compound **3** as a major spot. After that, the reaction mixture was stirred at room temperature for additional 24 h, and then evaporated under reduced pressure to dryness. Distilled water (35 mL) was added to the solid residue and the resulted suspension was filtered off and well washed with distilled water for the complete removal of piperazine. After drying, the crude product (1.800 g) was crystallised from boiling ethanol (5 mL) to provide pure compound **3** (1.760 g, 95% yield with respect to **2a**). *2-(Piperazin-1-yl)-4,6-bis[4-(n-octyloxy)phenylamino]-s-triazine* (**3**). White powder; m.p. 137-139 °C (EtOH). Yield 95%. R_f=0.66 (EtOH/aq. NH_3 25% 9:0.3). Elemental analysis calcd (%) for $C_{35}H_{53}N_7O_2$: C 69.62, H 8.85, N 16.24; found: C 69.48, H 9.02, N 16.35. IR (KBr) ν_{max} 3314 (m), 2932 (m), 2853 (m), 1573 (s), 1561 (s), 1519 (s), 1496 (s), 1419 (s), 1307 (m), 1290 (m), 1264 (m), 1048 (w), 1022 (w), 828 (m), 804 (m) cm^{-1}. ^{1}H and 2D-^{1}H,^{1}H-COSY NMR (500 MHz, 5.0 mM in DMSO-d_6, 298 K): δ_H=0.81 (6H, t, $^3J_{H,H}$=6.8 Hz; CH_3), 1.26-1.35 (16H, m; CH_2, *n*-octyl), 1.40 (4H, tt app. qi, $^3J_{H,H}$=7.1 Hz; CH_2, *n*-octyl), 1.68 (4H, tt app. qi, $^3J_{H,H}$=7.3 Hz; OCH_2CH_2, *n*-octyl), 2.70 (4H, t, $^3J_{H,H}$=4.8 Hz; H-3, -5, piperazine), 3.66 (4H, t, $^3J_{H,H}$=4.8 Hz; H-2, -6, piperazine), 3.90 (4H, t, $^3J_{H,H}$=6.5 Hz; OCH_2, *n*-octyl), 6.82 (4H, d, $^3J_{H,H}$=9.0 Hz; H-3, -5, Ph), 7.56 (4H, br s; H-2, -6, Ph), 8.87 (2H, br s; NH) ppm. ^{1}H and 2D-^{1}H,^{1}H-COSY NMR (500 MHz, 5.0 mM in DMSO-d_6, 363 K): δ_H=0.88 (6H, t, $^3J_{H,H}$=7.0 Hz; CH_3), 1.30-1.38 (16H, m; CH_2, *n*-octyl), 1.43 (4H, tt

app. qi, $^3J_{H,H}$=7.4 Hz; CH_2, *n*-octyl), 1.71 (4H, tt app. qi, $^3J_{H,H}$=6.9 Hz; OCH_2CH_2, *n*-octyl), 2.74 (4H, t, $^3J_{H,H}$=5.0 Hz; H-3, -5, piperazine), 3.67 (4H, t, $^3J_{H,H}$=5.0 Hz; H-2, -6, piperazine), 3.94 (4H, t, $^3J_{H,H}$=6.5 Hz; OCH_2, *n*-octyl), 6.82 (4H, d, $^3J_{H,H}$=9.0 Hz; H-3, -5, Ph), 7.55 (4H, d, $^3J_{H,H}$=9.0 Hz; H-2, -6, Ph), 8.50 (2H, s; NH) ppm. 2D-^{1}H-DOSY NMR (500 MHz, 5.0 mM in DMSO-d_6, 298 K): D=191 μm^2 s^{-1}. DEPT ^{13}C NMR (125 MHz, 5.0 mM in DMSO-d_6, 298 K): δ_C=14.4 (CH_3), 22.6 (CH_2CH_3), 26.1, 29.2, 29.3, 31.7 (CH_2; *n*-octyl), 44.6 (C-2, -6; piperazine), 46.0 (C-3, -5; piperazine), 68.0 (OCH_2; *n*-octyl), 114.6 (C-3, -5; Ph), 122.0 (C-2, -6; Ph), 133.7 (C-1; Ph), 154.2 (C-4; Ph), 164.5 (C-2; *s*-triazine), 165.0 (C-4, -6; *s*-triazine) ppm. HRMS-APCI (rel. int.) *m*/*z*: 604.4298 (100) $[M+H]^+$; $[M+H]^+$ calcd. for $C_{35}H_{54}N_7O_2$, 604.4339.

Preparation of G-1 dendron **D-Cl**

Into a cooled (−10 °C) and anhyd. THF (82 mL) solution containing cyanuric chloride (0.305 g, 1.66 mmol), anhyd. K_2CO_3 (0.458 g, 3.32 mmol) was suspended with vigorous stirring. An anhyd. THF (15 mL) solution containing compound **3** (2.000 g, 3.32 mmol) was then added during 2 h. The reaction mixture was stirred at −10 °C for 3 h then let to reach the room temperature where it was kept for additional 36 h. In order to ensure completion of the reaction, the mixture was refluxed for 24 h with TLC monitoring (consumption of the starting material **3**, eluent EtOH/aq. NH_3 25% 9:0.3; formation of compound **D-Cl**, eluent hexane/CH_2Cl_2/acetone 5:3:1). After that, THF was removed by evaporation under reduced pressure to dryness and the resulted solid was taken up with distilled water (50 mL) and then filtered off. After three successive crystallisations from boiling ethanol (15 mL), the crude product (2.150 g) afforded pure compound **D-Cl** (2.080 g, 95% yield with respect to cyanuric chloride).

2-Chloro-4,6-bis{4-{4,6-bis[4-(n-octyloxy)phenylamino]-s-triazin-2-yl}-piperazin-1-yl}-s-triazine **D-Cl**. White powder; m.p. 184-186 °C (EtOH). Yield 95%. R_f =0.50 (hexane/CH_2Cl_2/acetone 5:3:1). Elemental analysis calcd (%) for $C_{73}H_{104}ClN_{17}O_4$: C 66.46, H 7.95, N 18.05; found: C 66.69, H 8.11, N 18.18. IR (KBr) ν_{max} 3408 (w), 3290 (w), 2924 (m), 2853 (m), 1650 (m), 1569 (s), 1515 (s), 1490 (s), 1420 (s), 1302 (m), 1286 (m), 1171 (s), 999 (m), 974 (m), 825 (m), 799 (m) cm^{-1}. ^{1}H and 2D-^{1}H,^{1}H-COSY NMR (500 MHz, 5.0 mM in DMSO-d_6, 298 K): δ_H=0.86 (12H, t, $^3J_{H,H}$=6.8 Hz; CH_3), 1.26-1.31 (32H, m; CH_2, *n*-octyl), 1.41 (8H, tt app. qi, $^3J_{H,H}$=7.0 Hz; CH_2, *n*-octyl), 1.69 (8H, tt app. qi, $^3J_{H,H}$=6.9 Hz; OCH_2CH_2, *n*-octyl), 3.82 (16H, tt app. q, $^3J_{H,H}$=7.5 Hz; piperazine), 3.92 (8H, t, $^3J_{H,H}$=6.5 Hz; OCH_2, *n*-octyl), 6.85 (8H, d, $^3J_{H,H}$=7.5 Hz; H-3, -5, Ph), 7.57 (8H, br s; H-2, -6, Ph), 9.08 (4H, br s; NH) ppm. ^{1}H and 2D-^{1}H,^{1}H-COSY NMR (500 MHz, 5.0 mM in DMSO-d_6, 363 K): δ_H=0.88 (12H, t, $^3J_{H,H}$=7.0 Hz; CH_3), 1.29-1.37 (32H, m; CH_2, *n*-octyl), 1.45 (8H, tt app. qi, $^3J_{H,H}$=7.1 Hz; CH_2, *n*-octyl), 1.72 (8H, tt app. qi, $^3J_{H,H}$=7.0 Hz; OCH_2CH_2, *n*-octyl), 3.84 (16H, s; piperazine), 3.98 (8H, t, $^3J_{H,H}$=6.5 Hz; OCH_2, *n*-octyl), 6.85 (8H, d, $^3J_{H,H}$=9.0 Hz; H-3, -5, Ph), 7.56 (8H, d, $^3J_{H,H}$=9.0 Hz; H-2, -6, Ph), 8.70 (4H, s; NH) ppm. 2D-^{1}H-DOSY NMR (500 MHz, 5.0 mM in DMSO-d_6, 298 K): D=129 μm^2 s^{-1}. DEPT ^{13}C NMR (125 MHz, 5.0 mM in DMSO-d_6, 298 K): δ_C=14.4 (CH_3), 22.6 (CH_2CH_3), 26.1, 29.2, 29.3, 31.7 (CH_2; *n*-octyl), 42.9, 43.0, 43.4 (piperazine), 68.0 (OCH_2; *n*-octyl), 114.7 (C-3, -5; Ph), 122.3 (C-2, -6; Ph), 133.2 (C-1; Ph), 154.5 (C-4; Ph), 164.4 (C-2, T-0; C-4, -6, T-1), 164.8 (C-4, -6; T-0), 169.3 (C-2; T-1) ppm. HRMS-ESI (rel. int.) *m*/*z*: 1318.8237 (10) $[M+H]^+$; $[M+H]^+$ calcd. for $C_{73}H_{105}ClN_{17}O_4$, 1318.8224.

Preparation of G-1 dendron **D-N<P>NH**

Into an anhyd. THF (45 mL) solution containing anhyd. piperazine (0.418 g, 4.84 mmol), anhyd. K_2CO_3 (0.168 g, 1.21 mmol) was suspended with vigorous stirring. The resulted suspension was heated at reflux when compound **D-Cl** (1.600 g, 1.21 mmol) was added gradually as five equal portions, every 8 h. After each portion (0.320 g), TLC monitoring (eluent CH_2Cl_2/acetone/EtOH 8:2:1.5) indicated the presence of the starting material **D-Cl** in small traces only and formation of compound **D-N<P>NH** as a major spot. The

reaction reached completion after additional 15 h of reflux (TLC monitoring). The reaction mixture was then evaporated under reduced pressure to dryness. Distilled water (45 mL) was added to the solid residue and the resulted suspension was filtered off and well washed with distilled water for the complete removal of piperazine. After drying, the crude product (1.630 g) was two times crystallised from boiling ethanol (7 mL) to provide pure compound **D-N<P>NH** (1.540 g, 93% yield with respect to **D-Cl**).

2-(Piperazin-1-yl)-4,6-bis{4-{4,6-bis[4-(n-octyloxy)phenylamino]-s-triazin-2-yl}-piperazin-1-yl}-s-triazine **D-N<P>NH**. White powder; m.p. 161-163 ^{o}C (EtOH). Yield 93%. R_f=0.54 (CH_2Cl_2/acetone/EtOH 8:2:1.5). Elemental analysis calcd (%) for $C_{77}H_{113}N_{19}O_4$: C 67.56, H 8.32, N 19.44; found: C 67.81, H 7.99, N 19.55. IR (KBr) ν_{max} 3413 (m), 3271 (m), 3200 (w), 2924 (m), 2853 (m), 1630 (m), 1557 (s), 1542 (s), 1515 (s), 1418 (s), 1241 (m), 1219 (m), 1000 (m), 826 (m), 802 (m) cm^{-1}. ^{1}H and 2D-^{1}H,^{1}H-COSY NMR (500 MHz, 5.0 mM in DMSO-d_6, 298 K): δ_H=0.86 (12H, t, $^3J_{H,H}$=6.5 Hz; CH_3), 1.26-1.31 (32H, m; CH_2, *n*-octyl), 1.41 (8H, tt app. qi, $^3J_{H,H}$=6.8 Hz; CH_2, *n*-octyl), 1.69 (8H, tt app. qi, $^3J_{H,H}$=6.9 Hz; OCH_2CH_2, *n*-octyl), 3.13 (2H, br s; H-3, -5, P-1), 3.67 (1H, br s; NH, P-1), 3.78 (16H, s; P-0), 3.92 (10H, t, $^3J_{H,H}$=6.3 Hz: 8H, OCH_2, *n*-octyl; 2H, H-2, -6, P-1), 6.84 (8H, d, $^3J_{H,H}$=8.5 H; H-3, -5, Ph), 7.58 (8H, br s; H-2, -6, Ph), 8.69, 8.93 (4H, br s, br s; NH) ppm. ^{1}H and 2D-^{1}H,^{1}H-COSY NMR (500 MHz, 5.0 mM in DMSO-d_6, 363 K): δ_H=0.90 (12H, t, $^3J_{H,H}$=7.0 Hz; CH_3), 1.30-1.37 (32H, m; CH_2, *n*-octyl), 1.44 (8H, tt app. qi, $^3J_{H,H}$=7.1 Hz; CH_2, *n*-octyl), 1.72 (8H, tt app. sx, $^3J_{H,H}$=6.8 Hz; OCH_2CH_2, *n*-octyl), 3.12 (2H, t, $^3J_{H,H}$=5.0 Hz; H-3, -5, P-1), 3.74 (1H, br s; NH, P-1), 3.81 (16H, s; P-0), 3.91 (2H, t, $^3J_{H,H}$=4.9 Hz; H-2, -6, P-1), 3.96 (8H, t, $^3J_{H,H}$=6.5 Hz; OCH_2, *n*-octyl), 6.85 (8H, d, $^3J_{H,H}$=9.0 Hz; H-3, -5, Ph), 7.57 (8H, d, $^3J_{H,H}$=9.0 Hz; H-2, -6, Ph), 8.57 (4H, s; NH) ppm. 2D-^{1}H-DOSY NMR (500 MHz, 5.0 mM in DMSO-d_6, 298 K): D=110 μm^2 s^{-1}. DEPT ^{13}C NMR (125 MHz, 5.0 mM in DMSO-d_6, 298 K): δ_C=14.4 (CH_3), 22.6 (CH_2CH_3), 26.1, 29.2, 29.3, 31.7 (CH_2; *n*-octyl), 43.1, 43.2 (P-0, -1), 68.0 (OCH_2; *n*-octyl), 114.6 (C-3, -5; Ph), 122.1 (C-2, -6; Ph), 133.6 (C-1; Ph), 154.3 (C-4; Ph), 164.5 (C-2; T-1), 165.1 (C-2, T-0; C-4, -6, T-1), 165.20, 165.25 (C-4, -6; T-0) ppm. HRMS-APCI (rel. int.) *m*/*z*: 1368.8956 (30) [M+H]$^+$; [M+H]$^+$ calcd. for $C_{77}H_{114}ClN_{19}O_4$, 1368.9301.

Preparation of G-2 dendrimer **4**

Under inert atmosphere, to an anhyd. 1,4-dioxane (15 mL) solution containing cyanuric chloride (0.018 g, 0.10 mmol), G-1 dendron **D-N<P>NH** (0.493 g, 0.36 mmol) and anhyd. K_2CO_3 (0.050 g, 0.36 mmol) were added with vigorous stirring, at room temperature. The reaction mixture was refluxed for 48 h, i.e., until no more evolution of the reaction was observed (TLC monitoring: for the consumption of the starting material **D-N<P>NH**, eluent acetone/CH_2Cl_2/EtOH 2:8:1.5; formation of product **4**, eluent hexane/acetone 2:1.4). Next, since the HRMS monitoring still indicated an incomplete amination, i.e., the presence of G-2 monomeric and dimeric precursor of **4**, the reaction mixture was evaporated to dryness and 1,4-dioxane was replaced by anhyd. DMF (10 mL). After additional heating at 100 ^{o}C for 48 h of the reaction mixture, this was lyophilised and submitted to column chromatography (eluent hexane/acetone 2:1.4) to provide pure compound **4** (0.260 g, 62% yield with respect to cyanuric chloride).

2,4,6-Tris{4-{4,6-bis{4-{4,6-bis[4-(n-octyloxy)phenylamino]-s-triazin-2-yl}-piperazin-1-yl}-s-triazin-2-yl}-piperazin-1-yl}-s-triazine (**4**). Pale beige powder; m.p. 112-113 ^{o}C. Yield 62%. R_f=0.69 (hexane/acetone 2:1.4). Elemental analysis calcd (%) for $C_{234}H_{336}N_{60}O_{12}$: C 67.21, H 8.10, N 20.10; found: C 66.94, H 7.87, N 20.46. IR (KBr) $\nu_{max.}$ 3433 (w), 3260 (w), 2958 (m), 2925 (m), 2851 (m), 1549 (s), 1535 (s), 1511 (s), 1494 (s), 1472 (s), 1417 (s), 1354 (m), 1297 (w), 1255 (m), 1218 (m), 1172 (w), 997 (m), 826 (m), 803 (m) cm^{-1}. ^{1}H and 2D-^{1}H,^{1}H-COSY NMR (500 MHz, 2.5 mM in DMSO-d_6, 298 K): δ_H=0.85 (36H, m; CH_3), 1.23-1.26 (96H, m; CH_2, *n*-octyl), 1.41 (24H, br s; CH_2, *n*-octyl), 1.70 (24H, br s; OCH_2CH_2, *n*-octyl), 3.80 (72 H, br s; P-0, -1), 3.95 (24H, br s; OCH_2, *n*-octyl), 6.95 (24H, br s; H-3, -5, Ph), 7.49 (24H, br s; H-2, -6, Ph), 9.58, 9.84 (12H, 2×br s; NH) ppm. ^{1}H and 2D-^{1}H,^{1}H-COSY NMR (500 MHz, 2.5 mM in DMSO-d_6,

363 K): δ_H=0.86 (36H, br t, $^3J_{H,H}$=6.3 Hz; CH_3), 1.27-1.33 (96H, m, CH_2; *n*-octyl), 1.43 (24H, tt app. qi, $^3J_{H,H}$=7.0 Hz; CH_2, *n*-octyl), 1.71 (24H, tt ap. qi, $^3J_{H,H}$=7.0 Hz; OCH_2CH_2, *n*-octyl), 3.81 (24H, br s; P-1), 3.84 (48H, br s; P-0), 3.96 (24H, br m; OCH_2, *n*-octyl), 6.88 (24H, d, $^3J_{H,H}$=8.5 Hz; H-3, -5, Ph), 7.53 (24H, d, $^3J_{H,H}$=9.0 Hz; H-2, -6, Ph), 8.93 (12H, br s; NH) ppm. 2D-^{1}H-DOSY NMR (500 MHz, 2.5 mM in DMSO-d_6, 298 K): D=89 μm^2 s^{-1}. ^{13}QC NMR (125 MHz, 2.5 mM in DMSO-d_6, 363 K): δ_C=13.7 (12C; CH_3), 21.9 (12C; CH_2CH_3), 25.5 (12C; CH_2, *n*-octyl), 28.5, 28.7, 28.8 (36C; CH_2, *n*-octyl), 31.1 (12C; OCH_2CH_2, *n*-octyl), 42.5, 42.6, 42.8, 42.9, 43.1, 43.3 (36C; P-0, -1), 68.1 (12C; OCH_2, *n*-octyl), 114.8 (24C; C-3, -5, Ph), 123.0 (24C; C-2, -6, Ph), 131.7 (12C; C-1, Ph), 155.1 (12C; C-4, Ph), 165.1 (30C; T-0, -1, -2) ppm. HRMS (ESI+, ACN+TFA) (rel. int.) *m*/z: 2091.3960 (1000) [0.5(*M*+4H)]$^+$. Anal. calcd. for [0.5(*M*+4H)]$^+$ 2091.3919.

Preparation of G-2 dendrimer **5**

Under inert atmosphere and with vigorous stirring, to an anhyd. DMF (4.5 mL) solution containing G-0 dendron **D-Cl** (0.160 g, 0.12 mmol) and 1,3,5-tris(piperazinomethyl)benzene **A** (0.015 g, 0.040 mmol), anhyd. K_2CO_3 (0.017 g, 0.12 mmol) was added at room temperature. The resulted suspension was heated at 90–95 °C for 96 h (TLC monitoring: consumption of **D-Cl**, eluent hexane/CH_2Cl_2/acetone 5:3:1; consumption of **A** and formation of product **5**, eluent hexane/CH_2Cl_2/acetone 1:4:0.5). DMF was distilled under reduced pressure and the solid residue was taken up with distilled water (12 mL) then filtered off. The dried crude product (0.155 g) was purified by column chromatography (eluent hexane/CH_2Cl_2/acetone 1:4:0.5) to provide pure product **5** (0.110 g, 65% yield).

1,3,5-Tris{{4-{4,6-bis{4-{4,6-bis[4-(n-octyloxy)phenylamino]-s-triazin-2-yl}-piperazin-1-yl}-s-triazin-2-yl}-piperazin-1-yl}methyl}benzene (**5**). Beige powder; m.p. 210-211 °C. Yield 65%. R_f=0.92 (hexane/CH_2Cl_2/acetone 1:4:0.5). Elemental analysis calcd (%) for $C_{240}H_{345}N_{57}O_{12}$: C 68.30, H 8.24, 18.92; found: C 67.98, H 8.57, N 18.86. IR (KBr) $\nu_{max.}$ 3412 (w), 2926 (m), 2853 (m), 1600 (m), 1547 (s), 1512 (s), 1493 (s), 1476 (s), 1419 (s), 1260 (m), 1242 (m), 1219 (m), 1001 (m), 827 (w), 804 (w) cm^{-1}. ^{1}H and 2D-^{1}H,^{1}H-COSY NMR (500 MHz, 5.0 mM in DMSO-d_6, 298 K): δ_H=0.84, 0.86 (36H, 2×t, $^3J_{H,H}$=4.6 Hz, $^3J_{H,H}$=5.3 Hz; CH_3), 1.25-1.28 (96H, br m; CH_2, *n*-octyl), 1.41 (24H, tt app. h, $^3J_{H,H}$=7.3 H; CH_2, *n*-octyl), 1.69 (24H, tt app. sp, $^3J_{H,H}$=7.1 Hz; OCH_2CH_2, *n*-octyl), 3.78 (72 H, br s; P-0, -1; 6H, CH_2-*inner*), 3.92, 3.91 (24H, 2×t, $^3J_{H,H}$=6.8 Hz, $^3J_{H,H}$=6.5 Hz; OCH_2, *n*-octyl), 6.83-6.85 (24H, br s; H-3, -5, Ph-*peripheral*), 7.57 (27H: 24H, br s; H-2, -6, Ph-*peripheral*; 3H, benzene-*core*), 8.91, 8.93 (12H, 2×br s; NH) ppm. ^{1}H and 2D-^{1}H,^{1}H-COSY NMR (500 MHz, 5.0 mM in DMSO-d_6, 363 K): δ_H=0.87 (36H, t, $^3J_{H,H}$=6.5 Hz; CH_3), 1.29-1.35 (96H, m; CH_2, *n*-octyl), 1.43 (24H, tt app. qi, $^3J_{H,H}$=7.0 Hz; CH_2, *n*-octyl), 1.71 (24H, tt app. sp, $^3J_{H,H}$=7.4 Hz; OCH_2CH_2, *n*-octyl), 3.79, 3.80 (78H: 72H, br s; P-0, P-1; 6H, br s; CH_2-*inner*), 3.94, 3.96 (24H, 2×t, $^3J_{H,H}$=6.3 Hz, $^3J_{H,H}$=6.0 Hz; OCH_2, *n*-octyl), 6.82, 6.85 (24H, d, dd, $^3J_{H,H}$=8.5 Hz, $^3J_{H,H}$=6.5 Hz, $^4J_{H,H}$=2.5 Hz; H-3, -5, Ph-*peripheral*), 7.55, 7.57 (27H: 24H, 2×dd, $^3J_{H,H}$=7.5 Hz, $^4J_{H,H}$=1.8 Hz, $^3J_{H,H}$=8.0 Hz, $^4J_{H,H}$=3.0 Hz; H-2, -6, Ph-*peripheral*; 3H, benzene-*core*), 8.51, 8.53, 8.55, 8.60 (12H, br s; NH) ppm. 2D-^{1}H-DOSY NMR (500 MHz, 5.0 mM in DMSO-d_6, 298 K): D=88 μm^2 s^{-1}. 2D-^{1}H,^{13}C-HSQC, DEPT and ^{13}QC NMR (125 MHz, 5.0 mM in DMSO-d_6, 298 K): δ_C=14.4 (12C; CH_3), 22.9 (12C; CH_2CH_3), 26.1 (12C; CH_2, *n*-octyl), 29.2, 29.3 (36C; CH_2, *n*-octyl), 31.7 (12C; OCH_2CH_2, *n*-octyl), 43.0, 43.2 (39C: 36C, P-0, -1; 3C, CH_2-*inner*), 68.0 (12C; OCH_2, *n*-octyl), 114.6 (24C; C-3, -5, Ph-*peripheral*), 122.1 (27C: 24C, C-2, -6, Ph-*peripheral*; 3C, C-2, -4, -6, benzene-*core*), 133.6 (15C: 12C, C-1, Ph-*peripheral*; 3C, C-1, -3, -5, benzene-*core*), 154.3 (12C; C-4, Ph-*peripheral*), 164.5 (12C; C-4, -6, T-0), 165.06, 165.10, 165.18, 165.22, 165.3 (6C, C-2, T-0; 9C, T-1) ppm. HRMS (ESI+, ACN+TFA) (rel. int.) *m*/z: 2110.9146 (32) [0.5(*M*+4H)]$^+$. Anal. calcd. for [0.5(*M*+4H)]$^+$ 2110.9225.

Preparation of G -2 dendrimer **6**

Under inert atmosphere and vigorous stirring, into an anhyd. DMF (8 mL) solution containing G-1 dendron **D-Cl** (0.680 g, 0.516 mmol) and 2,4,6-tris(4-hydroxyphenylamino)-*s*-triazine **B** (65.8 mg, 0.16 mmol), anhyd. K_2CO_3 (0.265 g, 1.92 mmol) was suspended. The reaction mixture was heated at 100 ^oC for 56 h until no progress of the reaction was observed (TLC monitoring: consumption of **D-Cl**, eluent hexane/CH_2Cl_2/acetone 5:3:1; consumption of **B**, eluent ligroin/acetone 1:1; formation of **6**, eluent $CHCl_3$/hexane/acetone 4:1:0.3). In order to remove the unreacted **D-Cl**, the reaction mixture was evaporated to dryness under reduced pressure and the solid residue was dissolved in THF (25 mL) to which a solution obtained by dissolving KOH (0.210 g) in distilled water (20 mL) was added. Under inert atmosphere, the alkaline solution (pH ~ 13) was stirred for 24 h at room temperature then THF was removed under reduced presure. The resulted suspension was filtered off and well washed with hot distilled water to neutrality. The dried crude product was purified by column chromatography (eluent $CHCl_3$/hexane/acetone 4:1:0.3) to provide pure compound **6** [0.240 g, 35% yield with respect to 2,4,6-tris(4-hydroxyphenylamino)-*s*-triazine **B**].

2,4,6-Tris{4-{4,6-bis{4-{4,6-bis[4-(n-octyloxy)phenylamino]-s-triazin-2-yl}-piperazin-1-yl}-s-triazin-2-oxy}-phenylamino}-s-triazine (**6**). Beige powder; m.p. 110-111 ^oC (column chromatography, eluent $CHCl_3$/hexane/acetone 4:1:0.3); R_f=0.64. Yield 35%. Elemental analysis calcd (%) for $C_{240}H_{327}N_{57}O_{15}$: C 67.82, H 7.75, N 18.78; found: C 67.93, H 7.65, N 18.63. IR (KBr) $\nu_{max.}$ 3411 (w), 2959 (m), 2924 (m), 2852 (m), 1736 (w), 1651 (w), 1577 (s), 1559 (s), 1515 (s), 1491 (s), 1438 (m), 1419 (m),1380 (w), 1358 (m), 1287 (w), 1260 (w), 1243 (m), 1204 (w), 1110 (w), 1003 (w), 973 (m), 836 (w), 802 (w) cm^{-1}. ^1H and 2D-^1H,^1H-COSY NMR (500 MHz, 5.0 mM in DMSO-d_6, 363 K): δ_H=0.87 (36H, t, $^3J_{H,H}$=6.5 Hz; CH_3), 1.27-1.32 (96H, m; CH_2, *n*-octyl), 1.41 (24H, tt ap. qi, $^3J_{H,H}$=7.1 Hz; CH_2, *n*-octyl), 1.69 (24H, tt ap. qi, $^3J_{H,H}$=6.9 Hz; OCH_2CH_2, *n*-octyl), 3.77 (48H, br s; piperazine), 3.92 (24H, t, $^3J_{H,H}$=6.3 Hz; OCH_2, *n*-octyl), 6.80 (24H, d, $^3J_{H,H}$=9.0 Hz; H-3, -5, Ph-*peripheral*), 7.13 (6H, d, $^3J_{H,H}$=9.0 Hz; H-3, -5, Ph-*linker*), 7.53 (24H, d, $^3J_{H,H}$=9.0 Hz; H-4, -6, Ph-*peripheral*), 7.84 (6H, d, $^3J_{H,H}$=9 Hz; H-4, -6, Ph-*linker*), 8.55 (12H, s; NH-*peripheral*), 9.02 (3H, br s; NH-*core*) ppm. DEPT and ^{13}QC NMR (125 MHz, 2.5 mM in DMSO-d_6, 363 K): δ_C=13.7 (12C; CH_3), 21.9 (12C; CH_2CH_3), 25.5 (12C; CH_2, *n*-octyl), 28.5 (12C; CH_2, *n*-octyl), 28.7 (12C; CH_2, *n*-octyl), 28.9 (12C; CH_2, *n*-octyl), 31.2 (12C; OCH_2CH_2, *n*-octyl), 42.6, 42.9 (24C; piperazine), 68.1 (12C; OCH_2, *n*-octyl), 114.5 (24C; C-3, -5, Ph-*peripheral*), 121.15 (6C; C-3, -5, Ph-*linker*), 121.22 (6C; C-2, -6, Ph-*linker*) 122.1 (24C; C-2, -6, Ph-*peripheral*), 133.1 (12C; C-1, Ph-*peripheral*), 136.8 (3C; C-1, Ph-*linker*), 147.4 (3C; C-4, Ph-*linker*), 154.3 (12C; C-4, Ph-*peripheral*), 164.2 (12C; C-4, -6, T-0), 164.4 (3C; T-2), 164.9 (6C; C-2, T-0), 166.1 (6C; C-4, -6, T-1), 170.6 (3C; C-2, T-1) ppm. HRMS (ESI+, ACN+TFA) (rel. int.) *m*/z: 2125.8369 (100) $[0.5(M+4H)]^+$. Anal. calcd. for $[0.5(M+4H)]^+$ 2125.8445.

Preparation of G-2 dendrimer **7a**

Under inert atmosphere, into an anhyd. THF (14 mL) solution containing G-1 dendron **D-N<P>NH** (0.4000 g, 0.29 mmol), anhyd. K_2CO_3 (0.0400 g, 0.29 mmol) was suspended with vigorous stirring. The suspension was cooled at 0 °C when trimesic acid trichloride (0.0254 g (0.095 mmol) as anhyd. THF (1.5 mL) solution was injected dropwise. The reaction mixture was stirred at room temperature for 72 h then heated at reflux (48 h) until no more evolution was observed (TLC monitoring, eluent acetone/CH_2Cl_2/EtOH 2:8:0.5). The reaction mixture was then evaporated under reduced pressure to dryness. Distilled water (10 mL) was added to the solid residue and the suspension was filtered off and well washed with distilled water. The crude and dried product was dissolved in $CHCl_3$ (25 mL) and the solution was washed four times with 1% NaOH aq. soln. (25 mL), then with distilled water (25 mL) to neutrality. The organic layer was dried over anhyd. Na_2SO_4, filtered off and evaporated under reduced pressure. The solid residue was purified by column chromatography (eluent acetone/CH_2Cl_2/EtOH 2:8:0.5) to provide pure compound **7a** (0.2000 g, 50% yield

with respect to trimesic acid trichloride).

1,3,5-Tris{4-{4,6-bis{4-{4,6-bis[4-(n-octyloxy)phenylamino]-s-triazin-2-yl}-piperazin-1-yl}-s-triazin-2-yl}-piperazin-1-ylcarbonyl}benzene **7a**. White-yellowish powder; m.p. 140-141 °C (column chromatography, eluent acetone/CH_2Cl_2/EtOH 2:8:0.5); R_f=0.95. Yield 50%. Elemental analysis calcd (%) for $C_{240}H_{339}N_{57}O_{15}$: C 67.62, H 8.02, N 18.73; found: C 67.31, H 7.85, N 19.02. IR (KBr) ν_{max} 2958 (m), 2925 (m), 2852 (m), 1633 (m), 1582 (m), 1543 (s), 1512 (s), 1493 (s), 1474 (s), 1418 (s), 1261 (s), 1242 (s), 1220 (s), 998 (m), 826 (m), 805 (m) cm^{-1}. ^{1}H and 2D-^{1}H,^{1}H-COSY NMR (500 MHz, 2.5 mM in DMSO-d_6, 298 K): δ_H=0.85 (36H, br s; CH_3), 1.27 (96H, br s; CH_2, *n*-octyl), 1.40 (24H, br s; CH_2, *n*-octyl), 1.69 (24H, tt app. q, $^3J_{H,H}$=7.0 Hz; OCH_2CH_2, *n*-octyl), 3.78 (72H, br s; P-0, -1), 3.92 (24H, t, $^3J_{H,H}$=6.3 Hz; OCH_2, *n*-octyl), 6.84 (24H, br s; H-3, -5, Ph-*peripheral*), 7.58 (27H, br s: 24H, H-2, -6, Ph-*peripheral*; 3H, benzene-*core*), 8.94 (12H, br s, br s; NH) ppm. ^{1}H and 2D-^{1}H,^{1}H-COSY NMR (500 MHz, 2.5 mM in DMSO-d_6, 363 K): δ_H=0.87 (36H, br s; CH_3), 1.28 (96H, br s; CH_2, *n*-octyl), 1.42 (24H, br s; CH_2, *n*-octyl), 1.70 (24H, br s; OCH_2CH_2, *n*-octyl), 3.60 (12H, br s; H-2, -6, P-1), 3.80 (60H: 48H, br s, P-0; 12H, H-3, -5, P-1), 3.94 (24H, t, $^3J_{H,H}$=5.3 Hz; OCH_2, *n*-octyl), 6.83 (24H, d, $^3J_{H,H}$=7.0 Hz; H-3, -5, Ph-*peripheral*), 7.55 (24H, d, $^3J_{H,H}$=6.0 Hz; H-2, -6, Ph-*peripheral*), 7.60 (3H, s; benzene-*core*), 8.55 (12H, s; NH) ppm. 2D-^{1}H-DOSY NMR (500 MHz, 2.5 mM in DMSO-d_6, 298 K): D=107 μm^2 s^{-1}. DEPT and ^{13}QC NMR (125 MHz, 5.0 mM in DMSO-d_6, 298 K): δ_C=14.4 (12C; CH_3), 22.6 (12C; CH_2CH_3), 26.1 (12C; CH_2, *n*-octyl), 29.2, 29.3 (36C; CH_2, *n*-octyl), 31.7 (12C; OCH_2CH_2, *n*-octyl), 43.06, 43.14, 43.20, 43.24 (36C; P-0, -1), 68.0 (12C; OCH_2, *n*-octyl), 114.7 (24C; C-3, -5, Ph-*peripheral*), 122.1, 122.2 (27C: 24C, C-2, -6, Ph-*peripheral*; 3C, C-2, -4, -6, benzene-*core*), 133.5 (15C: 12C, C-1, Ph-*peripheral*; 3C, C-1, -3, -5, benzene-*core*), 154.3 (12C; C-4, Ph-*peripheral*), 164.36,164.39, 164.41, 164.46, 164.51 (12C; C-4, -6, T-0), 165.1 (6C: 3C, C=O; 3C, C-2, T-1), 165.3 (12C: 6C, C-2, T-0; 6C, C-4, -6, T-1) ppm. HRMS (ESI+, ACN+TFA) *m/z*: 2131.9060 (63) $[0.5(M+4H)]^+$, 1421.6078 (100) $[0.33(M+5H)]^+$; calcd. for $[0.5(M+4H)]^+$ 2131.8915; calcd. for $[0.33(M+5H)]^+$ 1421.5969.

Preparation of G-2 dendrimer **7b**

At room temperature and with vigorous stirring, compound **D-N<P>NH** (0.0860 g, 0.0628 mmol) and trimesic acid (0.0044 g, 0.0209 mmol) were dissolved in anhyd. THF (2 mL) and heated at 60 °C for 15 min. The resulted clear solution was evaporated under reduced presure to dryness and the solid residue was dried under vacuum for additional 48 h to provide compound **7b** with quantitative yield (0.0904 g).

Tris{4-{4,6-bis{4-{4,6-bis[4-(n-octyloxyphenyl)amino]-s-triazin-2-yl}-piperazin-1-yl}-s-triazin-2-yl}-piperazin-1-ium}-1,3,5-benzene tricarboxylate **7b**. White powder; m.p. 140-141 °C. Yield 100%. Elemental analysis calcd (%) for $C_{240}H_{345}N_{57}O_{18}$: C 66.78, H 8.06, N 18.50; found: C 66.55, H 8.43, N 18.24. IR (KBr) $\nu_{max.}$ 3413 (w), 3273 (w), 2957 (m), 2926 (m), 2853 (m), 1702 (w), 1556 (s), 1542 (s), 1513 (s), 1475 (s), 1438 (m), 1419 (s), 1354 (w), 1258 (m), 1220 (m), 1172 (m), 999 (m), 826 (w) cm^{-1}. ^{1}H and 2D-^{1}H,^{1}H-COSY NMR (500 MHz, 2.5 mM in DMSO-d_6, 298 K): δ_H=0.86 (36H, t, $^3J_{H,H}$=6.8 Hz; CH_3), 1.26-1.31 (96H, m; CH_2, *n*-octyl), 1.41 (24H, tt ap. qi, $^3J_{H,H}$=7.0 Hz; CH_2, *n*-octyl), 1.69 (24H, tt ap. qi, $^3J_{H,H}$=6.9 Hz; OCH_2CH_2, *n*-octyl), 3.09 (12H, br s; H-2, -6, P-1), 3.78 (60H, br s: 48H, P-0; 12H, H-3, -5, P-1), 3.92 (24H, t, $^3J_{H,H}$=6.5 Hz; OCH_2, *n*-octyl), 6.84 (24H, d, $^3J_{H,H}$=8.5 Hz; H-3, -5, Ph-*peripheral*), 7.58 (24H, br s; H-2, -6, Ph-*peripheral*), 8.63 (3H, s; benzene-*core*), 8.93 (12H, br s; NH) ppm. ^{1}H and 2D-^{1}H,^{1}H-COSY NMR (500 MHz, 2.5 mM in DMSO-d_6, 363 K): δ_H=0.88 (36H, t, $^3J_{H,H}$=6.8 Hz; CH_3), 1.30-1.39 (96H, m; CH_2, *n*-octyl), 1.44 (24H, tt ap. qi, $^3J_{H,H}$=7.0 Hz; CH_2, *n*-octyl), 1.72 (24H, tt ap. qi, $^3J_{H,H}$=6.9 Hz; OCH_2CH_2, *n*-octyl), 2.90 (12H, br t, $^3J_{H,H}$=5.3 Hz; H-2, -6, P-1), 3.80 (60H, br s: 48H, P-0; 12H, H-3, -5, P-1), 3.96 (24H, t, $^3J_{H,H}$=6.5 Hz; OCH_2, *n*-octyl), 6.85 (24H, d, $^3J_{H,H}$=9.0 Hz; H-3, -5, Ph-*peripheral*), 7.57 (24H, d, $^3J_{H,H}$=9.0 Hz; H-2, -6, Ph-*peripheral*), 8.53 (12H, s; NH) 8.64 (3H, s; benzene-*core*) ppm. 2D-^{1}H-DOSY NMR (500 MHz, 2.5 mM in DMSO-d_6, 298 K): D=105 and 225 μm^2 s^{-1}. ^{13}QC, DEPT and 2D-^{1}H,^{13}C-HSQC, -HMBC NMR (125 MHz,

2.5 mM in DMSO-d_6, 298 K): δ_C=13.7 (12C; CH_3), 21.9 (12C; CH_2CH_3), 25.5 (12C; CH_2, *n*-octyl), 28.5, 28.7, 28.9 (36C; CH_2, *n*-octyl), 31.1 (12C; OCH_2CH_2, *n*-octyl), 42.7, 42.8 (36C; P-0, -1), 68.1 (12C; OCH_2, *n*-octyl), 114.6 (24C; C-3, -5, Ph-*peripheral*), 122.1 (27C: 24C, C-2, -6, Ph-*peripheral*; 3C, C-2, -4, -6, benzene-*core*), 132.31, 132.34, 132.37 (3C; C-1, -3, -5, benzene-*core*), 133.3, 133.4 (12C; C-1, Ph-*peripheral*), 154.2 (12C; C-4, Ph-*peripheral*), 164.4 (12C; C-4, -6, T-0), 165.0, 165.1, 165.2 (15C: 6C, C-2, T-0; 6C, C-4, -6, T-1; 3C, C-2, T-1), 165.9 (3C, C=O) ppm.

Preparation of G-2 dendrimer **8**

An anhyd. DMSO (2 mL) solution containing compounds **D-N<P>NH** (0.0812 g, 0.0593 mmol) and **C1** (0.0114 g, 0.0198 mmol) was heated at 95 °C for 15 min. After cooling to room temperature, the solution was lyophilised to provide compound **8** in quantitative yield (0.0926 g).

Tris{4-{4,6-bis{4-{4,6-bis[4-(n-octyloxy)phenylamino]-s-triazin-2-yl}-piperazin-1-yl}-s-triazin-2-yl}-piperazin-1-ium} 2,4,6-tris[4-(methoxyl)phenylamino]-s-triazine tricarboxylate **8**. White powder; m.p. 169-170 ^oC. Elemental analysis calcd (%) for $C_{258}H_{363}N_{63}O_{21}$: C 66.17, H 7.81, N 18.84; found: C 66.03, H 7.94, N 19.07. IR (KBr) $\nu_{max.}$ 3412 (w), 3260 (w), 2957 (w), 2925 (m), 2852 (w), 1651 (w), 1555 (s), 1541 (s), 1512 (s), 1491 (s), 1475 (s), 1418 (s), 1357 (w), 1256 (m), 1244 (m), 1218 (m), 1171 (w), 1000 (m), 827 (w), 803 (w), 633 (w) cm^{-1}. ^1H and 2D-^1H,^1H-COSY NMR (500 MHz, 5.0 mM in DMSO-d_6, 298 K): δ_H=0.86 (36H, t, $^3J_{H,H}$=6.8 Hz; CH_3), 1.27-1.34 (96H, m; CH_2, *n*-octyl), 1.41 (24H, tt ap. qi, $^3J_{H,H}$=7.1 Hz; CH_2, *n*-octyl), 1.69 (24H, tt ap. qi, $^3J_{H,H}$=6.9 Hz; OCH_2CH_2, *n*-octyl), 2.97 (12H, br s; H-2, -6, P-1), 3.78, 3.82 (60H, 2×br s: 48H, P-0; 12H, H-3, -5, P-1), 3.92 (24H, t, $^3J_{H,H}$=6.3 Hz; OCH_2, *n*-octyl), 6.73 (6H, br s; H-3, -5, Ph-*inner*), 6.85 (24H, d, $^3J_{H,H}$=8.0 Hz; H-3, -5, Ph-*peripheral*), 7.43 (6H, br s; H-2, -6, Ph-*inner*), 7.59 (24H, br s; H-2, -6, Ph-*peripheral*), 8.93 (15H, br s: 12H, NH-*peripheral*; 3H, NH-*inner*) ppm. ^1H, 2D-^1H,^1H-COSY NMR (500 MHz, 5.0 mM in DMSO-d_6, 363 K): δ_H=0.86 (36H, t, $^3J_{H,H}$=6.8 Hz; CH_3), 1.30-1.37 (96H, m; CH_2, *n*-octyl), 1.44 (24H, tt ap. qi, $^3J_{H,H}$=7.0 Hz; CH_2, *n*-octyl), 1.72 (24H, tt ap. qi, $^3J_{H,H}$=6.9 Hz; OCH_2CH_2, *n*-octyl), 2.92 (12H, br t, $^3J_{H,H}$=4.5 Hz; H-2, -6, P-1), 3.80 (66H, br s: 48H, P-0; 12H, H-3, -5, P-1; 6H, CH_{2-}*inner*), 3.96 (24H, t, $^3J_{H,H}$=6.3 Hz; OCH_2, *n*-octyl), 6.83 (6H, d, $^3J_{H,H}$=9.0 Hz; H-3, -5, Ph-*inner*), 6.85 (24H, d, $^3J_{H,H}$=8.5 Hz; H-3, -5, Ph-*peripheral*), 7.57 (30H, d, $^3J_{H,H}$=9.0 Hz: 24H, H-2, -6, Ph-*peripheral*; 6H, H-2, -6, Ph-*inner*), 8.54 (12H, s; NH-*peripheral*), 8.60 (3H, br s; NH-*inner*) ppm. 2D-^1H-DOSY NMR (500 MHz, 5.0 mM in DMSO-d_6, 298 K): D=113 $\mu m^2\ s^{-1}$. ^{13}QC, DEPT and 2D-^1H,^{13}C-HSQC, -HMBC NMR (125 MHz, 5.0 mM in DMSO-d_6, 298 K): δ_C=14.4 (12C; CH_3), 22.6 (12C; CH_2CH_3), 26.1 (12C; CH_2, *n*-octyl), 29.2, 29.3 (36C; CH_2, *n*-octyl), 31.7 (12C; OCH_2CH_2, *n*-octyl), 43.1, 43.2 (30C; P-0, -1), 68.0 (12C; OCH_2, *n*-octyl), 114.6 (30C: 24C, C-3, -5, Ph-*peripheral*; 6C, C-3, -5, Ph-*inner*), 122.1 (30C: 24C, C-2, -6, Ph-*peripheral*; 6C, C-2, -6, Ph-*inner*), 133.6 (15C: 12C, C-1, Ph-*peripheral*; 3C, C-1, Ph-*inner*), 154.3 (15C: 12C, C-4, Ph-*peripheral*; 3C, C-4, Ph-*inner*), 164.5 (15C: 6C, C-2, T-0; 9C, T-1), 165.0-165.3 (15C: 12C, C-4, -6, T-0; 3C, T-2) ppm.

Preparation of G-2 dendrimer **9**

An anhyd. DMSO (2 mL) solution containing compounds **D-N<P>NH** (0.0802 g, 0.0586 mmol) and **C3** (0.0129 g, 0.0195 mmol) was heated at 95 °C for 15 min. After cooling to room temperature, the solution was lyophilised to provide compound **9** with quantitative yield (0.0931 g).

Tris{4-{4,6-bis{4-{4,6-bis[4-(n-octyloxy)phenylamino]-s-triazin-2-yl}-piperazin-1-yl}-s-triazin-2-yl}-piperazin-1-ium} 2,4,6-tris[4-(prop-1-yloxy)]phenylamino-s-triazine tricarboxylate **9**. White powder, m.p. 169-170 ^oC. Yield 100%. Elemental analysis calcd (%) for $C_{264}H_{375}N_{63}O_{21}$: C 66.51, H 7.93, N, 18.51; found: C 66.82, H 7.64, N 18.21. IR (KBr) $\nu_{max.}$ 3412 (w), 3274 (w), 2957 (m), 2926 (w), 2852 (m), 1542 (s), 1512 (s), 1492 (s), 1475 (s), 1418 (s), 1356 (w), 1243 (m), 1218 (m), 1171 (w), 1001 (m), 827 (w), 803 (w), 593

(w) cm^{-1}. ^1H and 2D-^1H,^1H-COSY NMR (500 MHz, 5.0 mM in DMSO-d_6, 298 K): δ_H=0.86 (36H, t, $^3J_{H,H}$=6.5 Hz; CH_3), 1.27-1.34 (96H, m; CH_2, *n*-octyl), 1.41 (24H, tt ap. qi, $^3J_{H,H}$=7.0 Hz; CH_2, *n*-octyl), 1.69 (24H, tt ap. qi, $^3J_{H,H}$=6.9 Hz; OCH_2CH_2, *n*-octyl), 1.94 (6H, tt ap. q, $^3J_{H,H}$=6.5 Hz; β-CH_2-*inner*), 2.39 (6H, t, $^3J_{H,H}$=7.3 Hz; α-CH_2-*inner*), 3.15 (12H, br s; H-2, -6, P-1), 3.78 (48H, s; P-0), 3.92 (36H, t, $^3J_{H,H}$=6.5 Hz: 24H, OCH_2, *n*-octyl; 12H, H-3, -5, P-1), 3.93 (6H, t, $^3J_{H,H}$=6.0 Hz; γ-CH_2-*inner*), 6.85 (30H, d, $^3J_{H,H}$=9.0 Hz: 24H, H-3, -5, Ph-*peripheral*; 6H, H-3, -5, Ph-*inner*), 7.58 (30H: 24H, br s, H-2, -6, Ph-*peripheral*; 6H, H-2, -6, Ph-*inner*), 8.70 (3H, br s; NH-*inner*), 8.94 (12H, br s; NH-*peripheral*) ppm. ^1H and 2D-^1H,^1H-COSY NMR (500 MHz, 5.0 mM in DMSO-d_6, 363 K): δ_H=0.86 (36H, t, $^3J_{H,H}$=6.8 Hz; CH_3), 1.29-1.39 (96H, m; CH_2, *n*-octyl), 1.44 (24H, tt ap. qi, $^3J_{H,H}$=7.0 Hz; CH_2, *n*-octyl), 1.72 (24H, tt ap. qi, $^3J_{H,H}$=6.9 Hz; OCH_2CH_2, *n*-octyl), 1.97 (6H, tt app. qi., $^3J_{H,H}$=7.0 Hz; β-CH_2-*inner*), 2.39 (6H, t, $^3J_{H,H}$=7.3 Hz; α-CH_2-*inner*), 3.17 (12H, br s; H-2, -6, P-1), 3.81 (48H, s; P-0), 3.96 (36H, t, $^3J_{H,H}$=6.5 Hz: 24H, OCH_2, *n*-octyl; 12H, H-3, -5, P-1), 4.00 (6H, t, $^3J_{H,H}$=6.5 Hz; γ-CH_2-*inner*), 6.85 (30H, d, $^3J_{H,H}$=8.5 Hz: 24H, H-3, -5, Ph-*peripheral*; 6H, H-3, -5, Ph-*inner*), 7.56 (24H, d, $^3J_{H,H}$=9.0 Hz; H-2, -6, Ph-*peripheral*), 7.59 (6H, d, $^3J_{H,H}$=8.0 Hz; H-2, -6, Ph-*inner*), 8.54 (12H, s; NH-*peripheral*), 8.57 (3H, br s; NH-*inner*) ppm. 2D-^1H-DOSY NMR (500 MHz, 5.0 mM in DMSO-d_6, 298 K): D= 115 μm^2 s^{-1}. ^{13}QC, DEPT and 2D-^1H,^{13}C-HSQC, -HMBC NMR (125 MHz, 5.0 mM in DMSO-d_6, 298 K): δ_C=14.4 (12C; CH_3), 22.6 (12C; CH_2CH_3, *n*-octyl), 24.8 (3C; β-CH_2-*inner*), 26.1 (12C; CH_2, *n*-octyl), 29.2, 29.3 (36C; CH_2, *n*-octyl), 30.6 (3C; α-CH_2-*inner*), 31.7 (12C; OCH_2CH_2, *n*-octyl), 43.1, 43.2 (30C: 24C, P-0; 6C, C-3, -5, P-1), 67.2 (3C; γ-CH_2-*inner*), 68.0 (12C; OCH_2, *n*-octyl), 114.7 (30C: 24C, C-3, -5, Ph-*peripheral*; 6C, C-3, -5, Ph-*core*), 122.1 (24C; C-2, -6, Ph-*peripheral*), 122.5 (6C; C-2, -6, Ph-*inner*), 133.5 (15C: 12C, C-1, Ph-*peripheral*; 3C, C-1, Ph-*inner*), 154.3 (15C: 12C, C-4, Ph-*peripheral*; 3C, C-4, Ph-*inner*), 164.4 (15C: 6C, C-2, T-0; 9C, T-1), 165.1, (12C; C-4, -6, T-0), 165.3 (3C; T-2),174.6 (3C; COO) ppm.

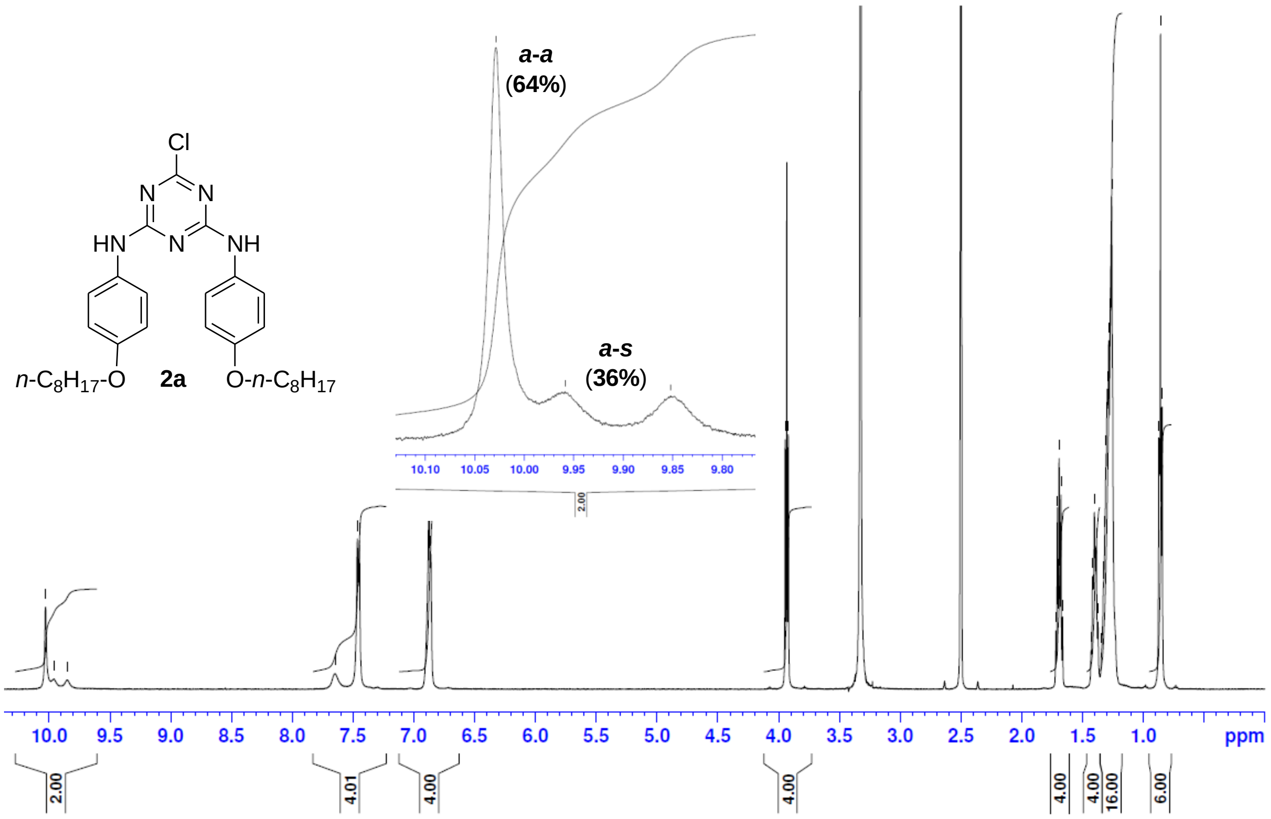

Figure S1: ^{1}H NMR spectrum of compound **2a** (500 MHz, 5.0 mM in DMSO-d_6, 298 K).

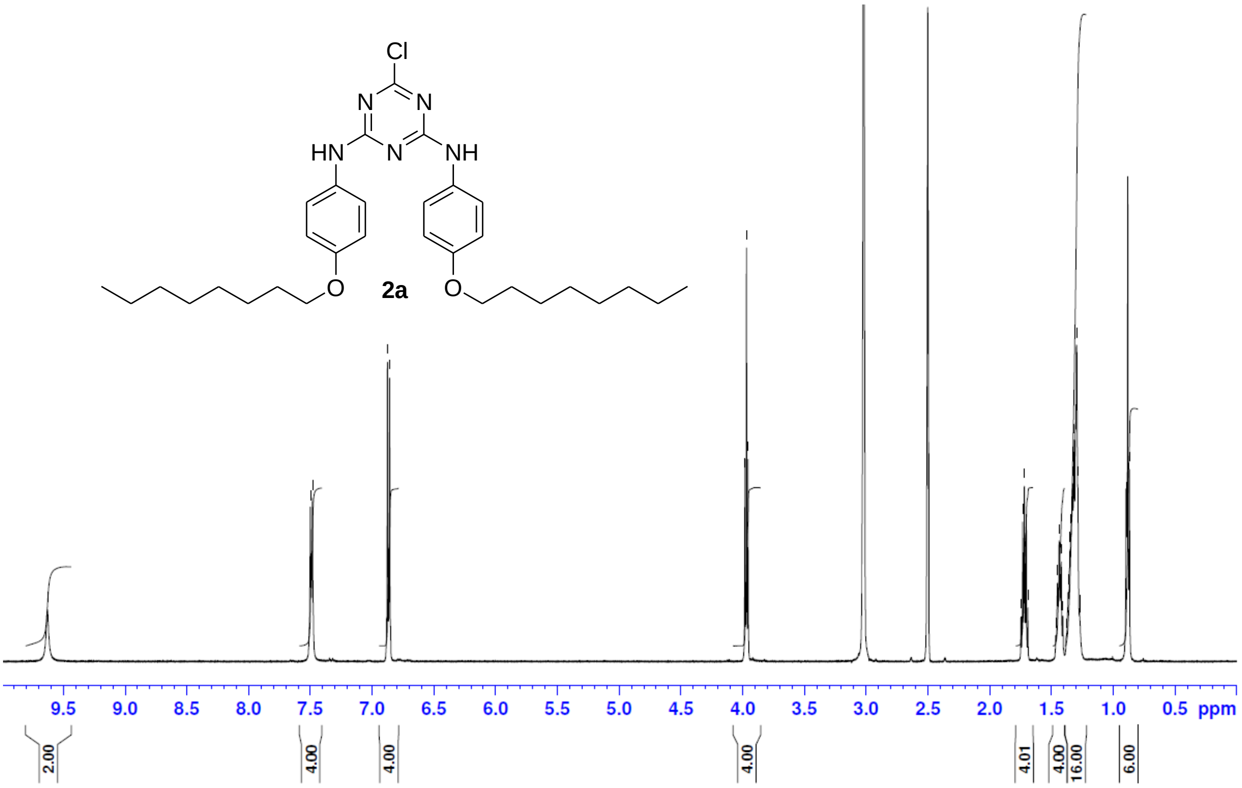

Figure S2: ^{1}H NMR spectrum of compound **2a** (500 MHz, 5.0 mM in DMSO-d_6, 363 K).

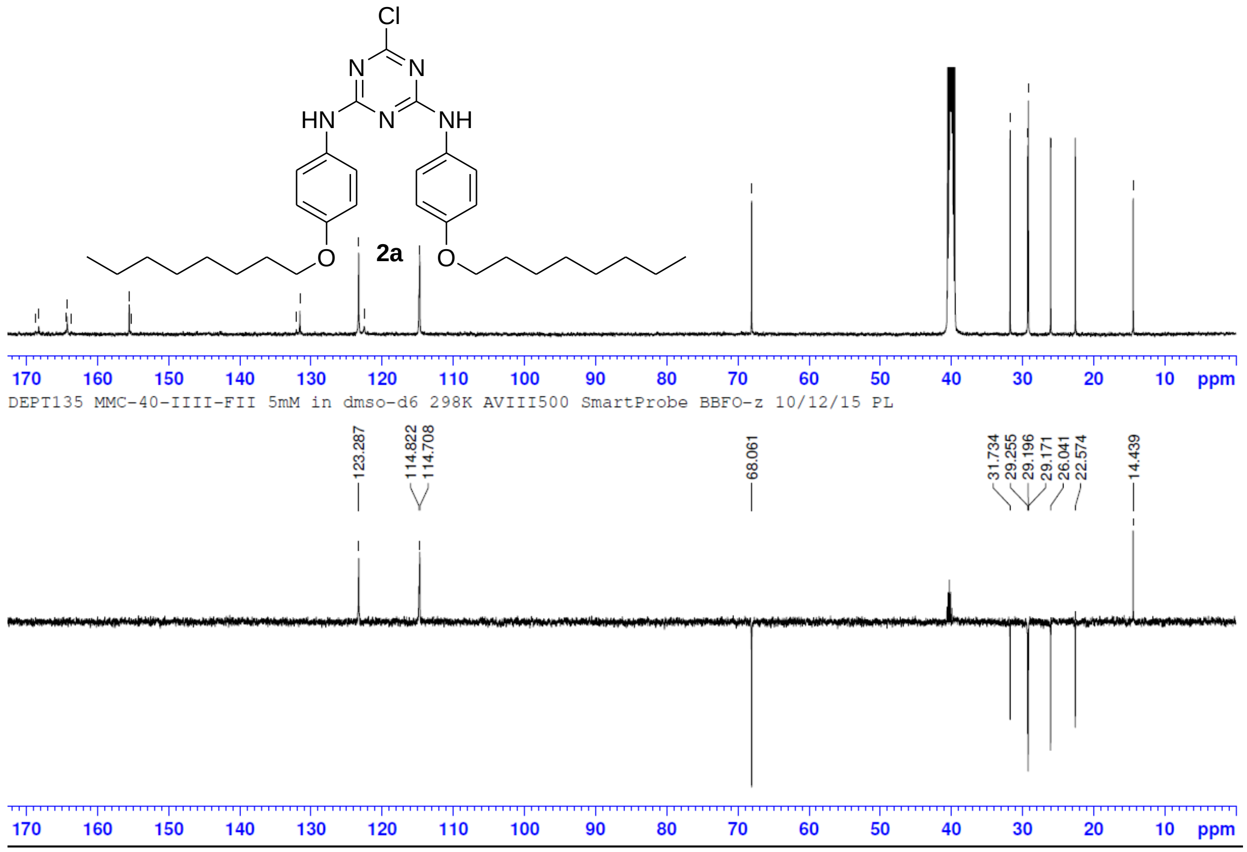

Figure S3: DEPT ^{13}C NMR spectrum of compound **2a** (125 MHz, 5.0 mM in DMSO-d_6, 298 K).

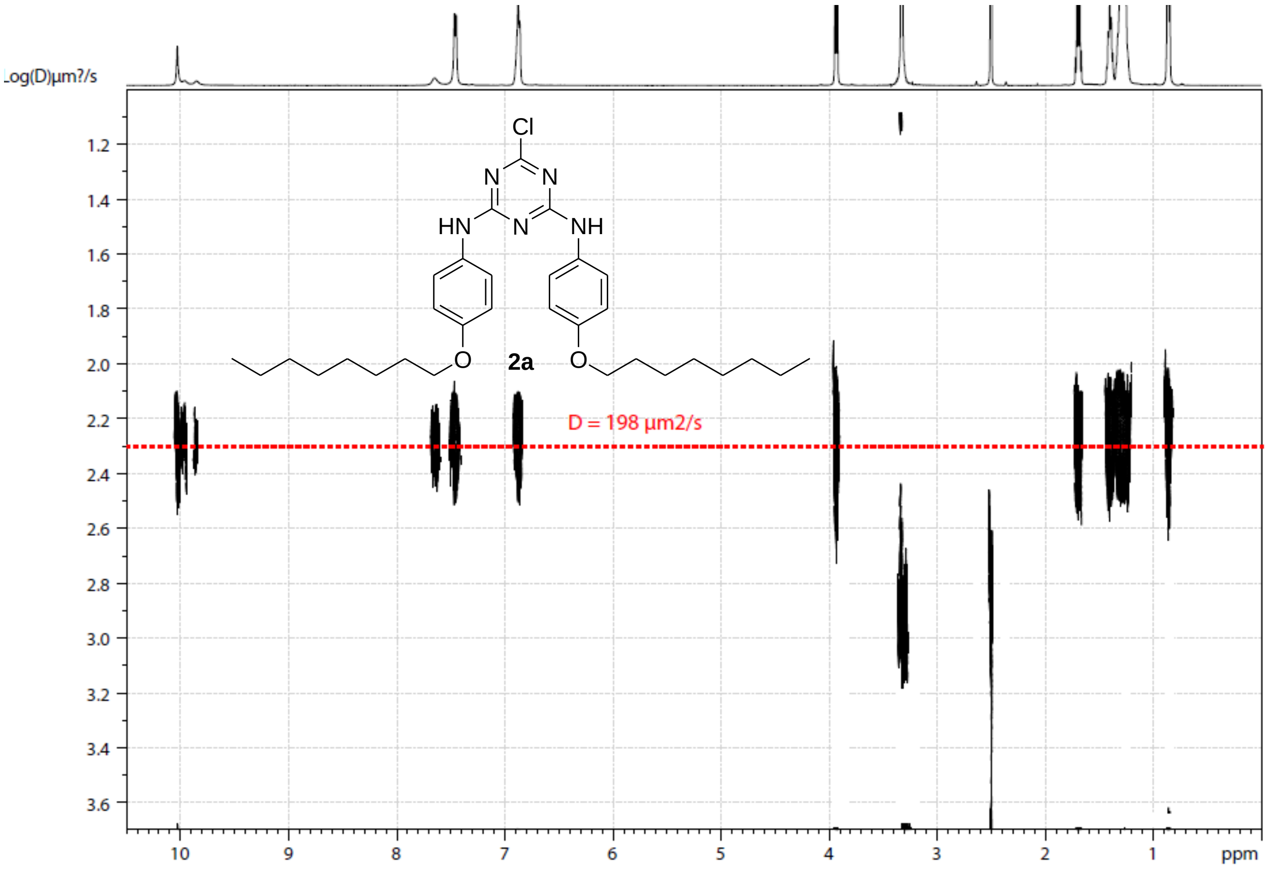

Figure S4: 2D-^{1}H-DOSY NMR chart of compound **2a** (500 MHz, 5.0 mM in DMSO-d_6, 298 K).

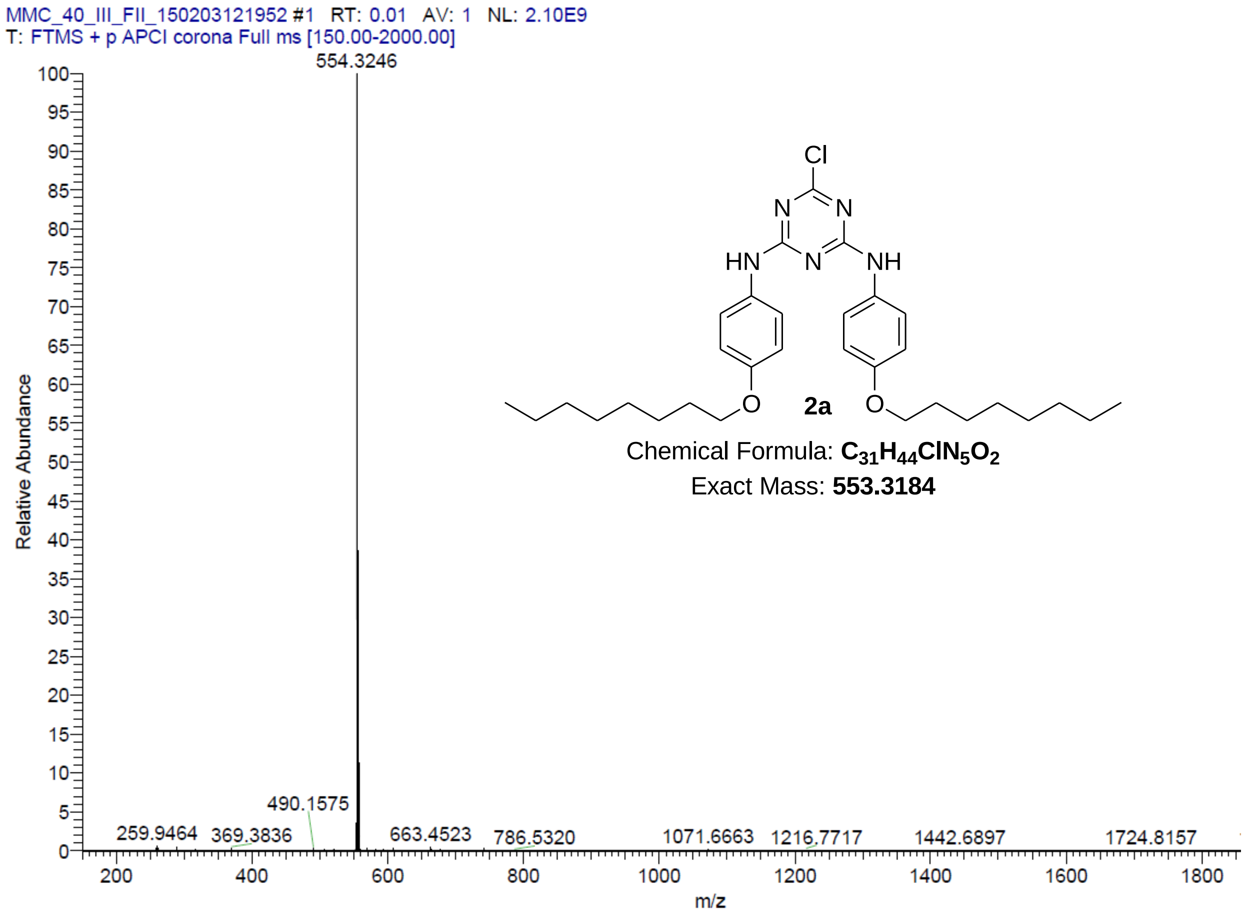

Figure S5: Mass spectrum of compound **2a** [HRMS (APCI+)].

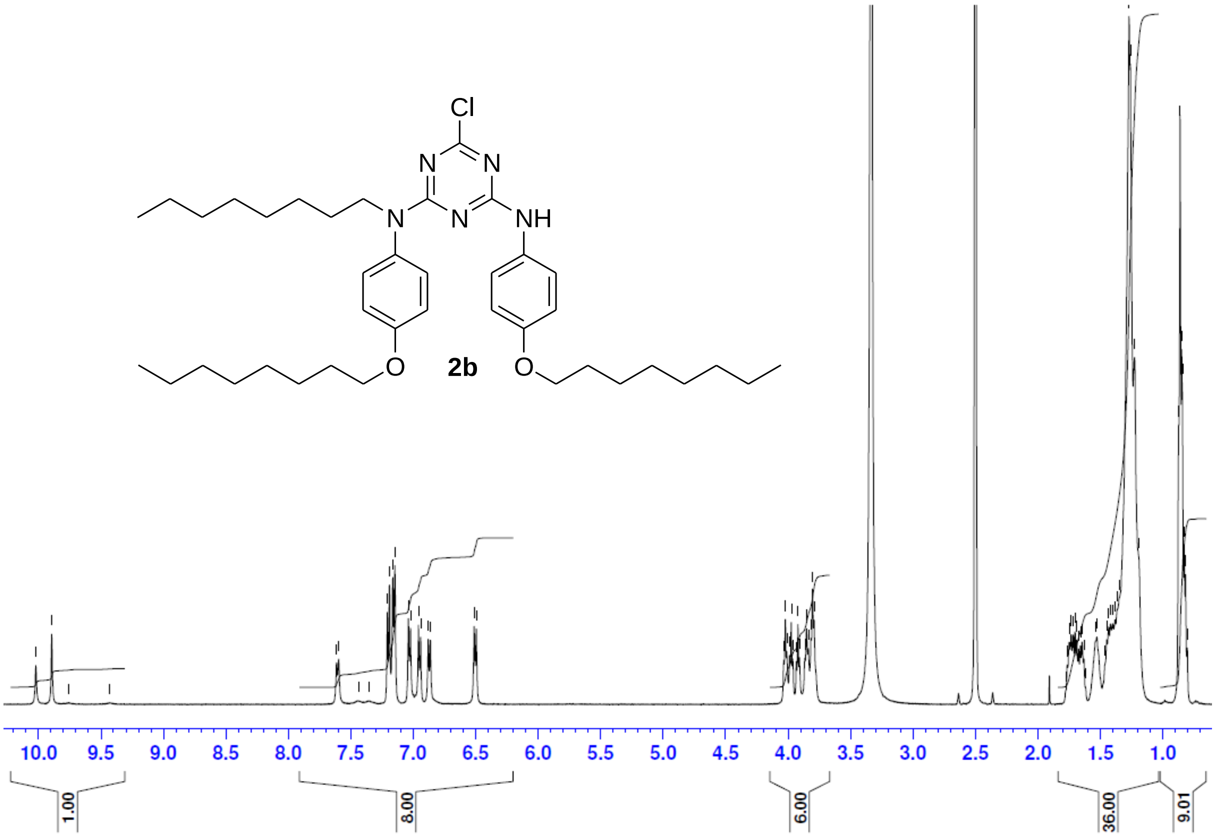

Figure S6: ^{1}H NMR spectrum of compound **2b** (500 MHz, 5.0 mM in DMSO-d_6, 298 K).

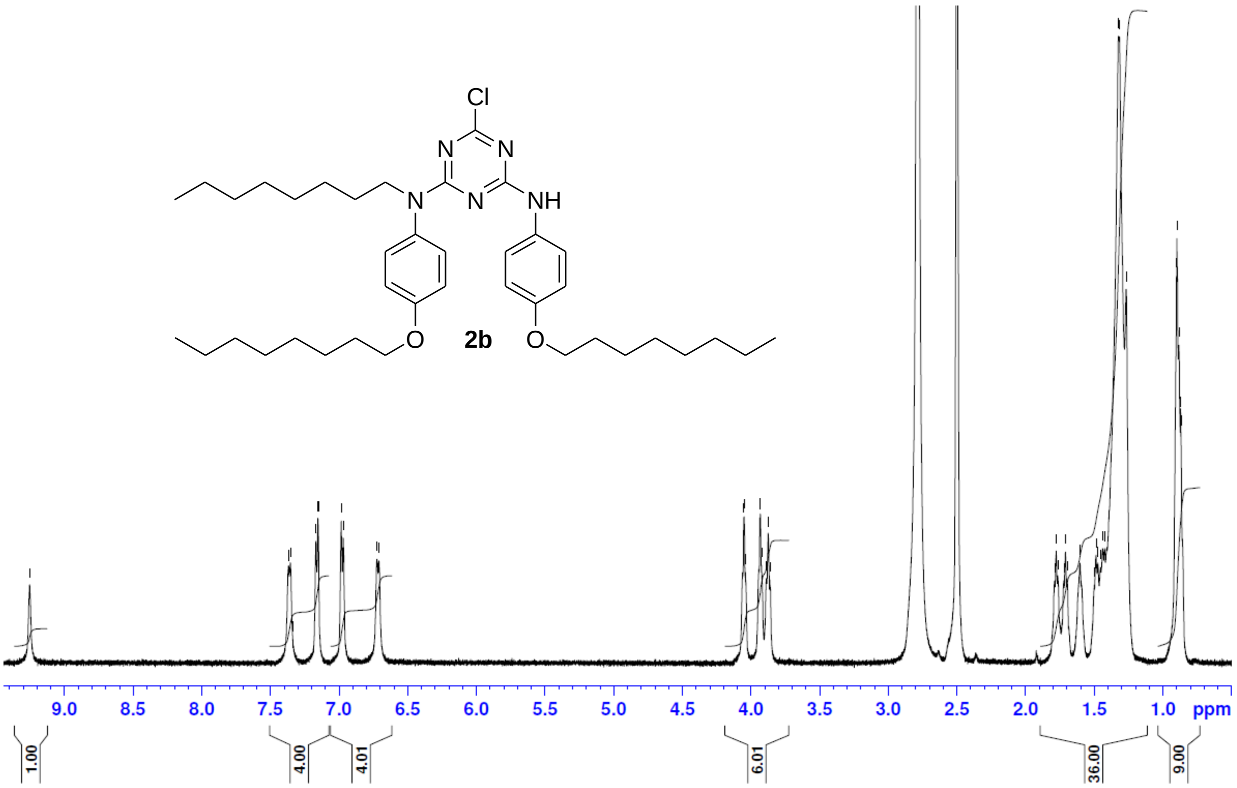

Figure S7: ^{1}H NMR spectrum of compound **2b** (500 MHz, 5.0 mM in DMSO-d_6, 403 K).

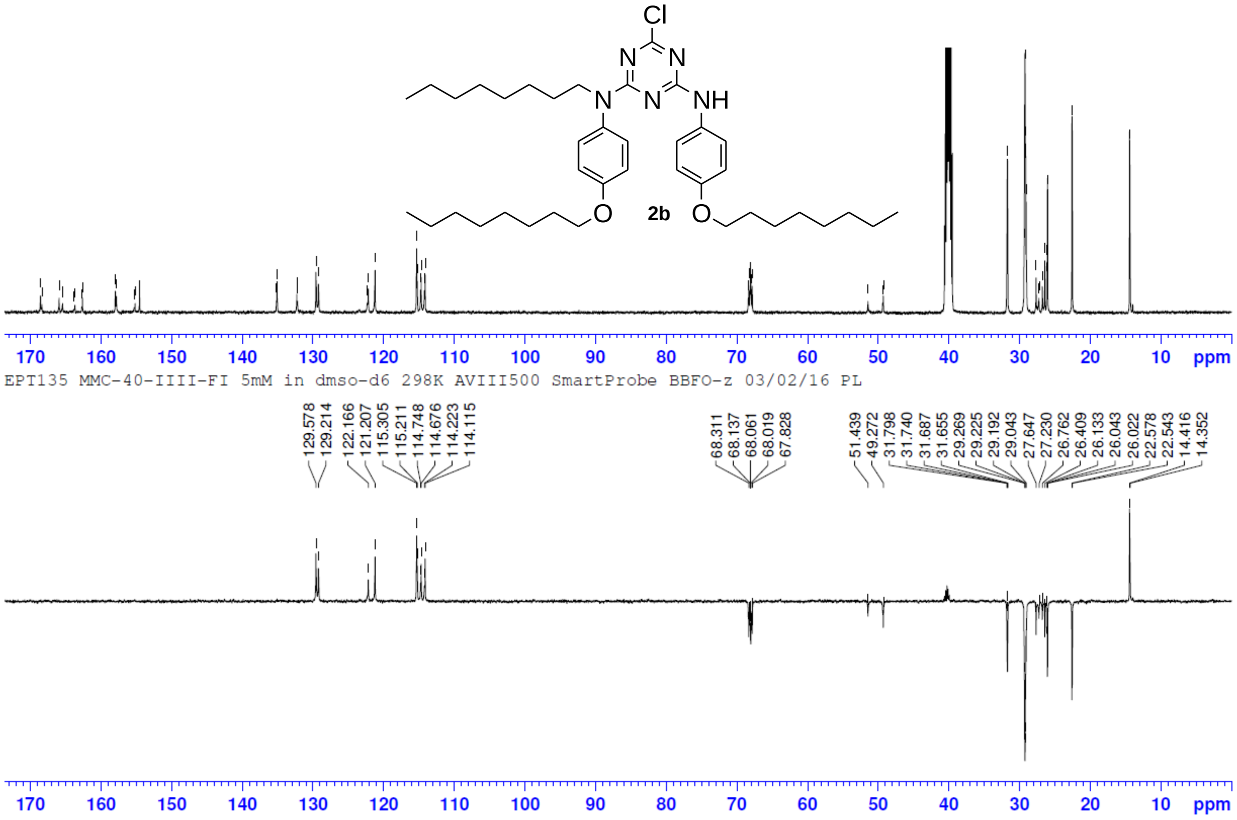

Figure S8: DEPT ^{13}C NMR spectrum of compound **2b** (125 MHz, 5.0 mM in DMSO-d_6, 298 K).

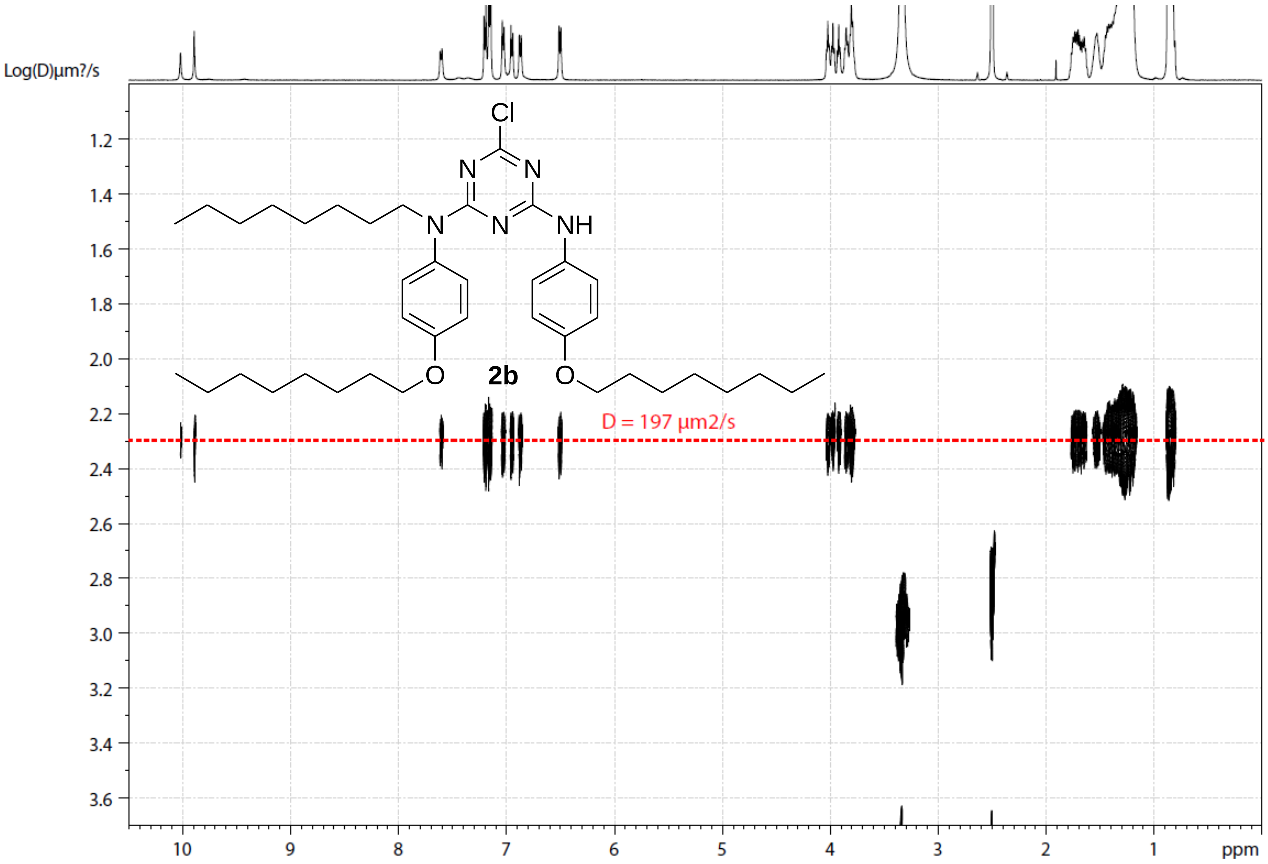

Figure S9: 2D-^{1}H-DOSY NMR chart of compound **2b** (500 MHz, 5.0 mM in DMSO-d_6, 298 K).

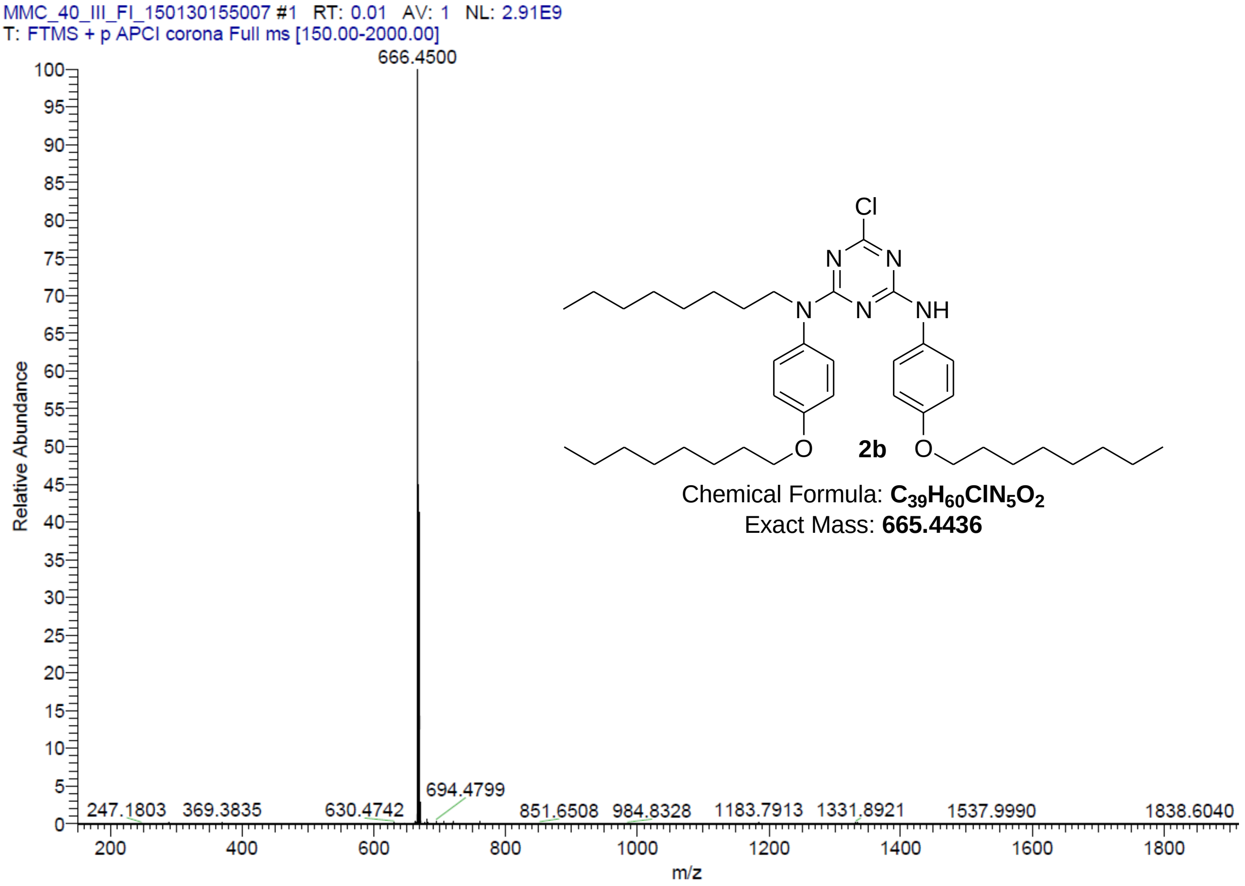

Figure S10: Mass spectrum of compound **2b** [HRMS (APCI+)].

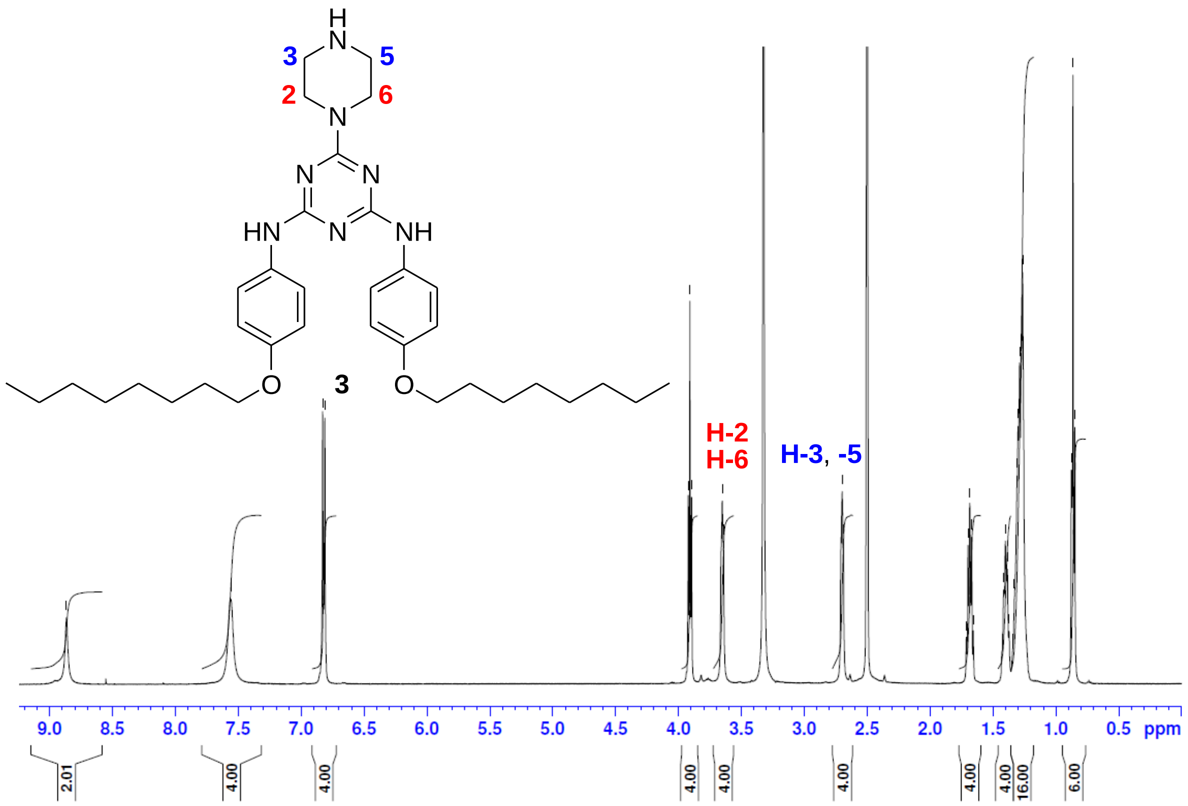

Figure S11: ^{1}H NMR spectrum of compound **3** (500 MHz, 5.0 mM in DMSO-d_6, 298 K).

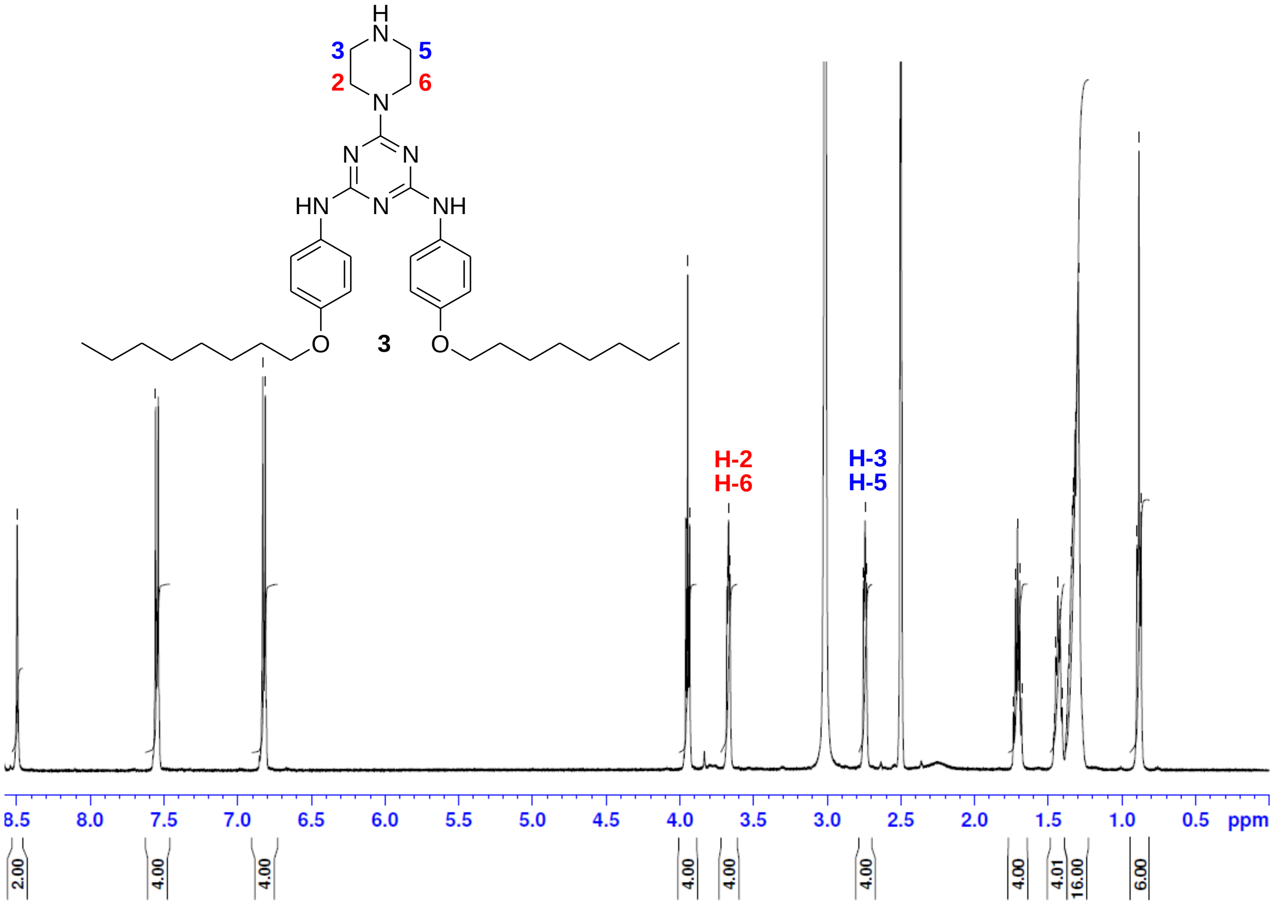

Figure S12: ^{1}H NMR spectrum of compound **3** (500 MHz, 5.0 mM in DMSO-d_6, 363 K).

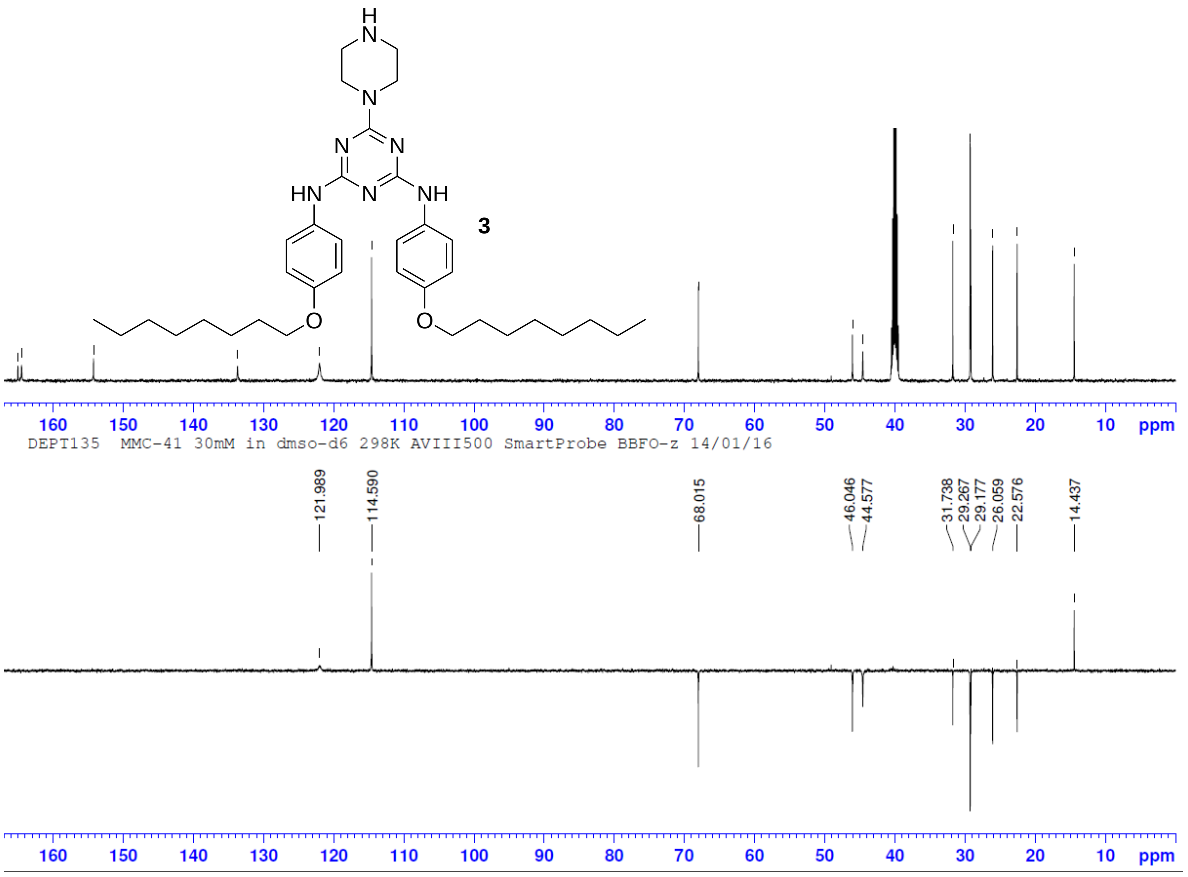

Figure S13: DEPT ^{13}C NMR spectrum of compound **3** (125 MHz, 5.0 mM in DMSO-d_6, 298 K).

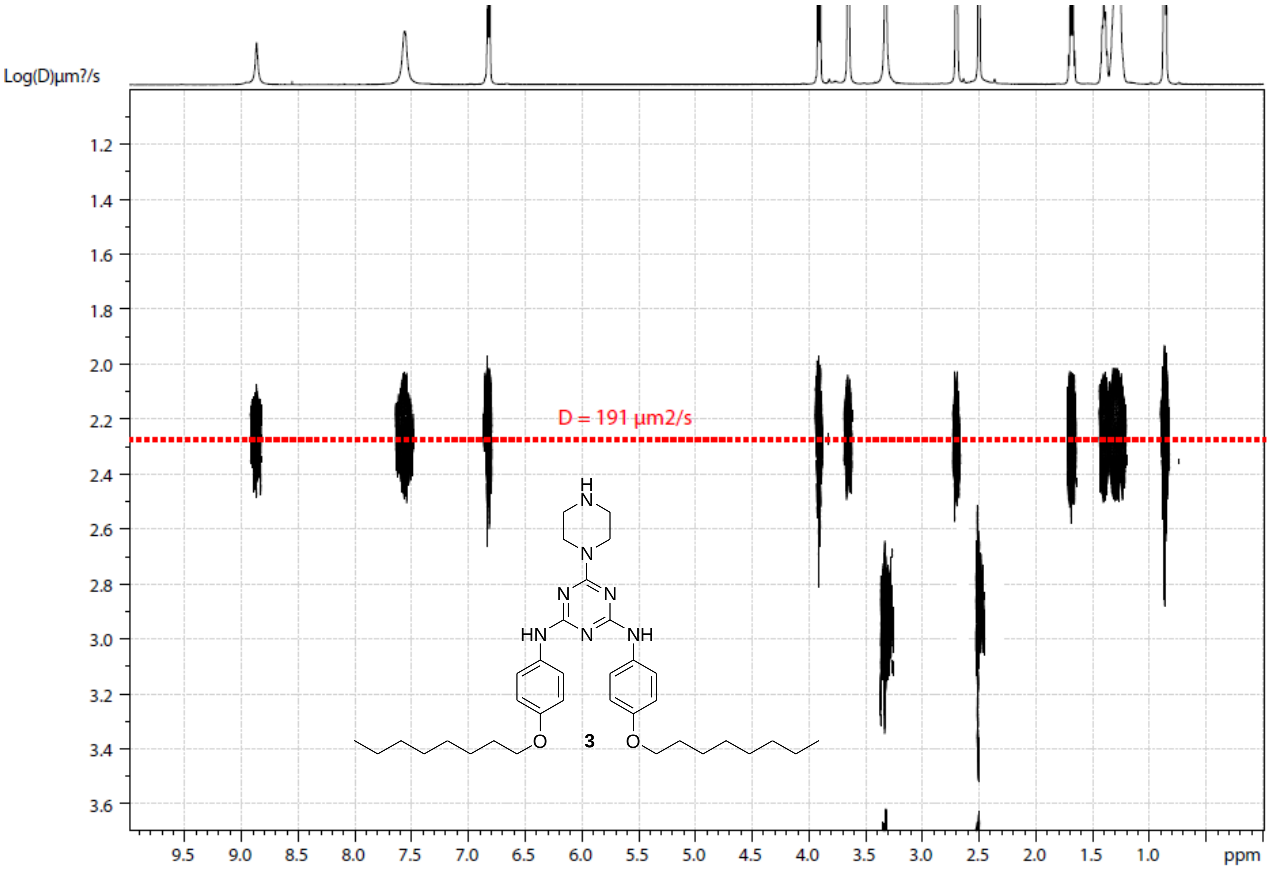

Figure S14: 2D-^{1}H-DOSY NMR chart of compound **3** (500 MHz, 5.0 mM in DMSO-d_6, 298 K).

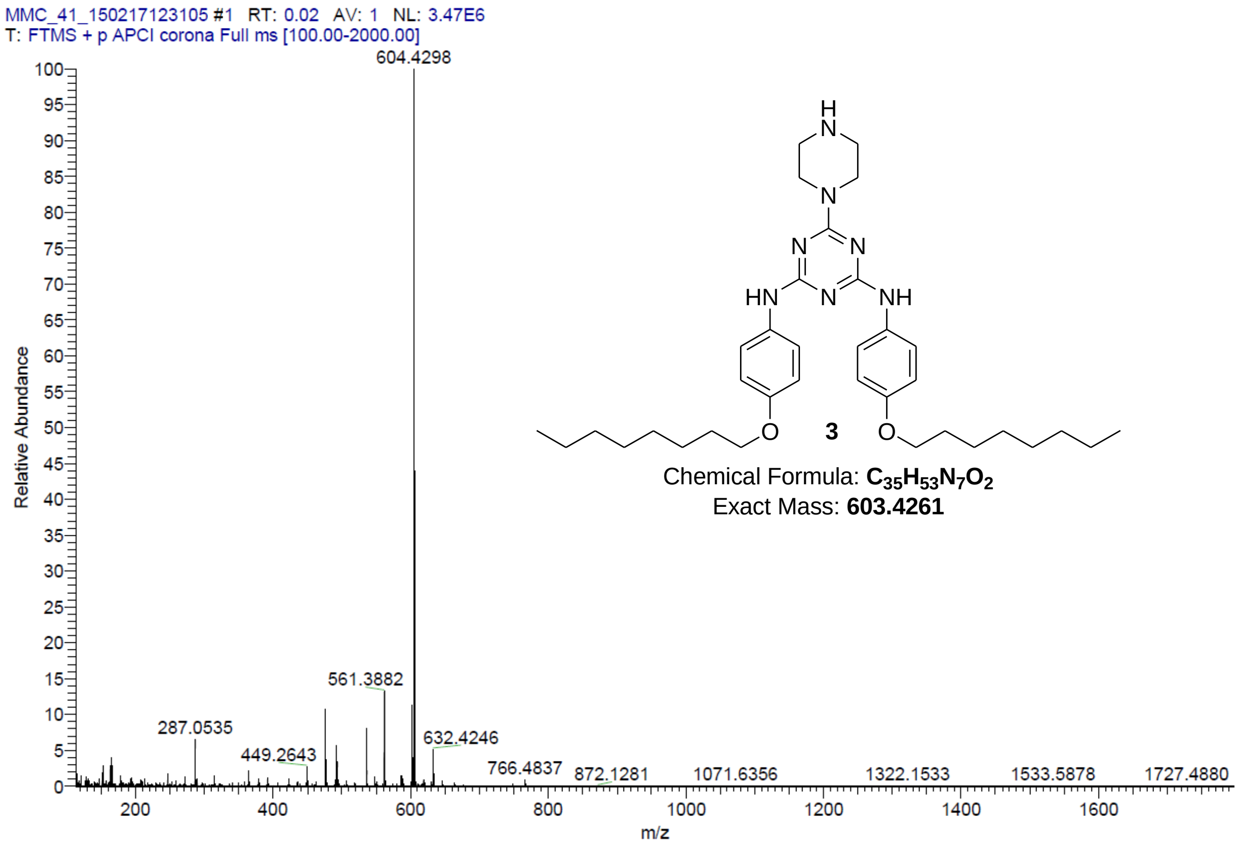

Figure S15: Mass spectrum of compound **3** [HRMS (APCI+)].

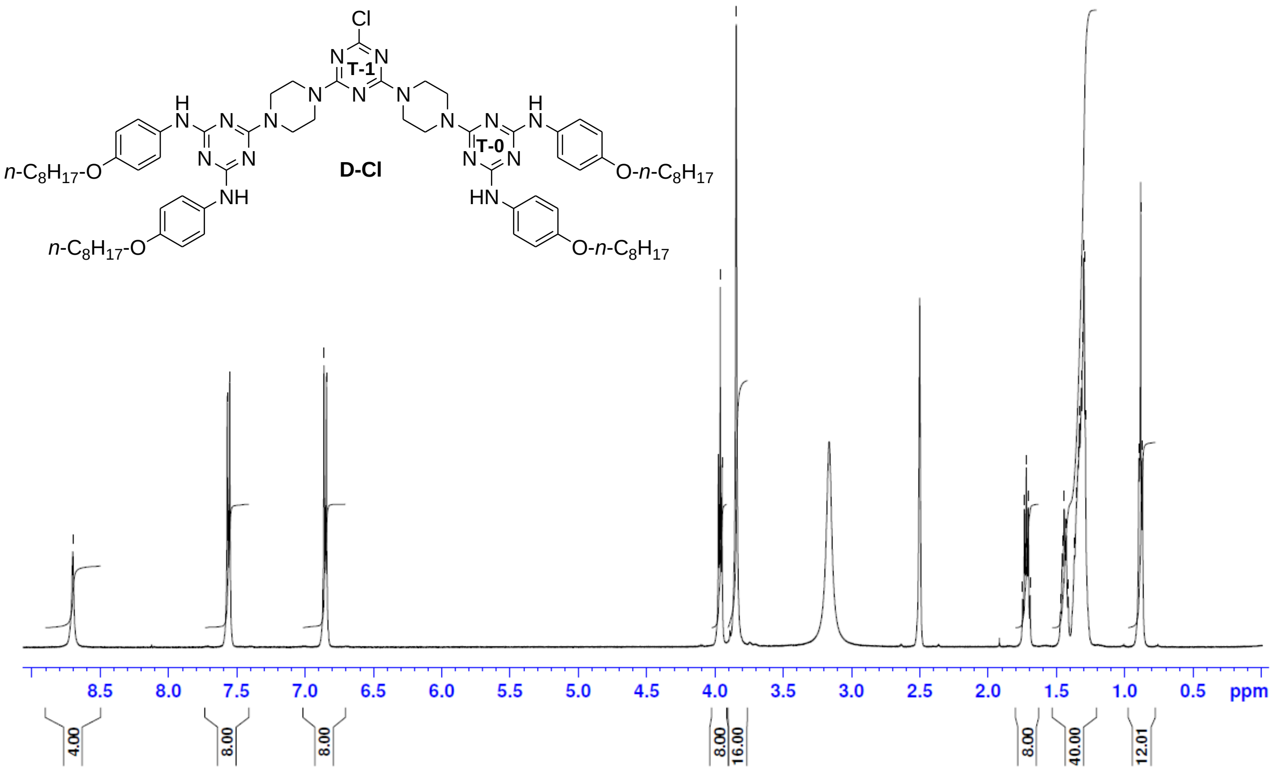

Figure S16: ^{1}H NMR spectrum of compound **D-Cl** (500 MHz, 5.0 mM in DMSO-d_6, 363 K).

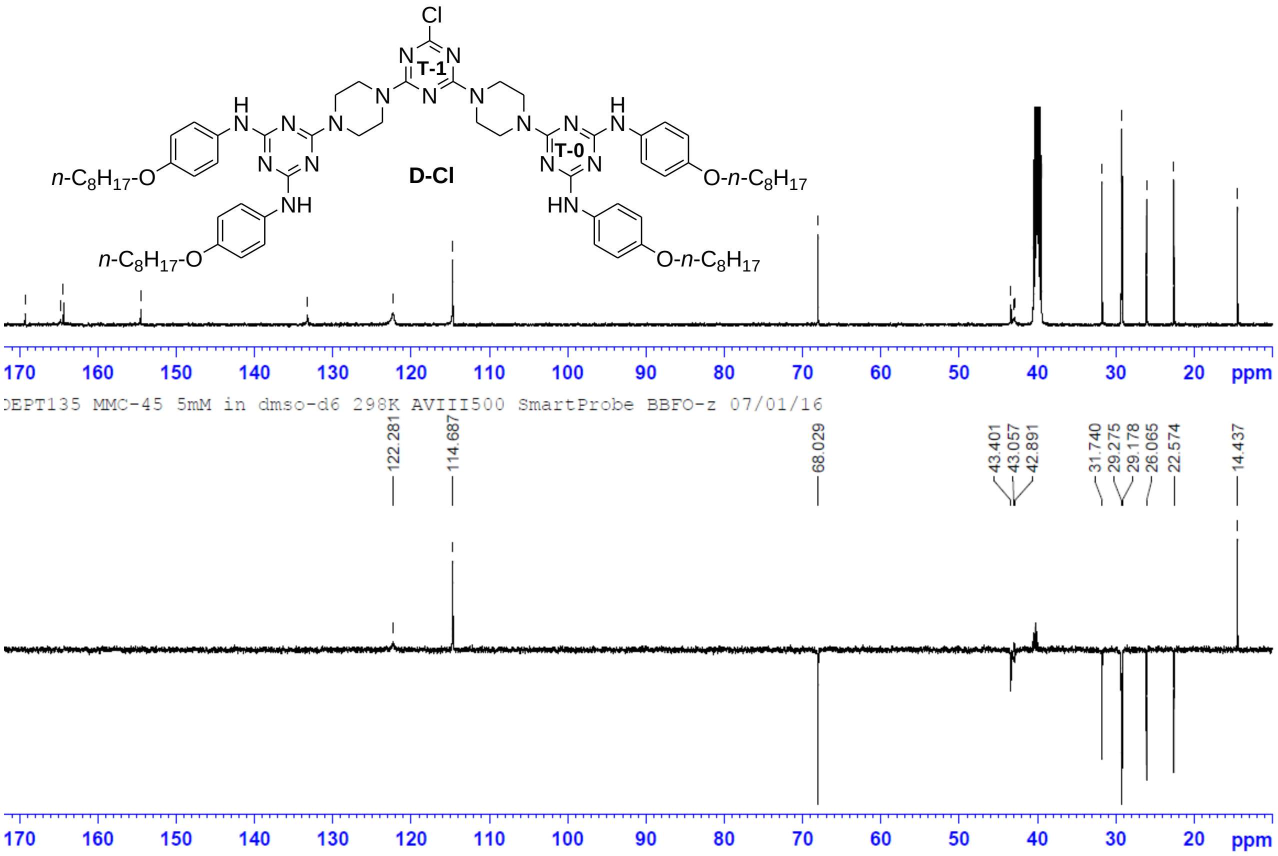

Figure S17: DEPT ^{13}C NMR spectrum of compound **D-Cl** (125 MHz, 5.0 mM in DMSO-d_6, 298 K).

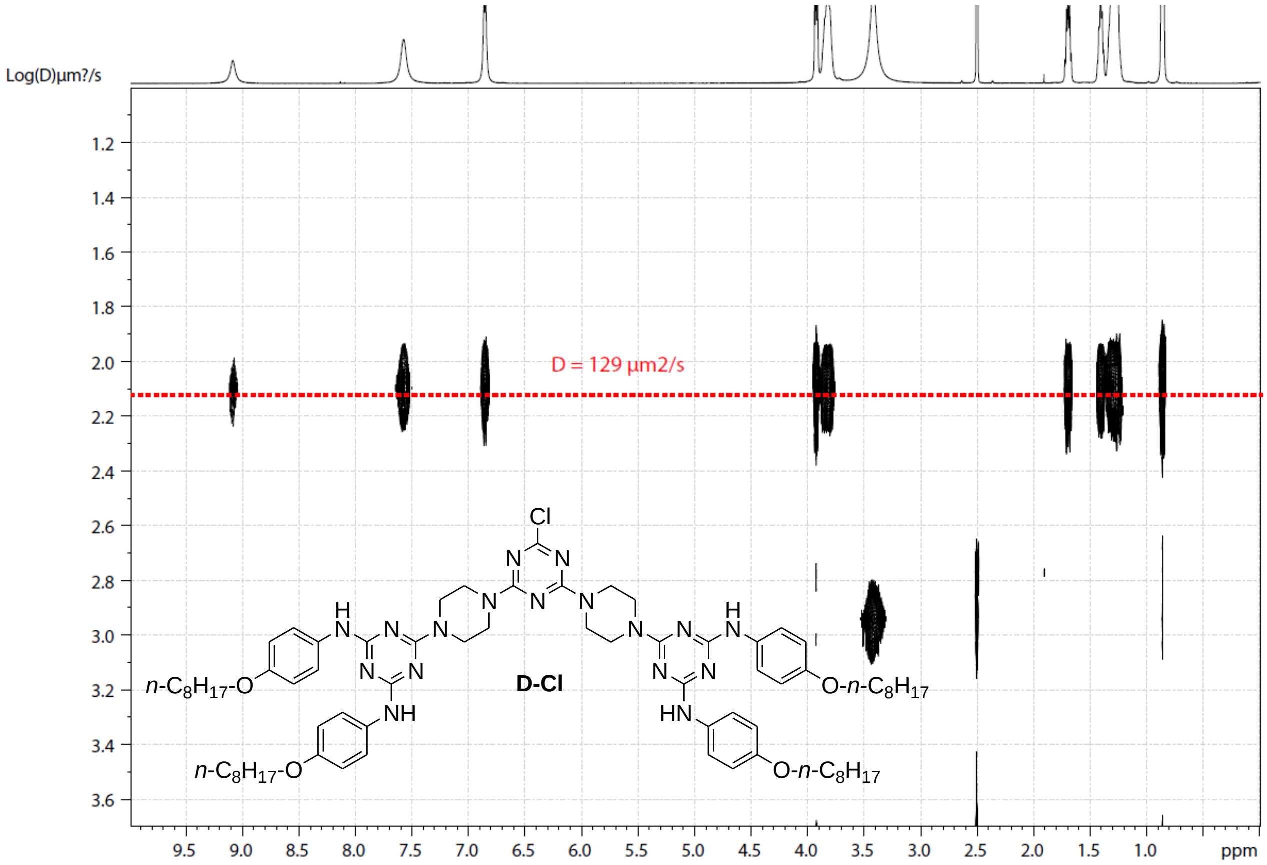

Figure S18: 2D-^{1}H-DOSY NMR chart of compound **D-Cl** (500 MHz, 5.0 mM in DMSO-d_6, 298 K).

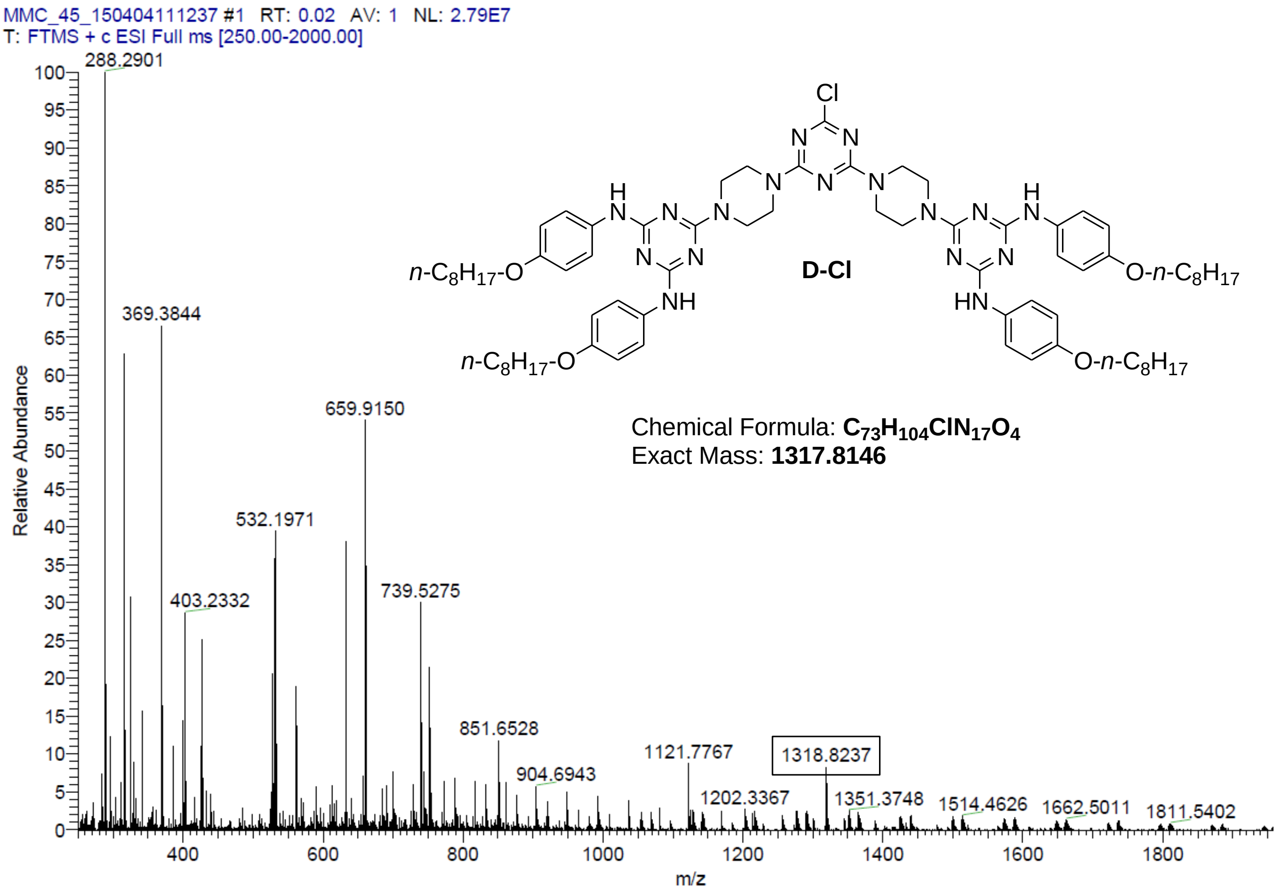

Figure S19: Mass spectrum of compound **D-Cl** [HRMS (ESI+), ACN+TFA].

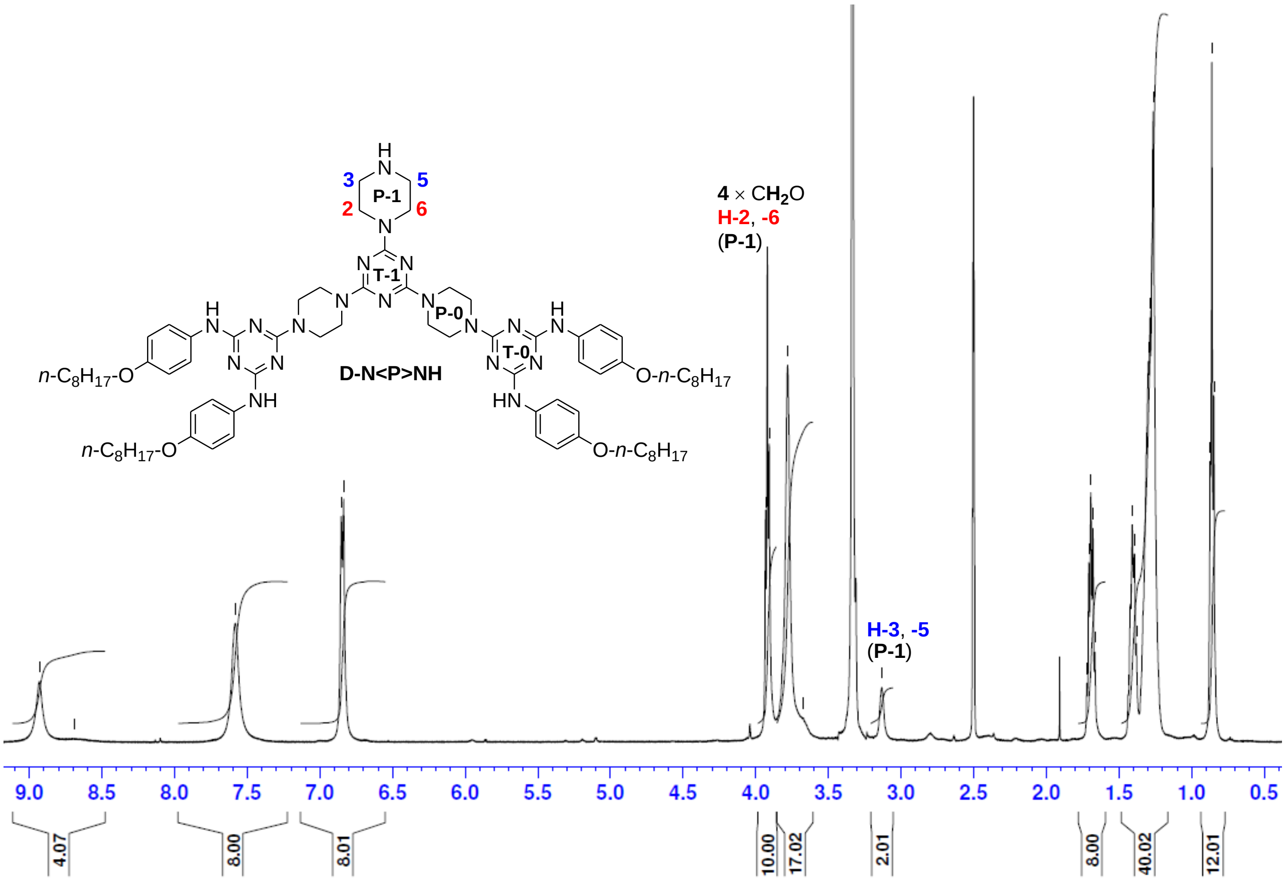

Figure S20: ^{1}H NMR spectrum of compound **D-N<P>NH** (500 MHz, 5.0 mM in DMSO-d_6, 298 K).

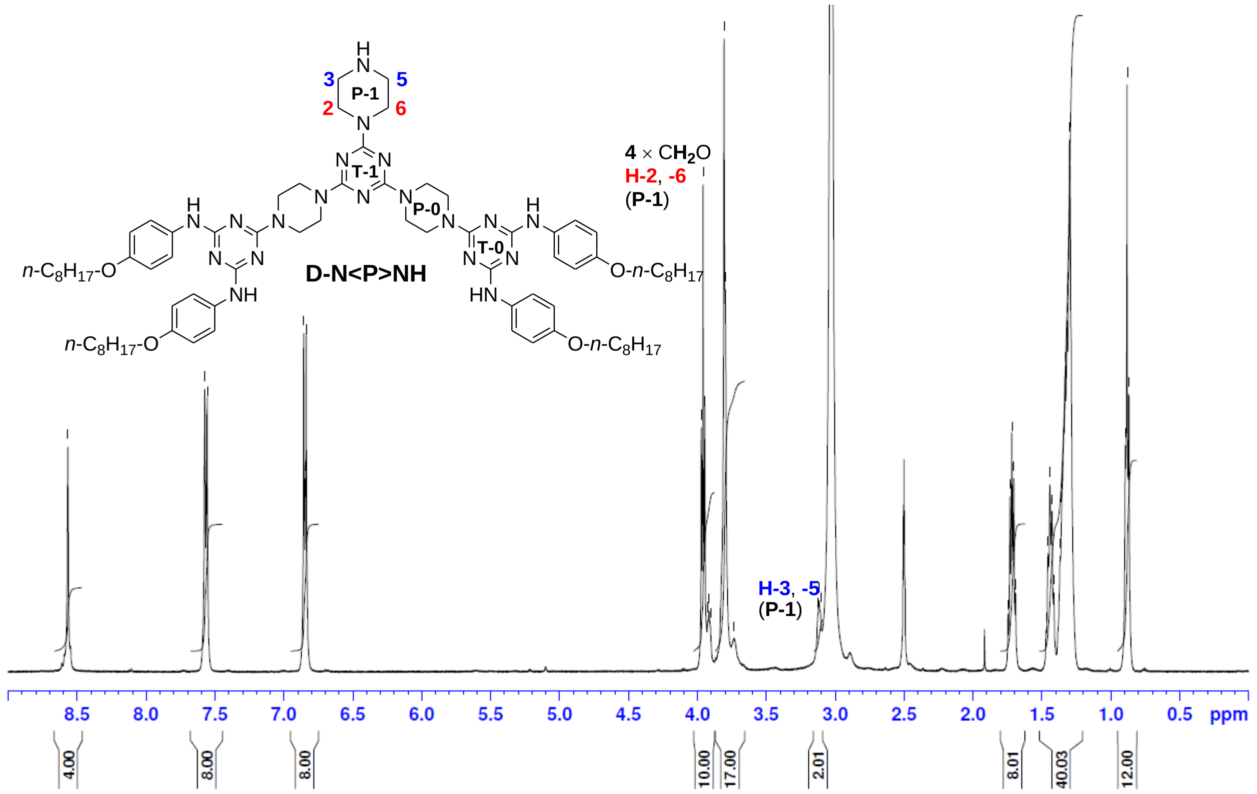

Figure S21: ^{1}H NMR spectrum of compound **D-N<P>NH** (500 MHz, 5.0 mM in DMSO-d_6, 363 K).

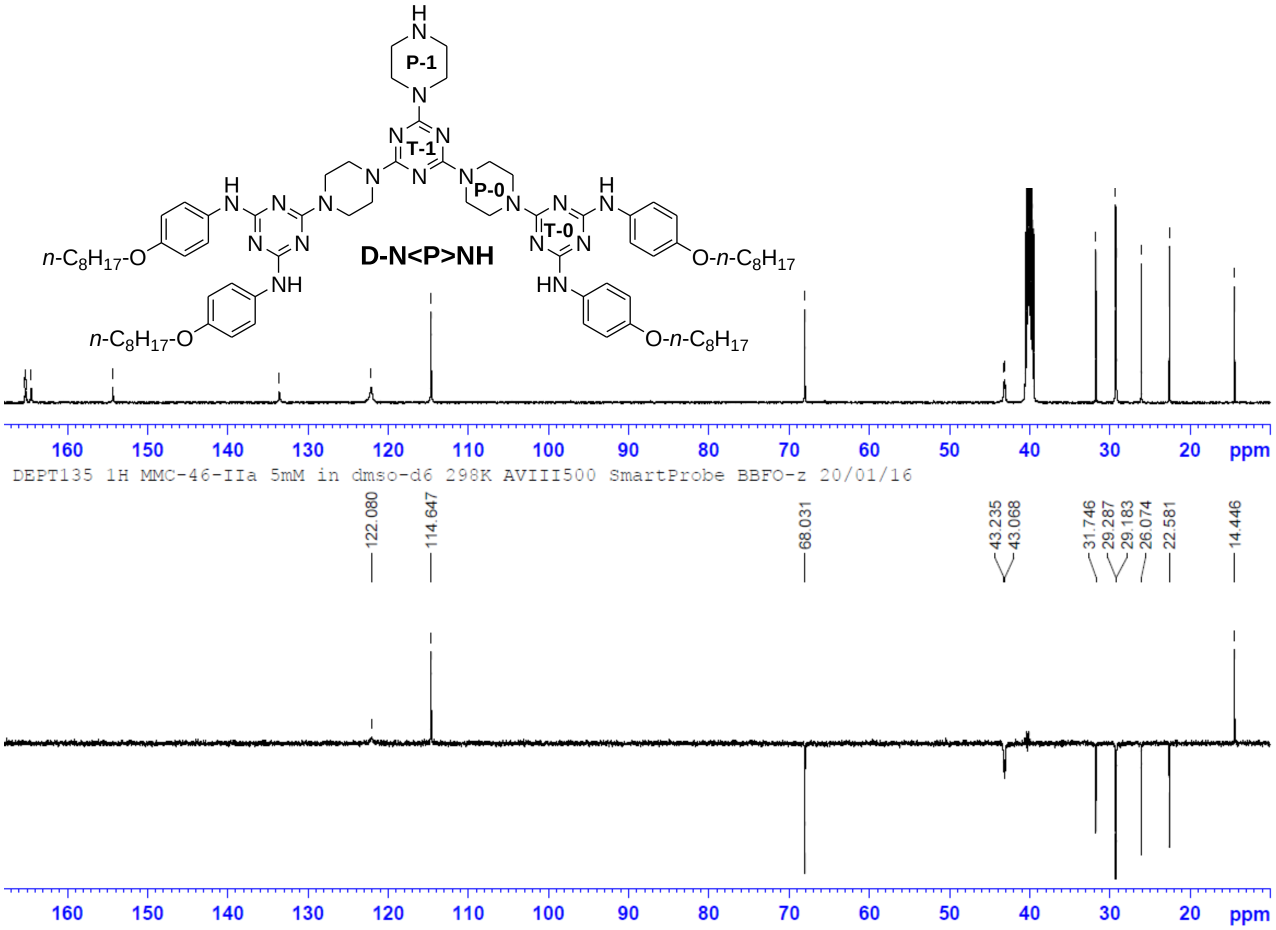

Figure S22: DEPT ^{13}C NMR spectrum of compound **D-N<P>NH** (125 MHz, 5.0 mM in DMSO-d_6, 298 K).

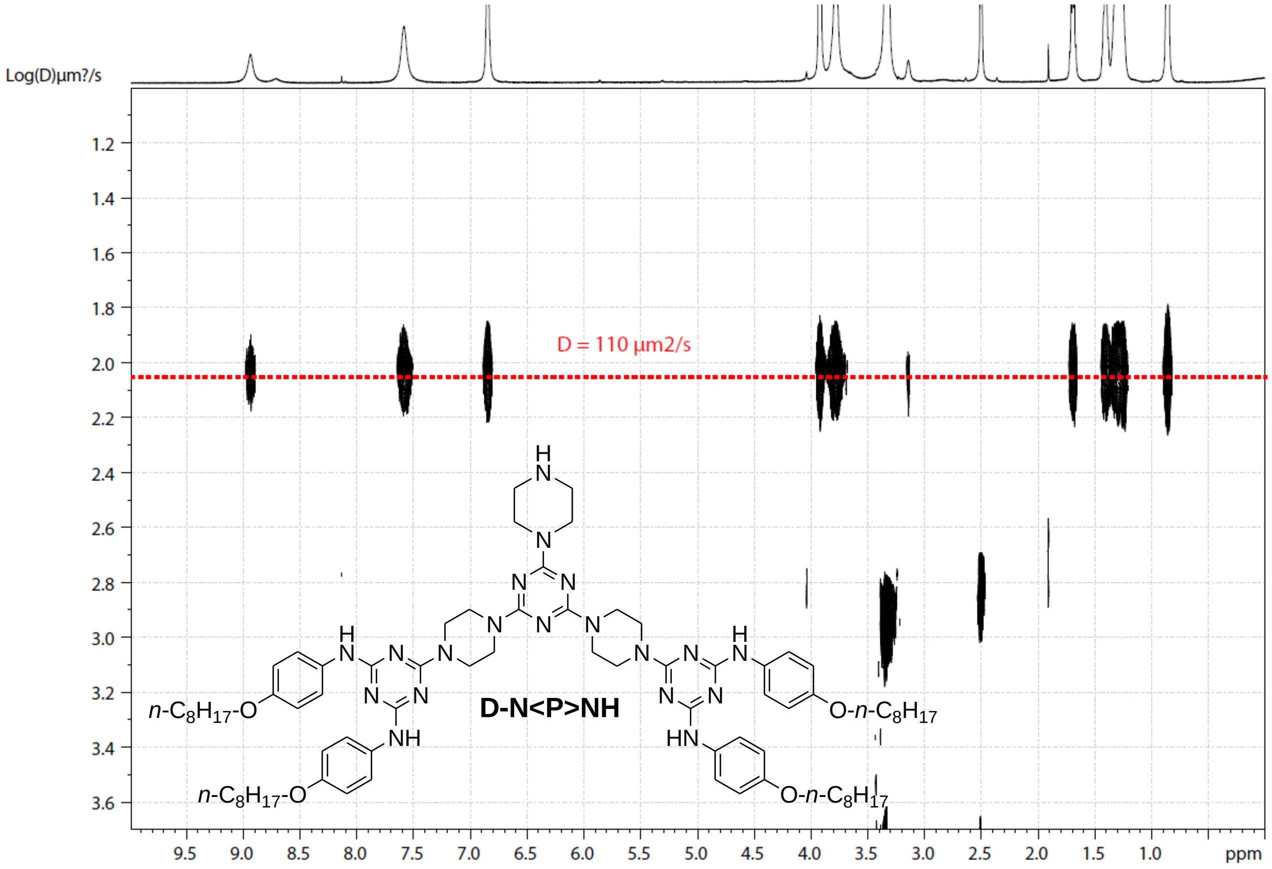

Figure S23: 2D-^{1}H-DOSY NMR chart of compound **D-N<P>NH** (500 MHz, 5.0 mM in DMSO-d_6, 298 K).

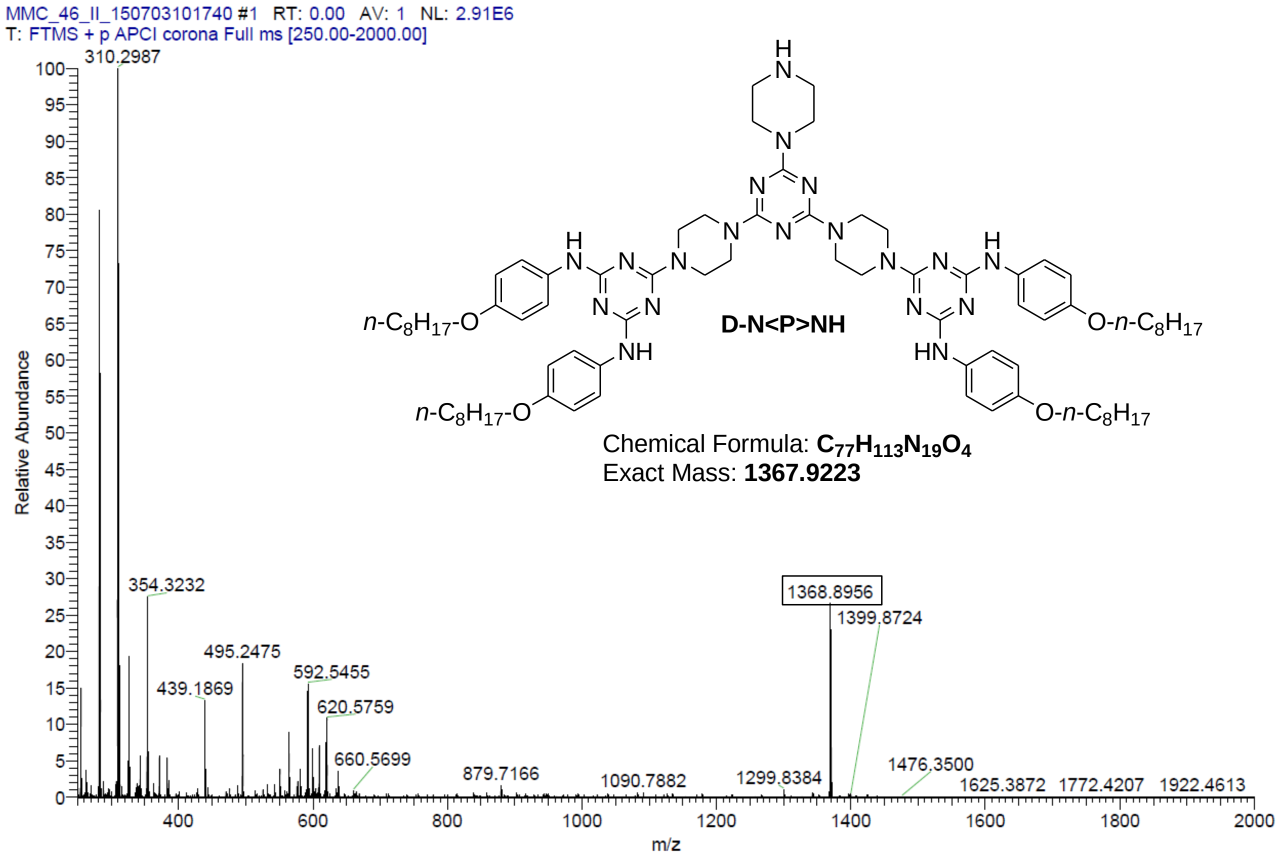

Figure S24: Mass spectrum of compound **D-N<P>NH** [HRMS (APCI+)].

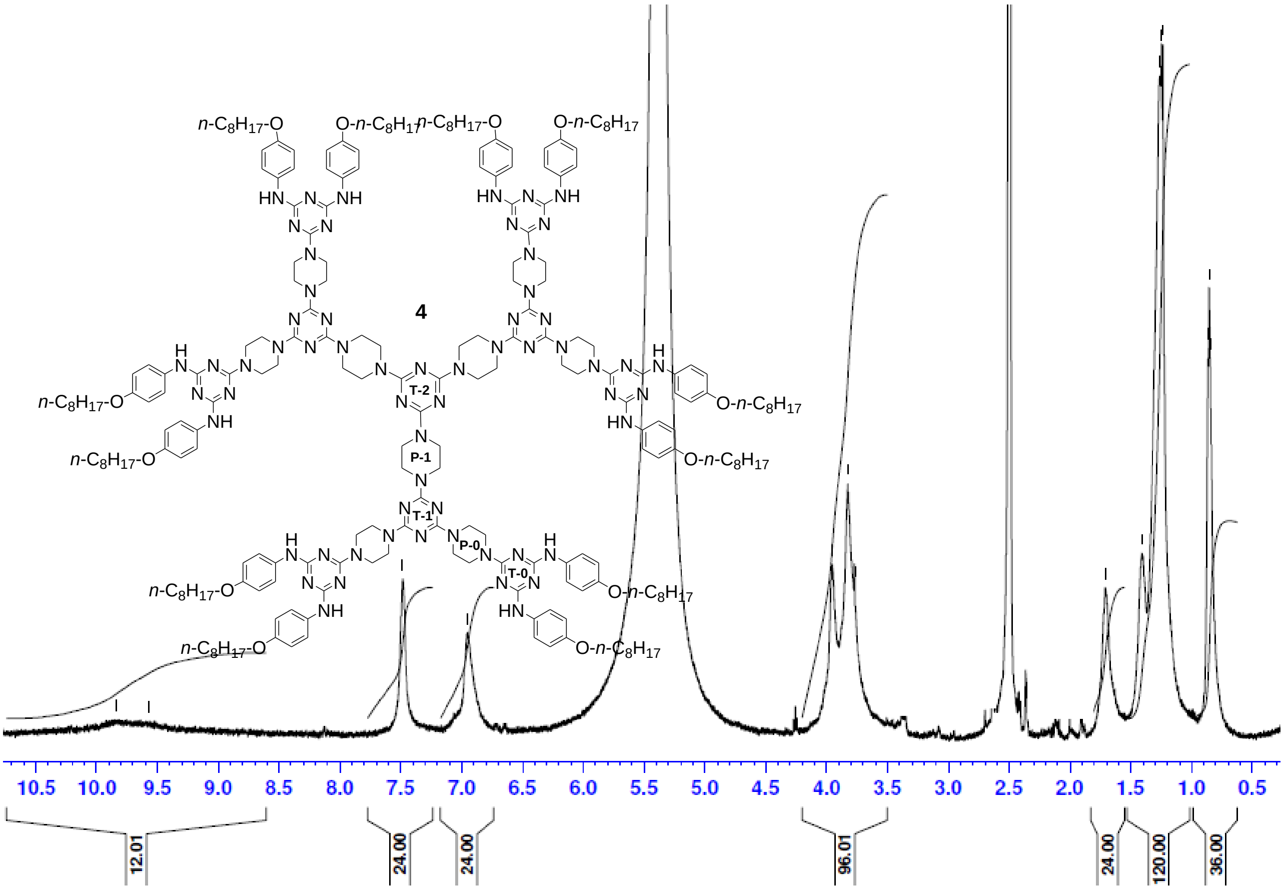

Figure S25: ^{1}H NMR spectrum of compound **4** (500 MHz, 2.5 mM in DMSO-d_6, 298 K).

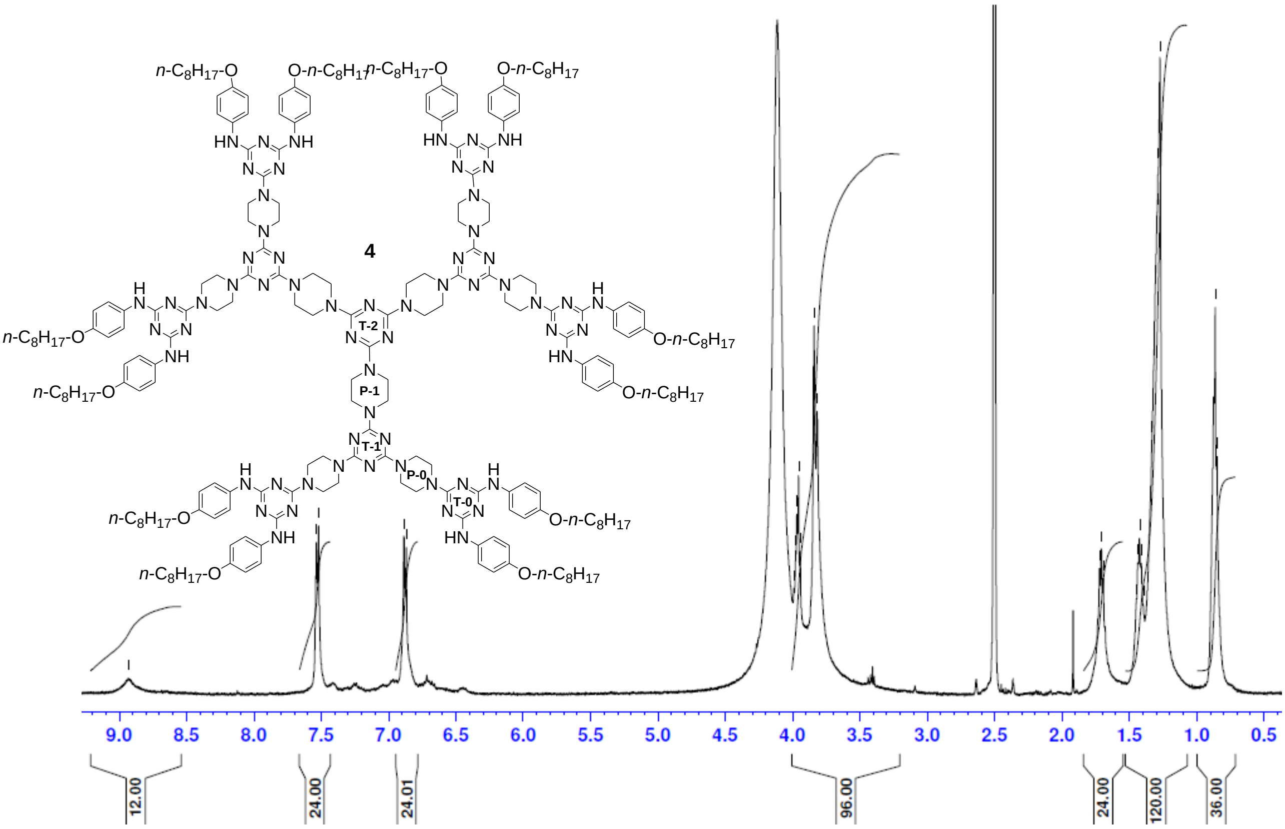

Figure S26: ^{1}H NMR spectrum of compound **4** (500 MHz, 2.5 mM in DMSO-d_6, 363 K).

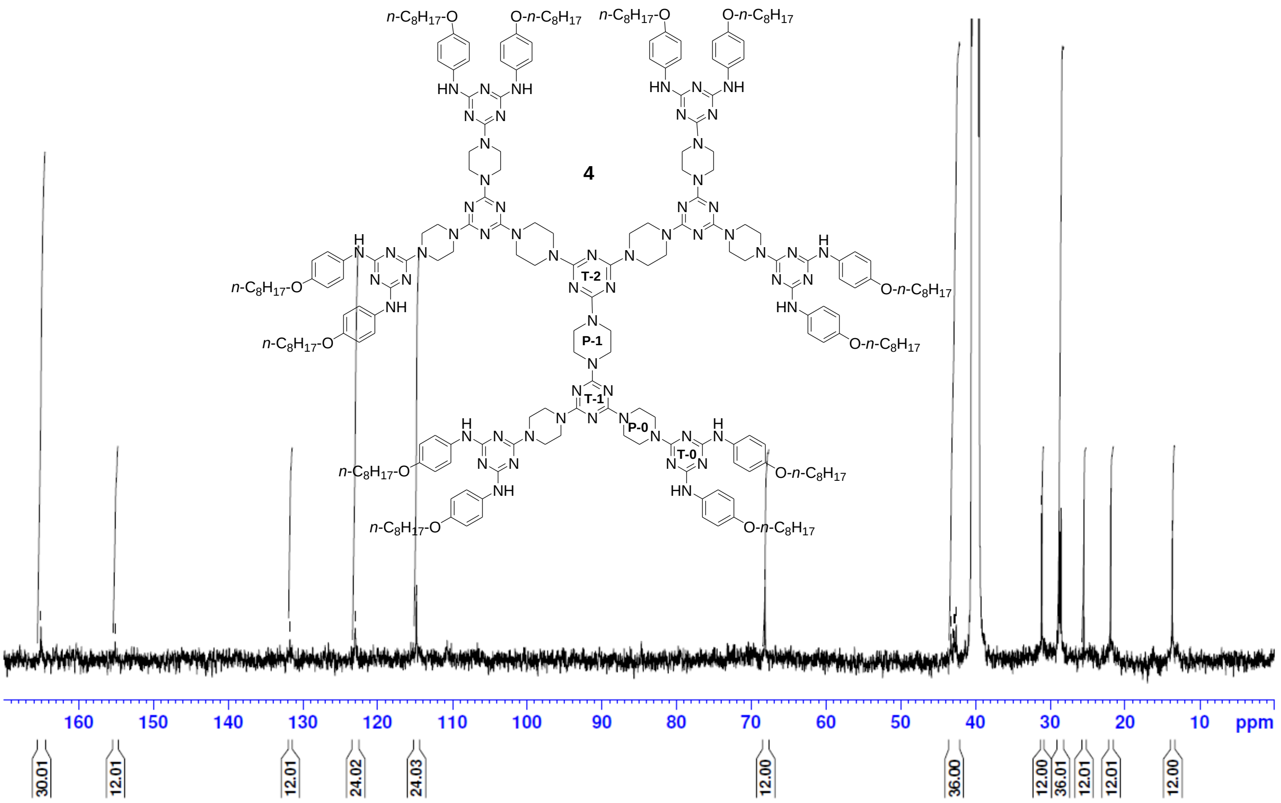

Figure S27: ^{13}QC NMR of compound **4** (125 MHz, 2.5 mM in DMSO-d_6, 363 K).

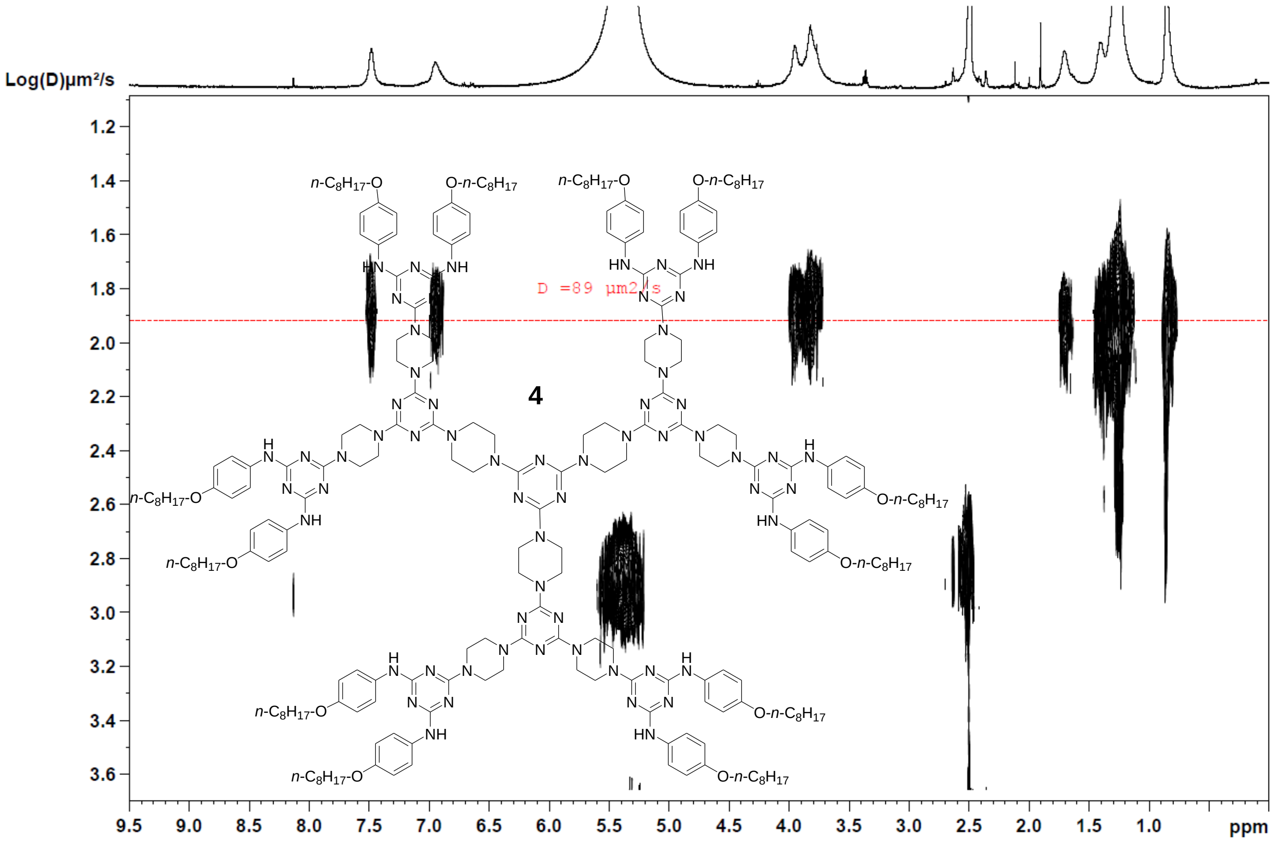

Figure S28: 2D-^{1}H-DOSY NMR chart of compound **4** (500 MHz, 2.5 mM in DMSO-d_6, 298 K).

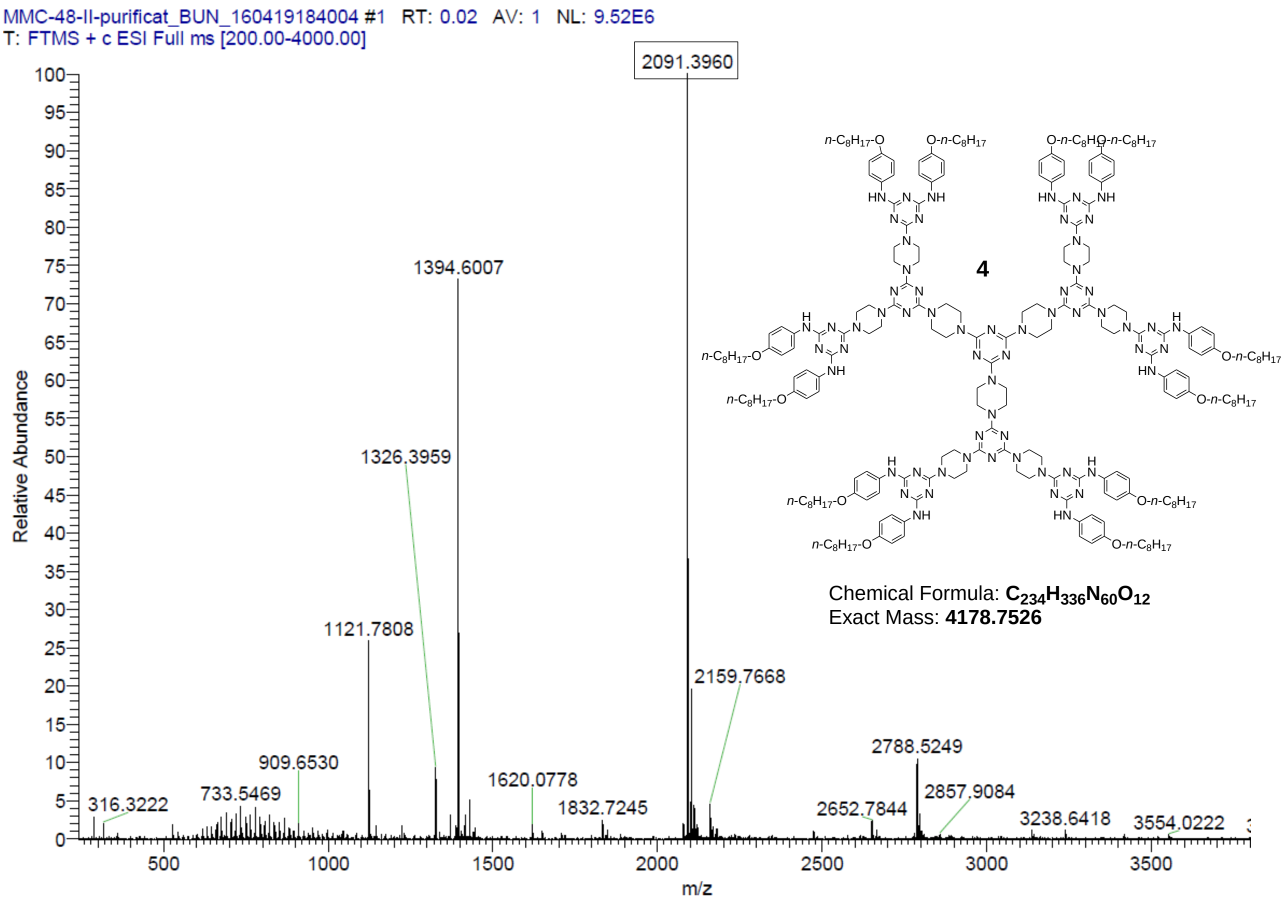

Figure S29: Mass spectrum of compound **4** [HRMS (ESI+) ACN+TFA].

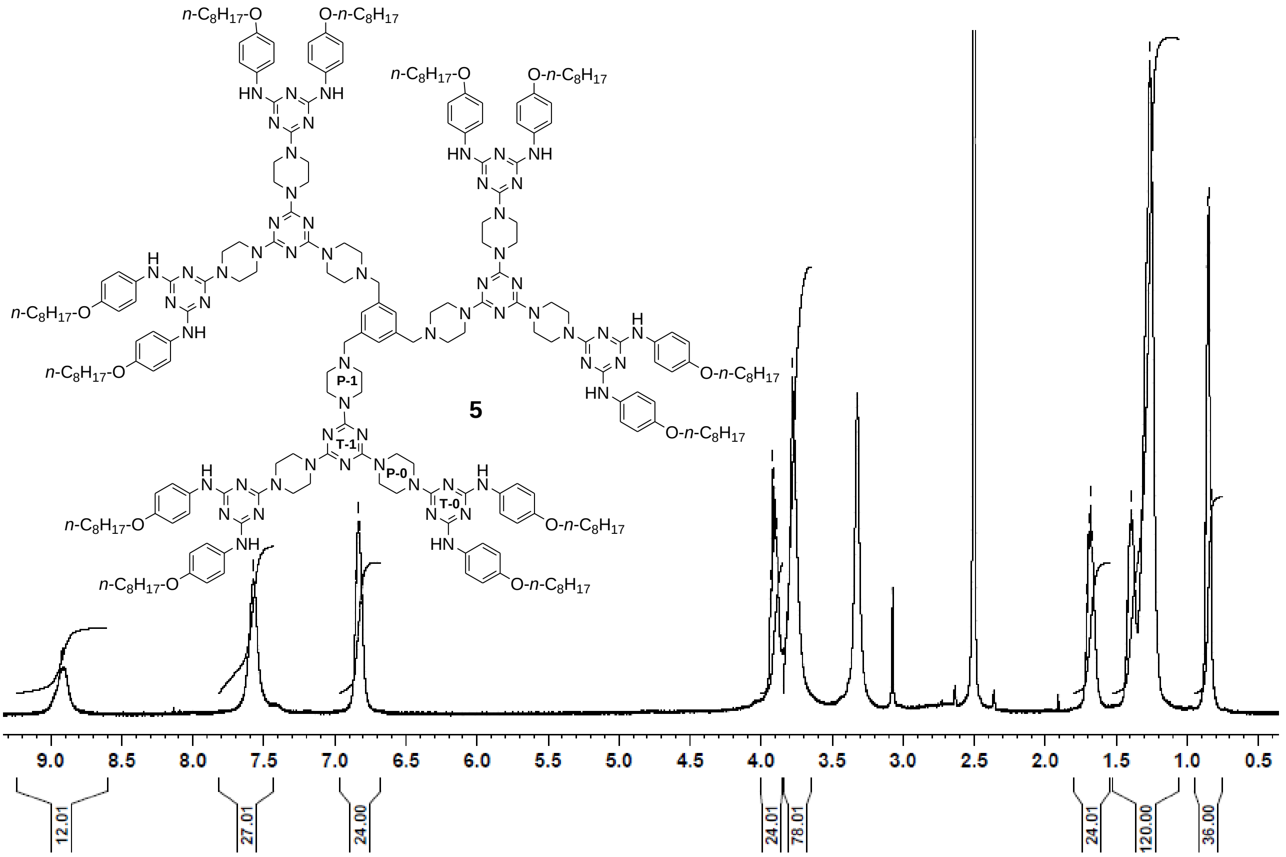

Figure S30: ^{1}H NMR spectrum of compound **5** (500 MHz, 5.0 mM in DMSO-d_6, 298 K).

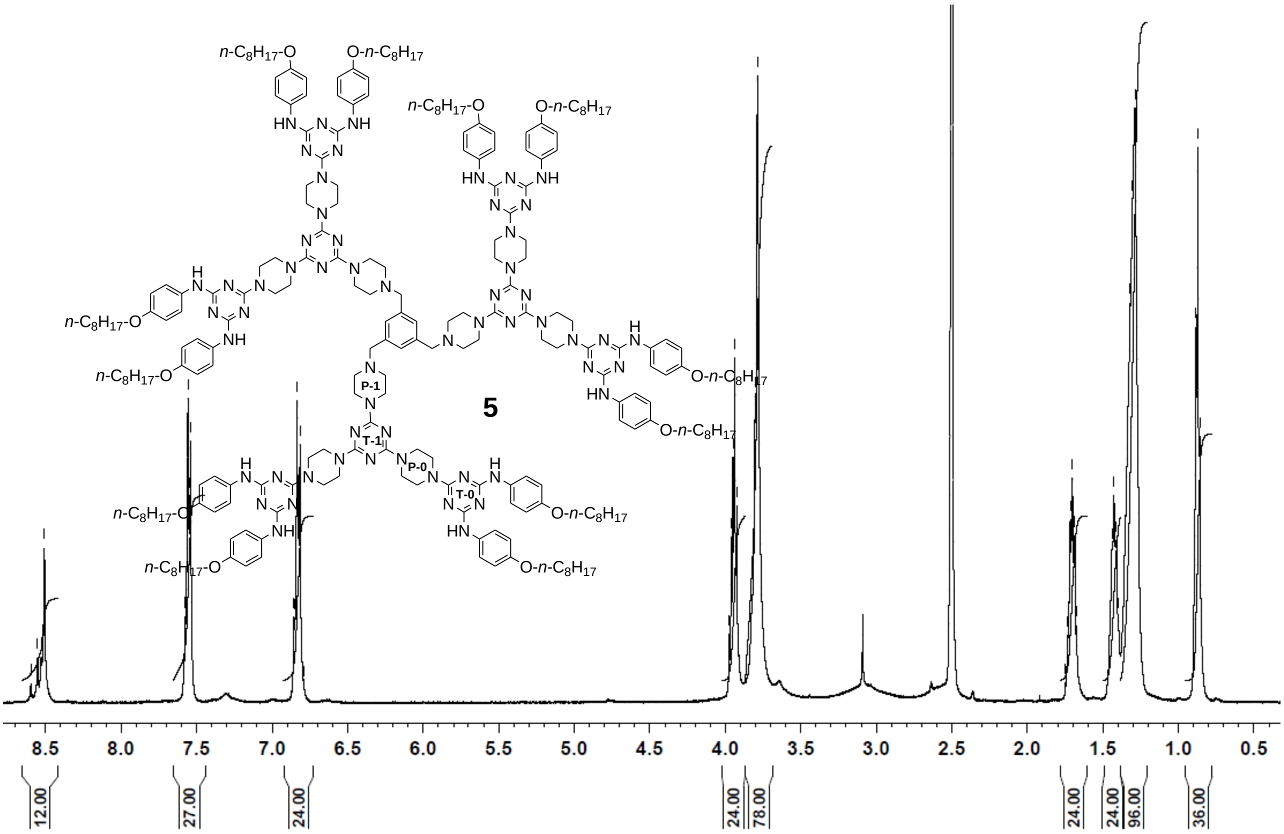

Figure S31: ^{1}H NMR spectrum of compound **5** (500 MHz, 5.0 mM in DMSO-d_6, 363 K)

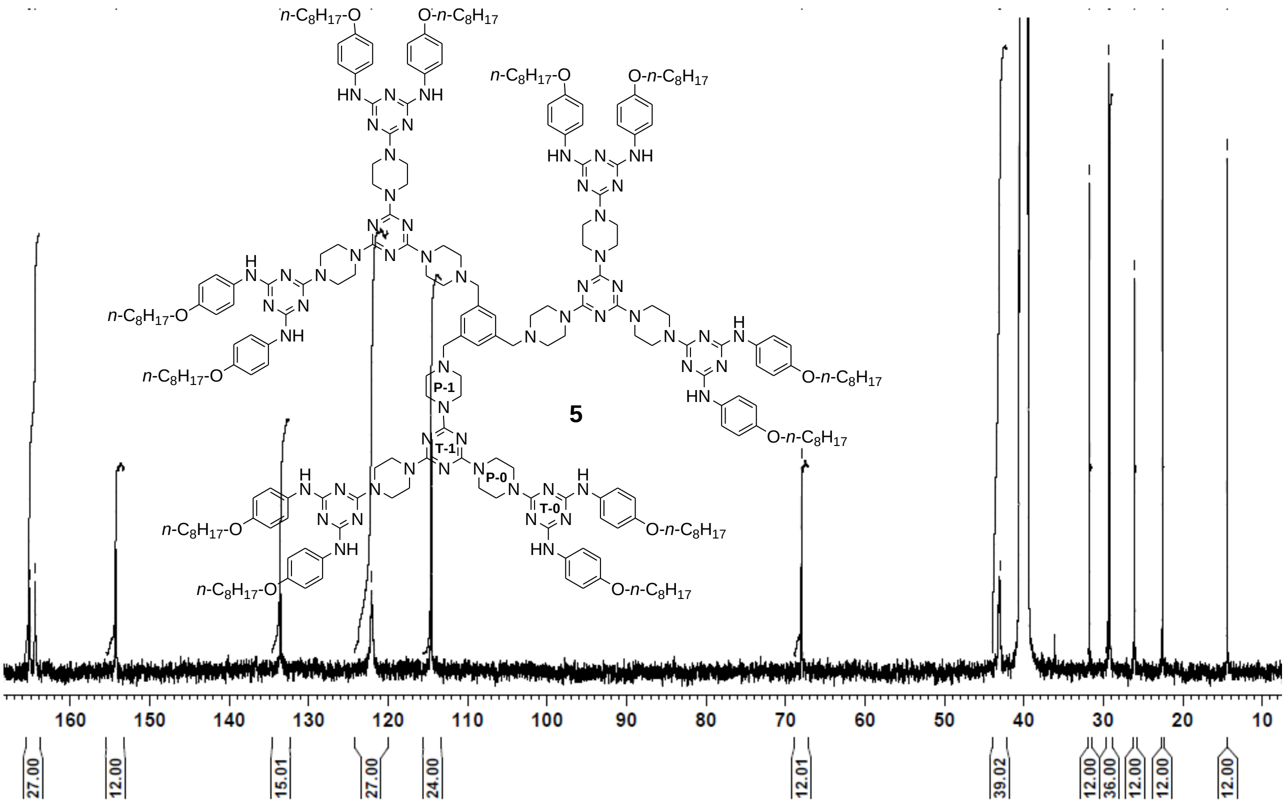

Figure S32: ^{13}QC NMR of compound **5** (125 MHz, 5.0 mM in DMSO-d_6, 298 K).

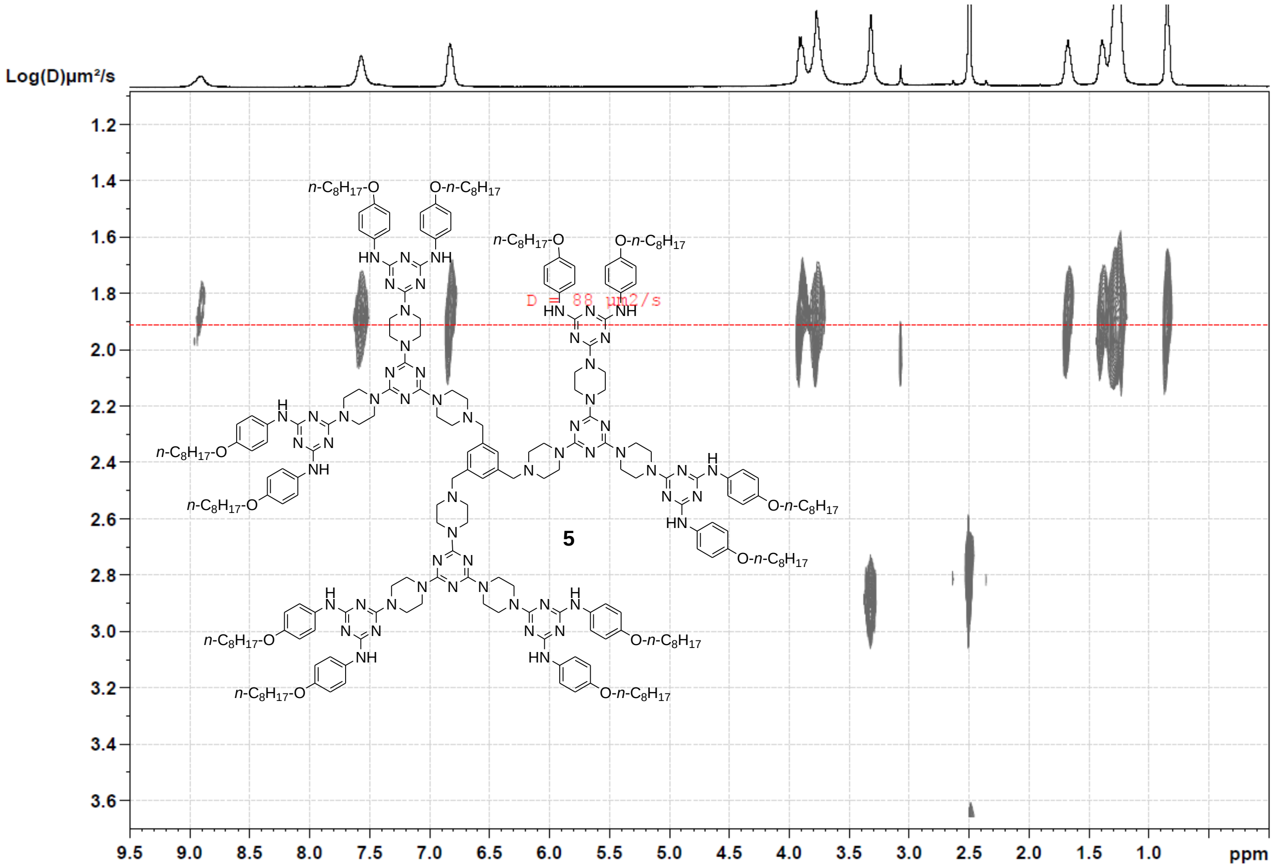

Figure S33: 2D-^{1}H-DOSY NMR chart of compound **5** (500 MHz, 5.0 mM in DMSO-d_6, 298 K).

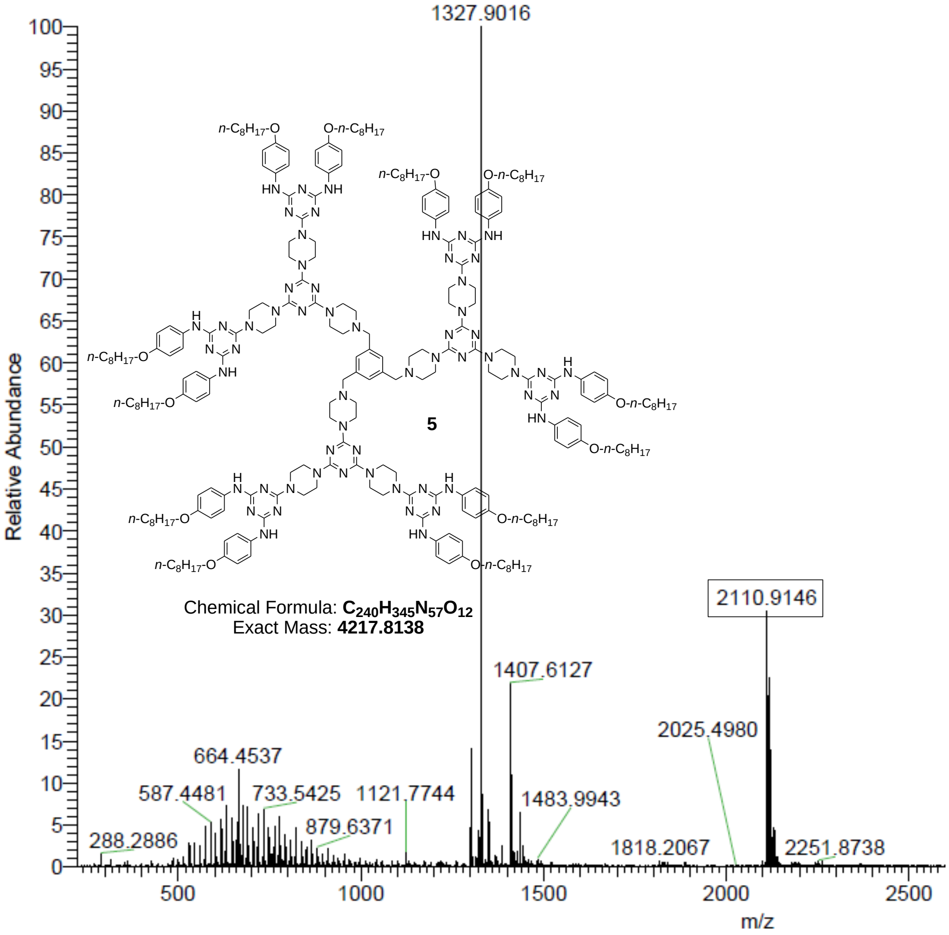

Figure S34: Mass spectrum of compound **5** [HRMS (ESI+), ACN+TFA].

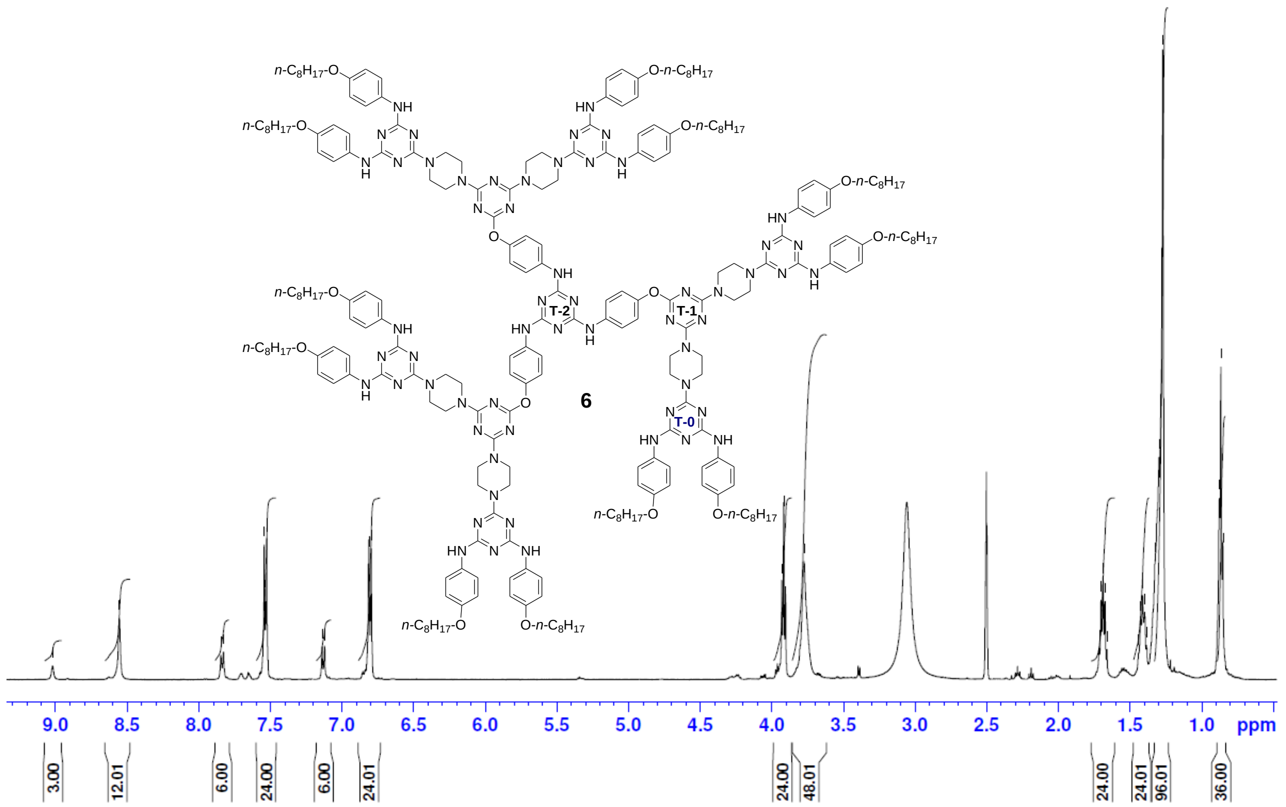

Figure S35: ^{1}H NMR spectrum of compound **6** (500 MHz, 2.5 mM in DMSO-d_6, 363 K).

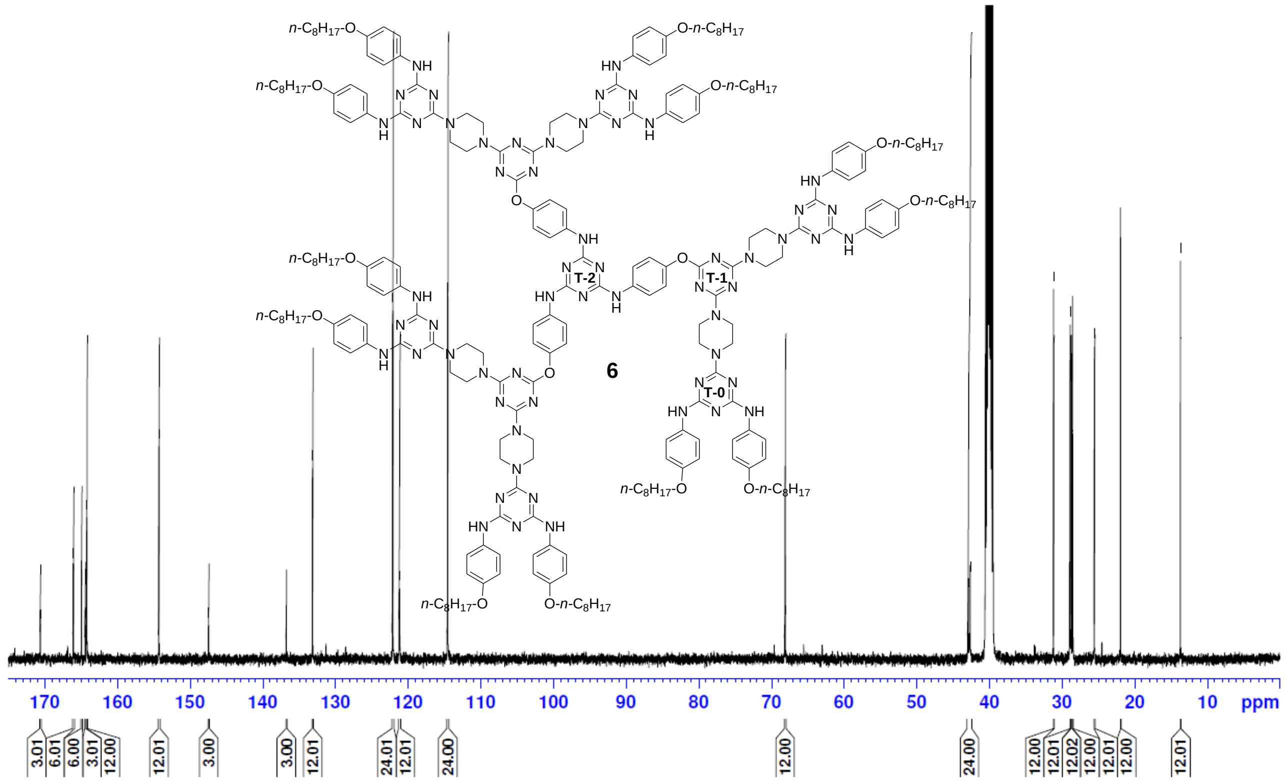

Figure S36: ^{13}QC NMR of compound **6** (125 MHz, 2.5 mM in DMSO-d_6, 363 K).

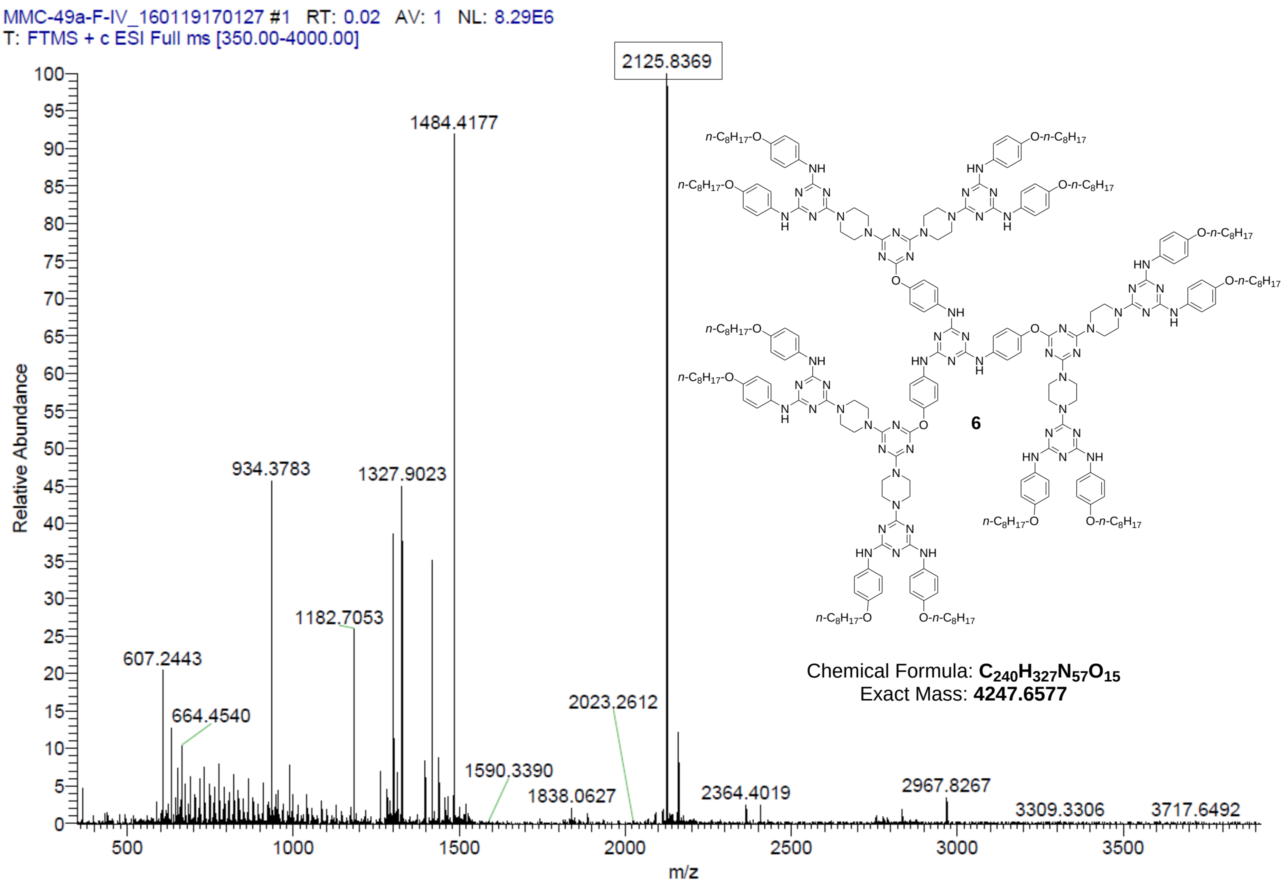

Figure S37: Mass spectrum of compound **6** [HRMS (ESI+), ACN+TFA].

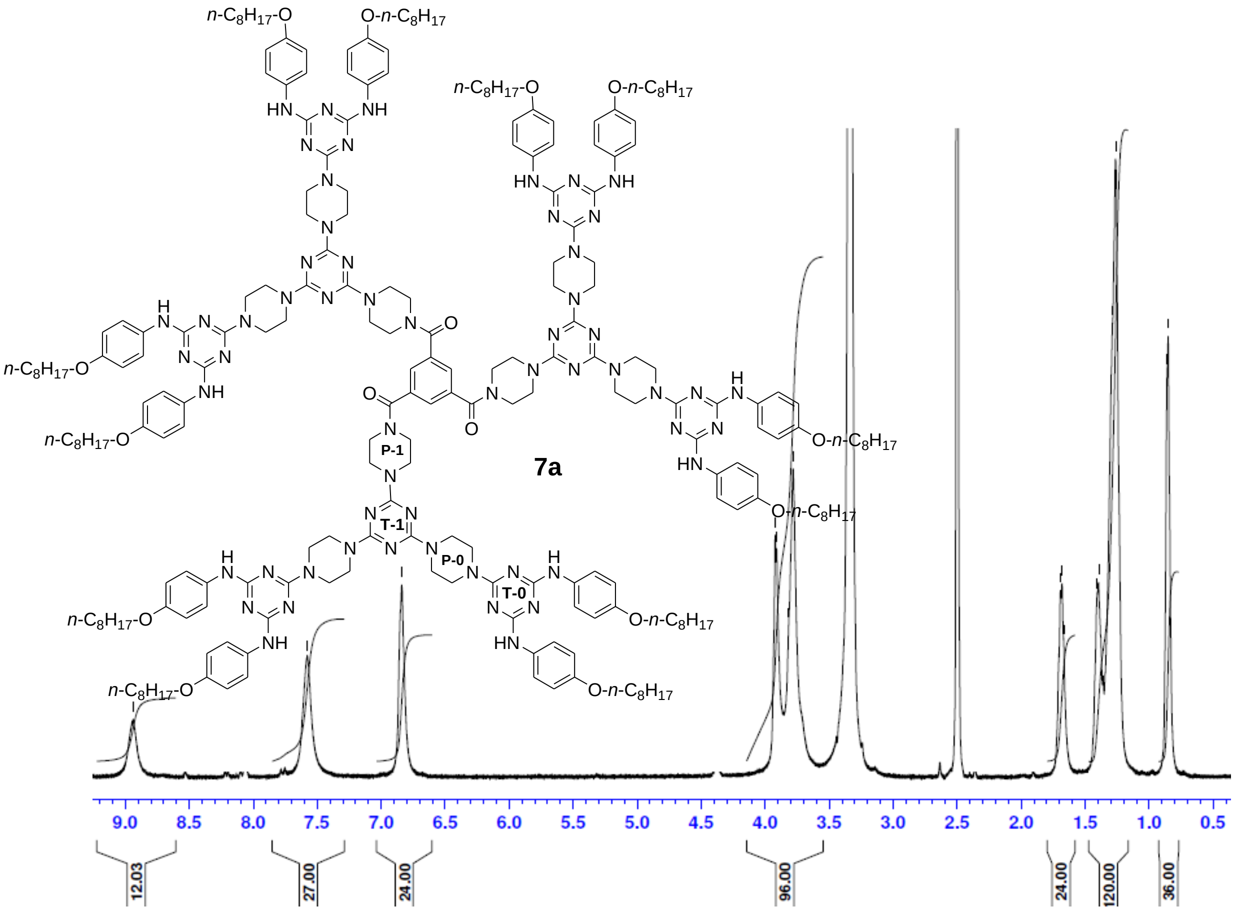

Figure S38: ^{1}H NMR apectrum of compound **7a** (500 MHz, 2.5 mM in DMSO-d_6, 298 K).

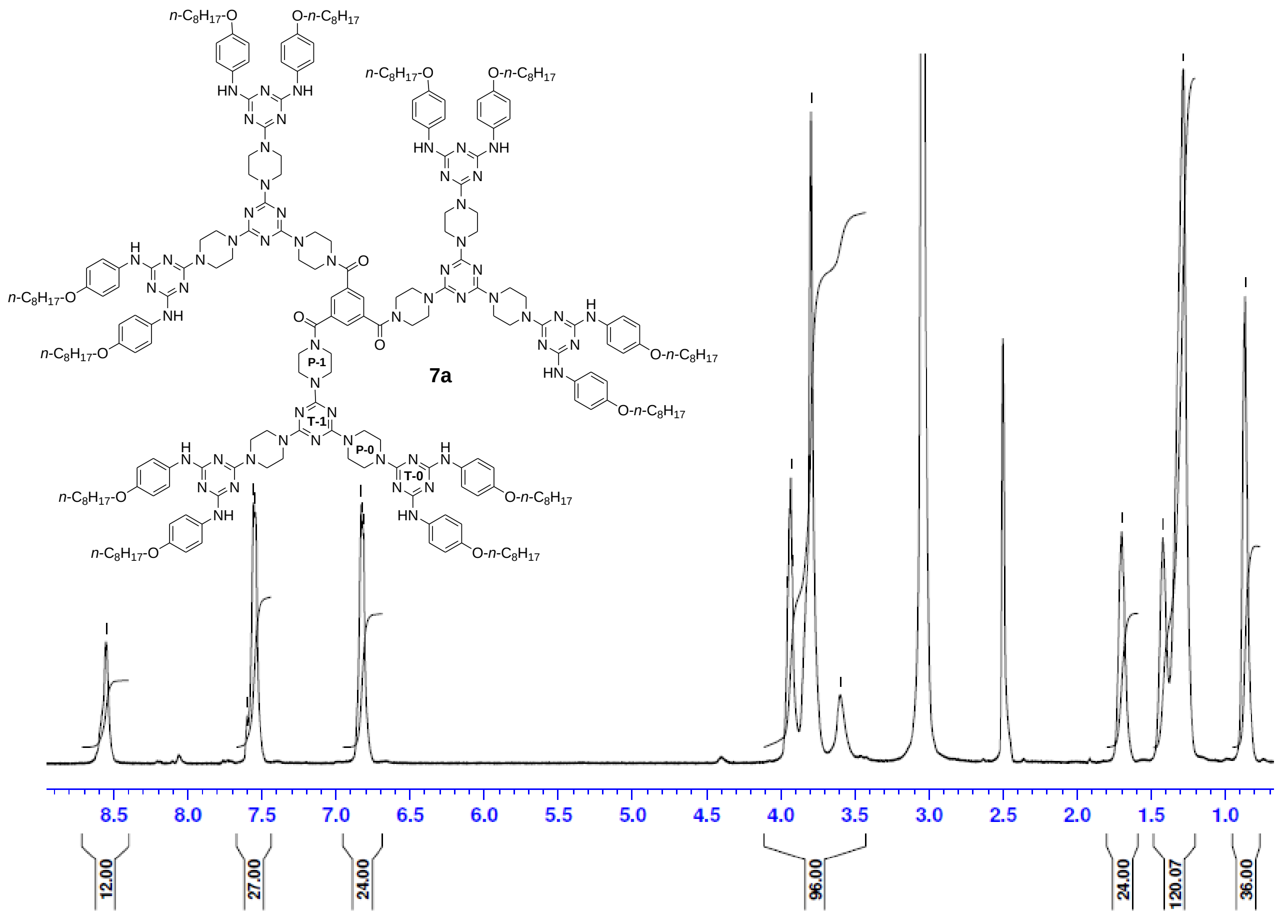

Figure S39: ^{1}H NMR spectrum of compound **7a** (500 MHz, 2.5 mM in DMSO-d_6, 363 K).

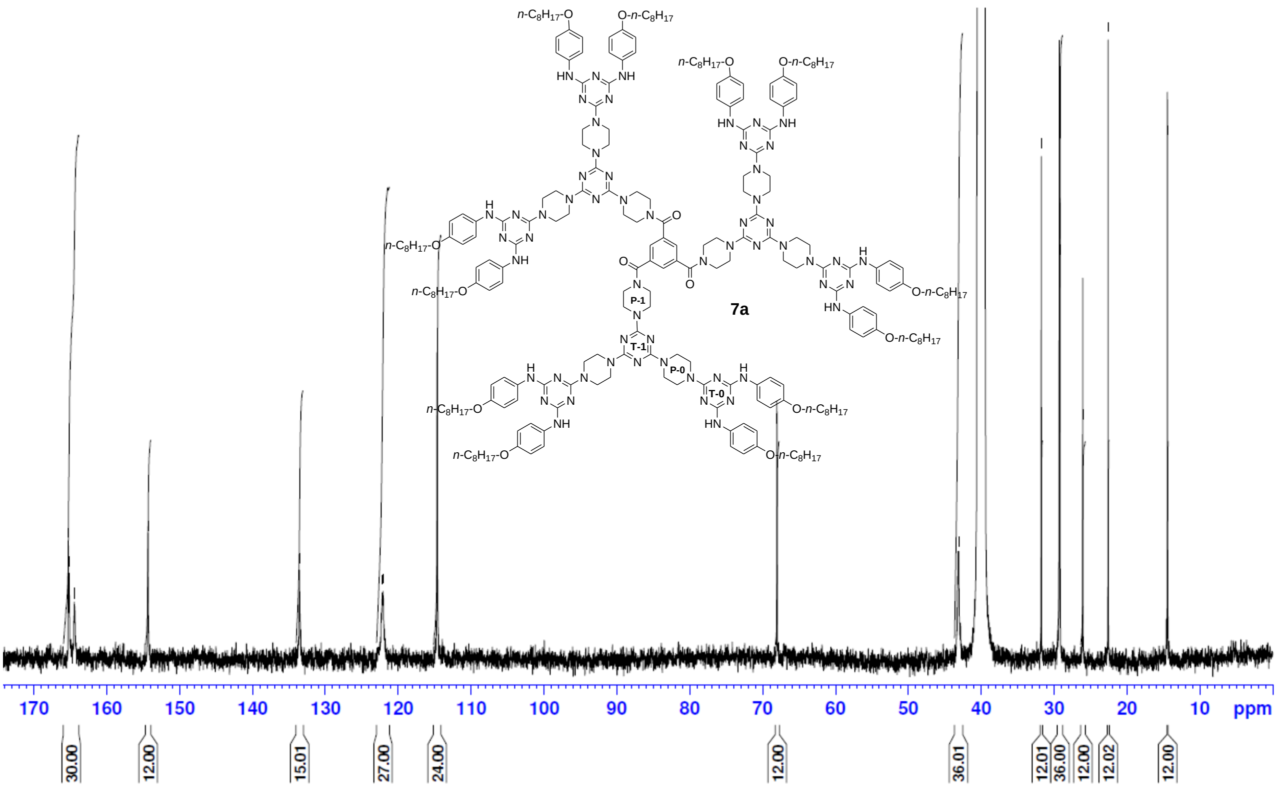

Figure S40: ^{13}QC NMR spectrum of compound **7a** (125 MHz, 5.0 mM in DMSO-d_6, 298 K).

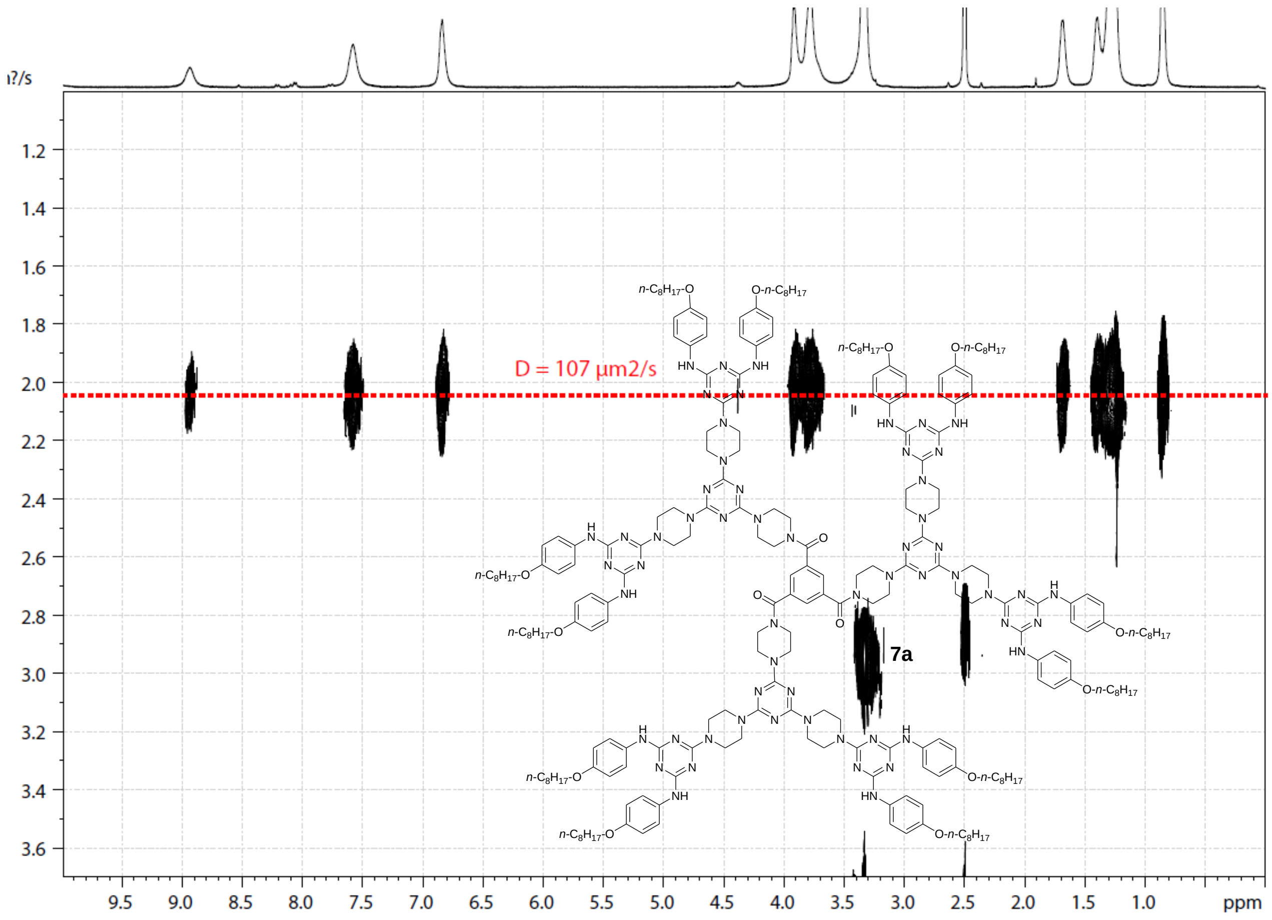

Figure S41: 2D-^{1}H-DOSY NMR chart of compound **7a** (500 MHz, 2.5 mM in DMSO-d_6, 298 K).

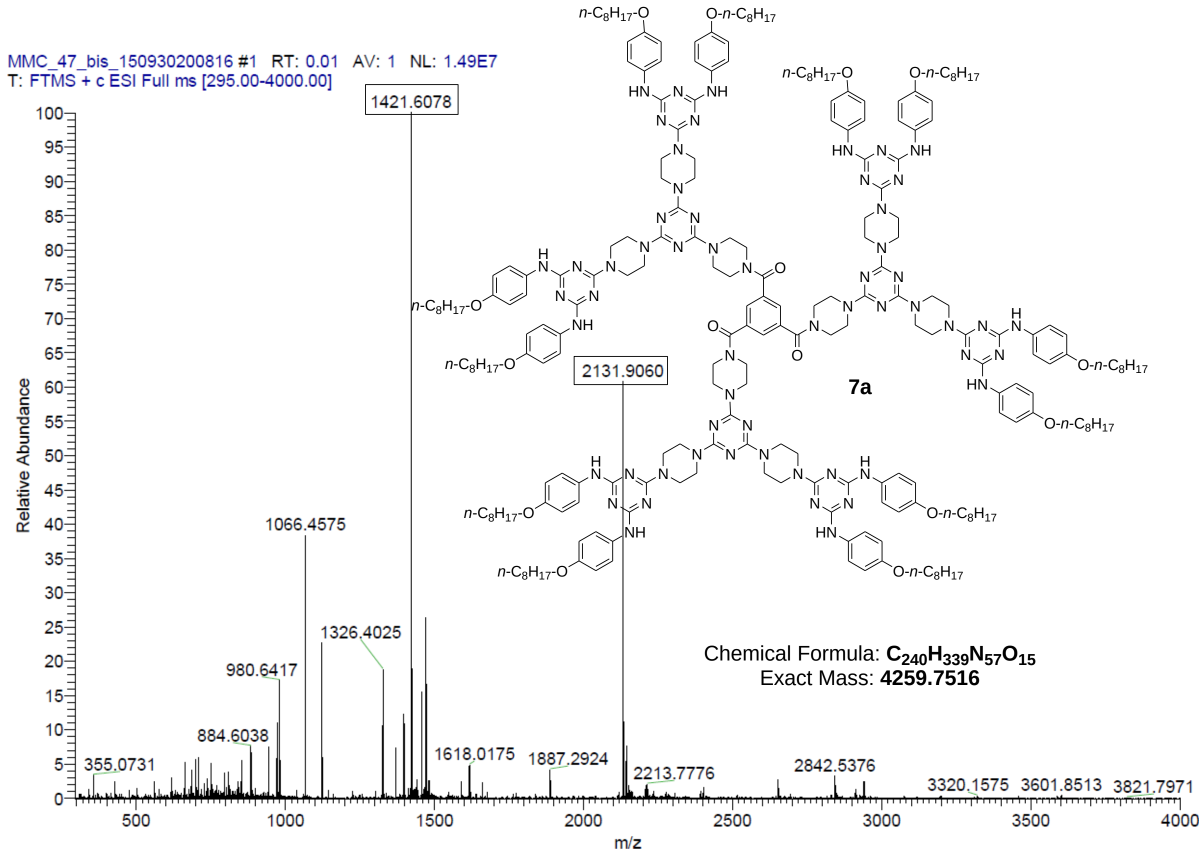

Figure S42: Mass spectrum of compound **7a** [HRMS (ESI+), ACN+TFA].

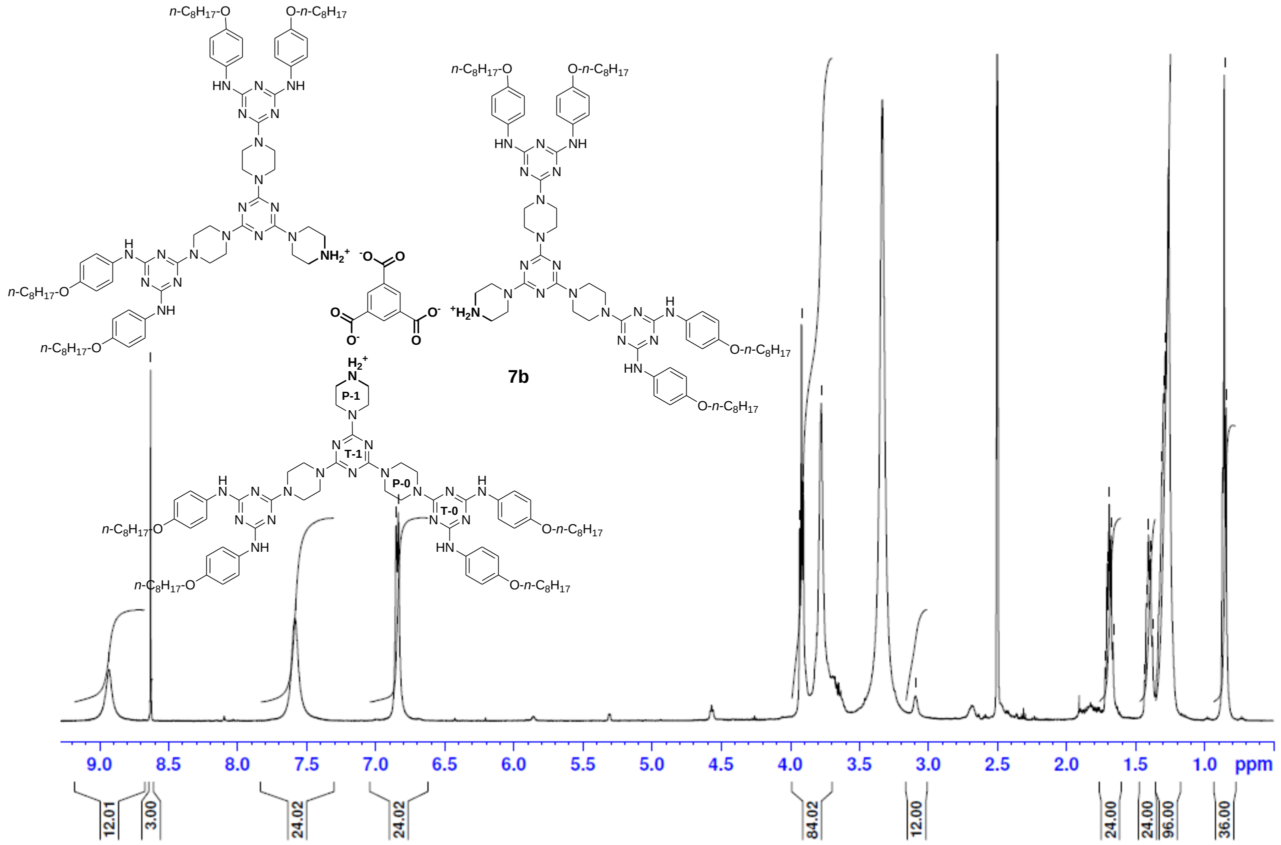

Figure S43: ^{1}H NMR spectrum of compound 7**b** (500 MHz, 2.5 mM in DMSO-d_6, 298 K).

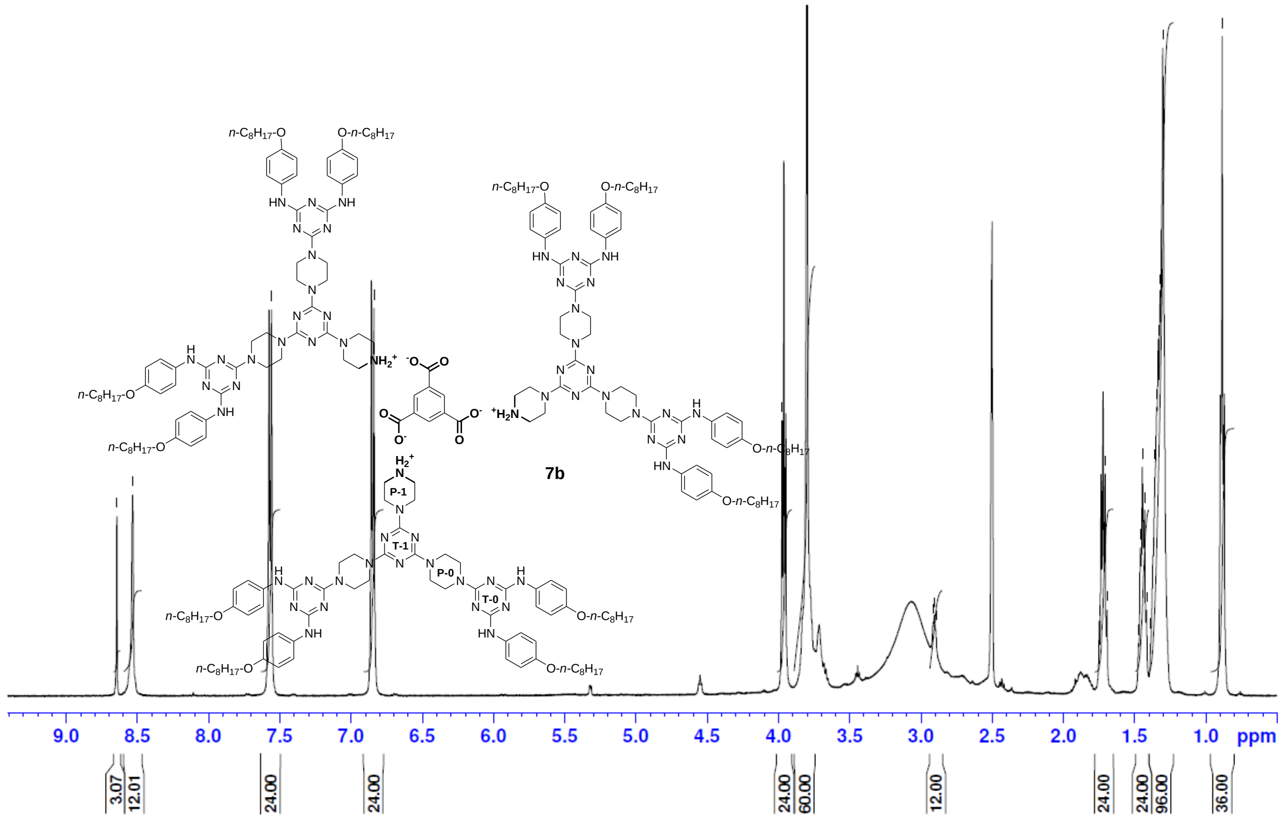

Figure S44: ^{1}H NMR spectrum of compound 7**b** (500 MHz, 2.5 mM in DMSO-d_6, 363 K).

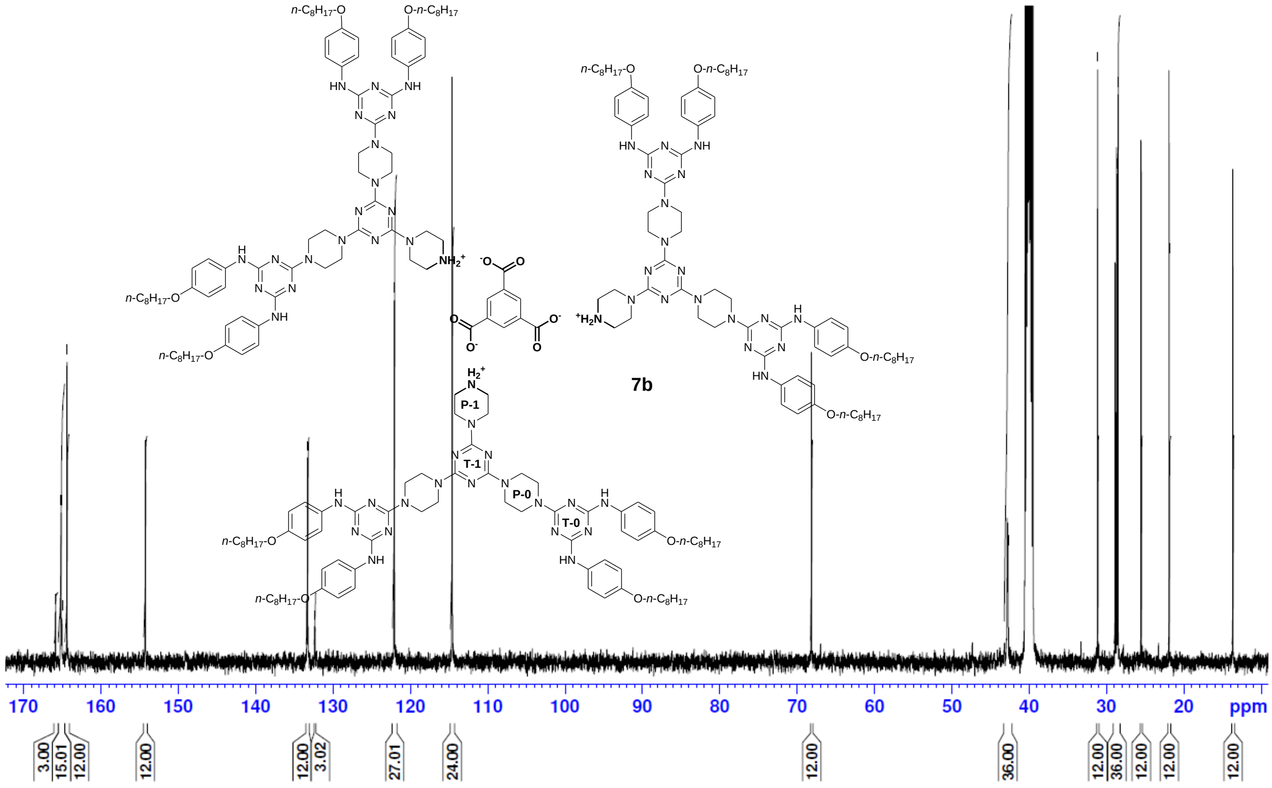

Figure S45: ^{13}QC NMR spectrum of compound **7b** (125 MHz, 2.5 mM in DMSO-d_6, 298 K).

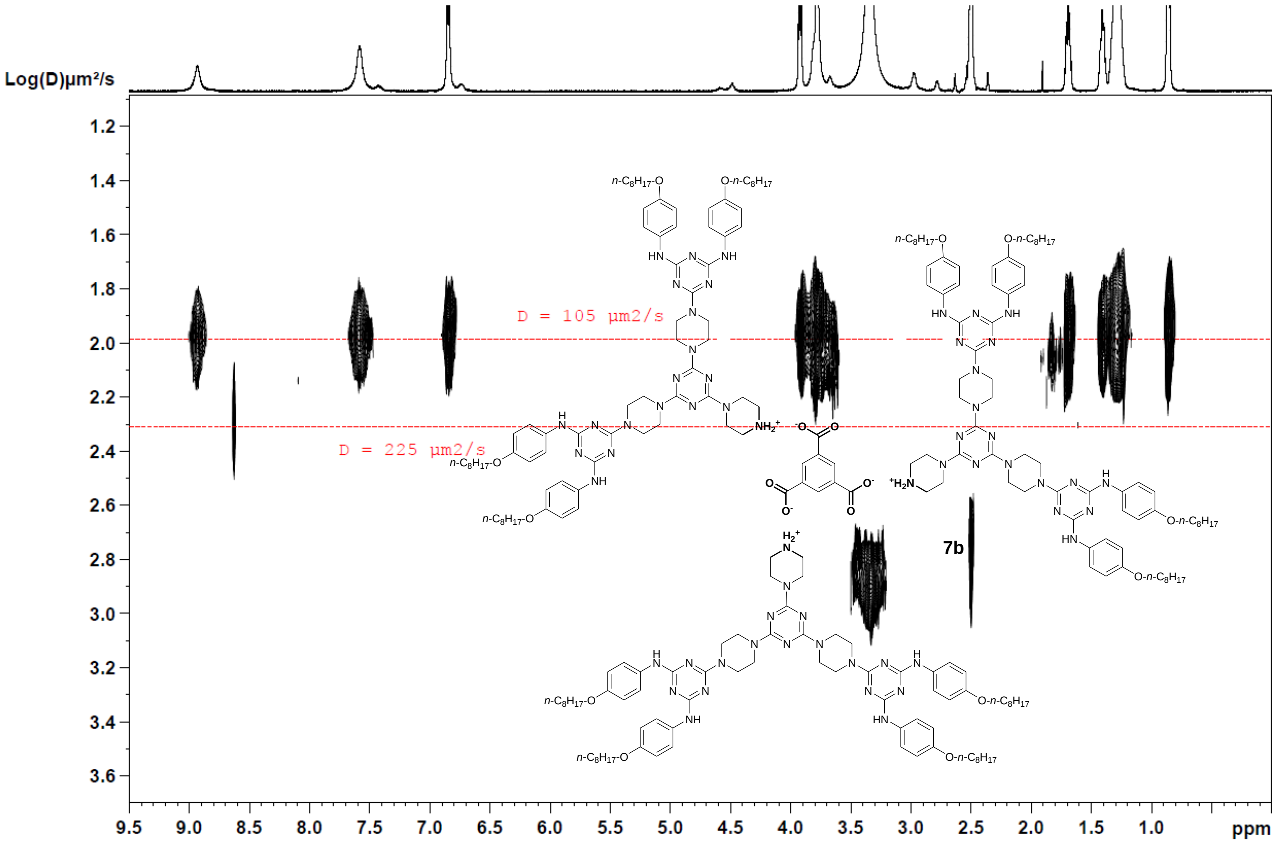

Figure S46: 2D-^{1}H-DOSY NMR chart of compound 7**b** (500 MHz, 2.5 mM in DMSO-d_6, 298 K).

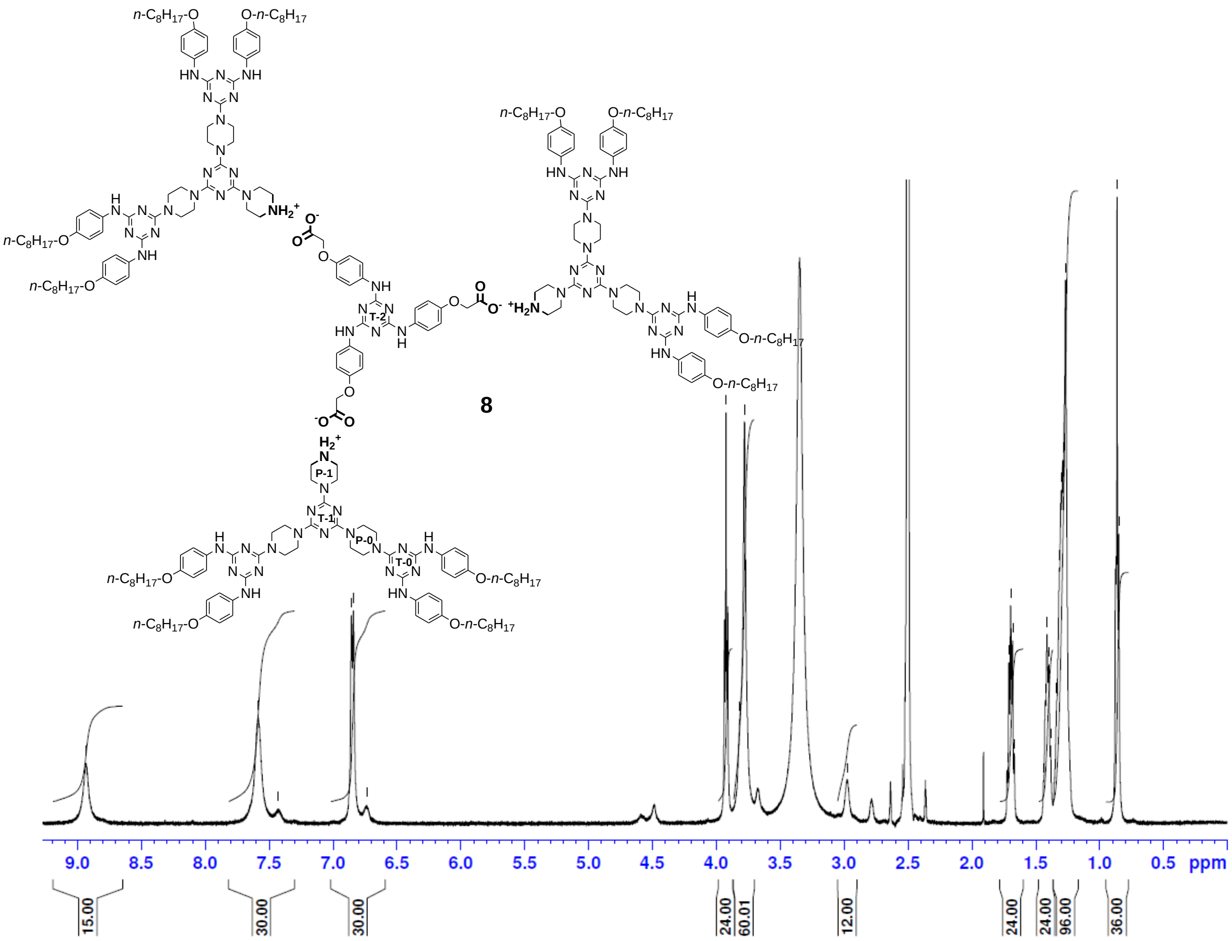

Figure S47: ^{1}H NMR spectrum of compound **8** (500 MHz, 5.0 mM in DMSO-d_6, 298 K).

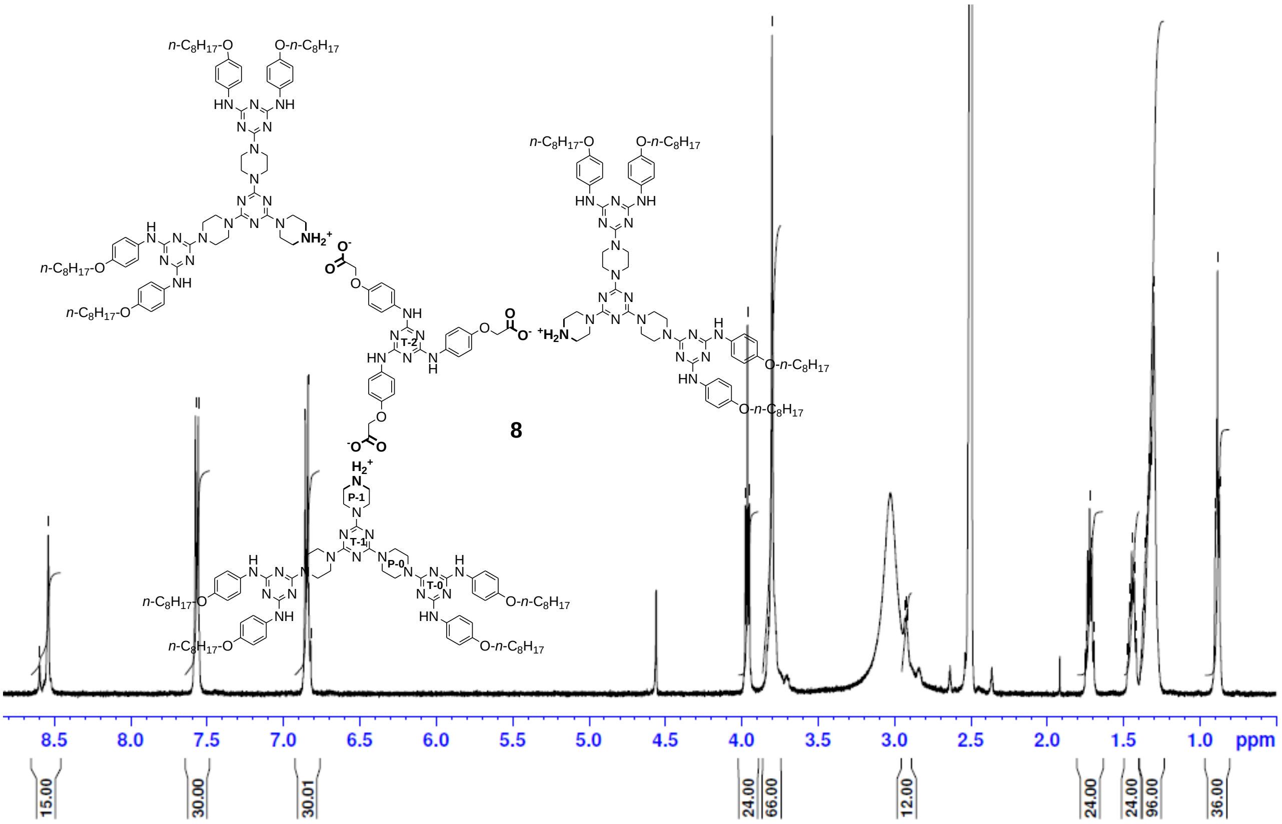

Figure S48: ^{1}H NMR spectrum of compound **8** (500 MHz, 5.0 mM in DMSO-d_6, 363 K).

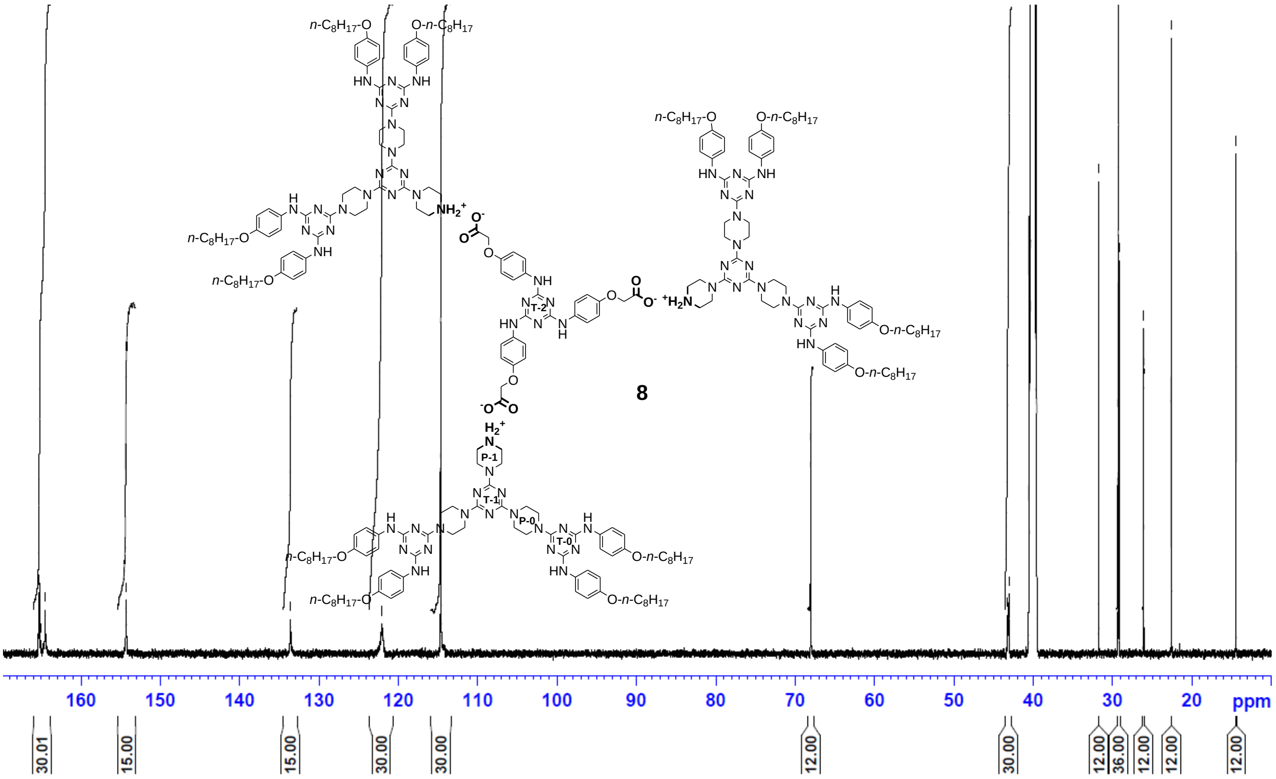

Figure S49: ^{13}QC NMR spectrum of compound **8** (125 MHz, 5.0 mM in DMSO-d_6, 298 K).

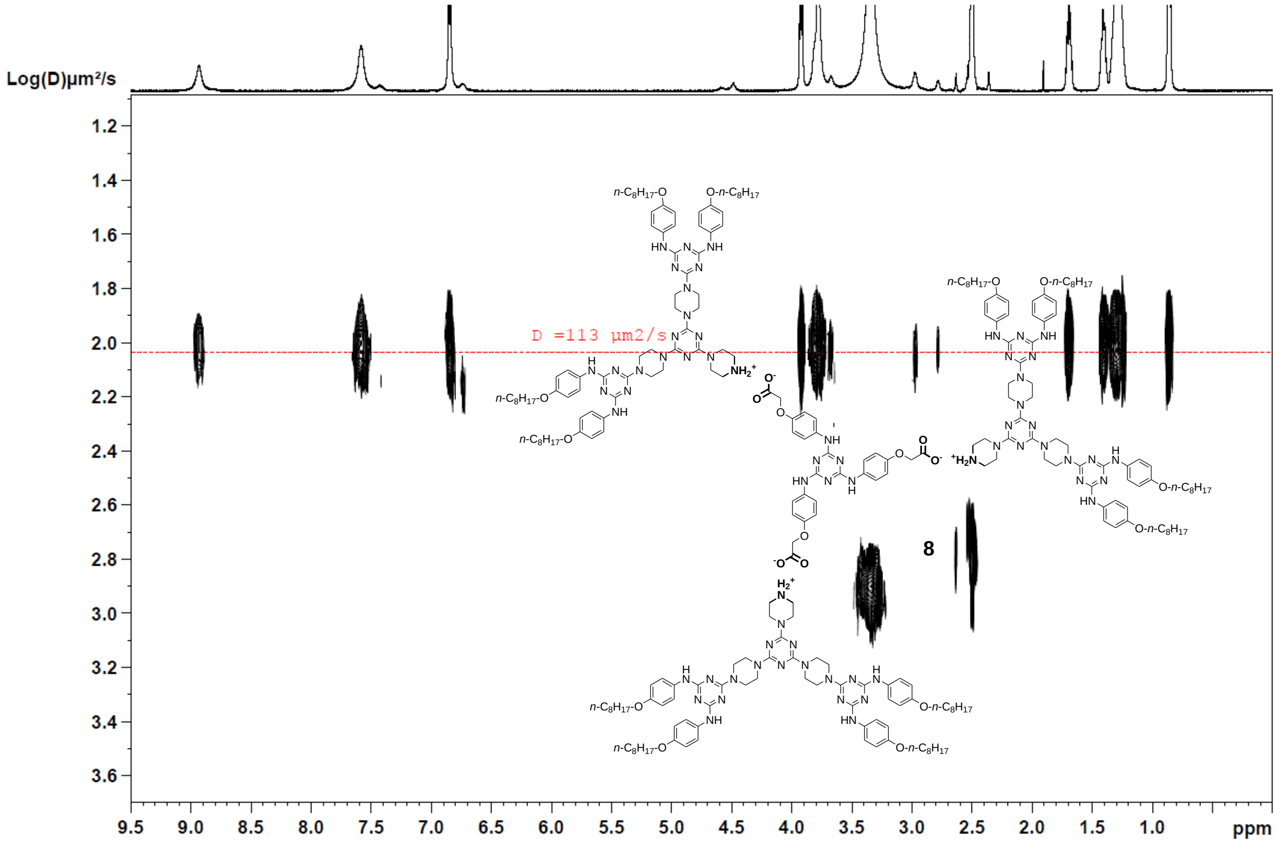

Figure S50: 2D-^{1}H-DOSY NMR chart of compound **8** (500 MHz, 5.0 mM in DMSO-d_6, 298 K).

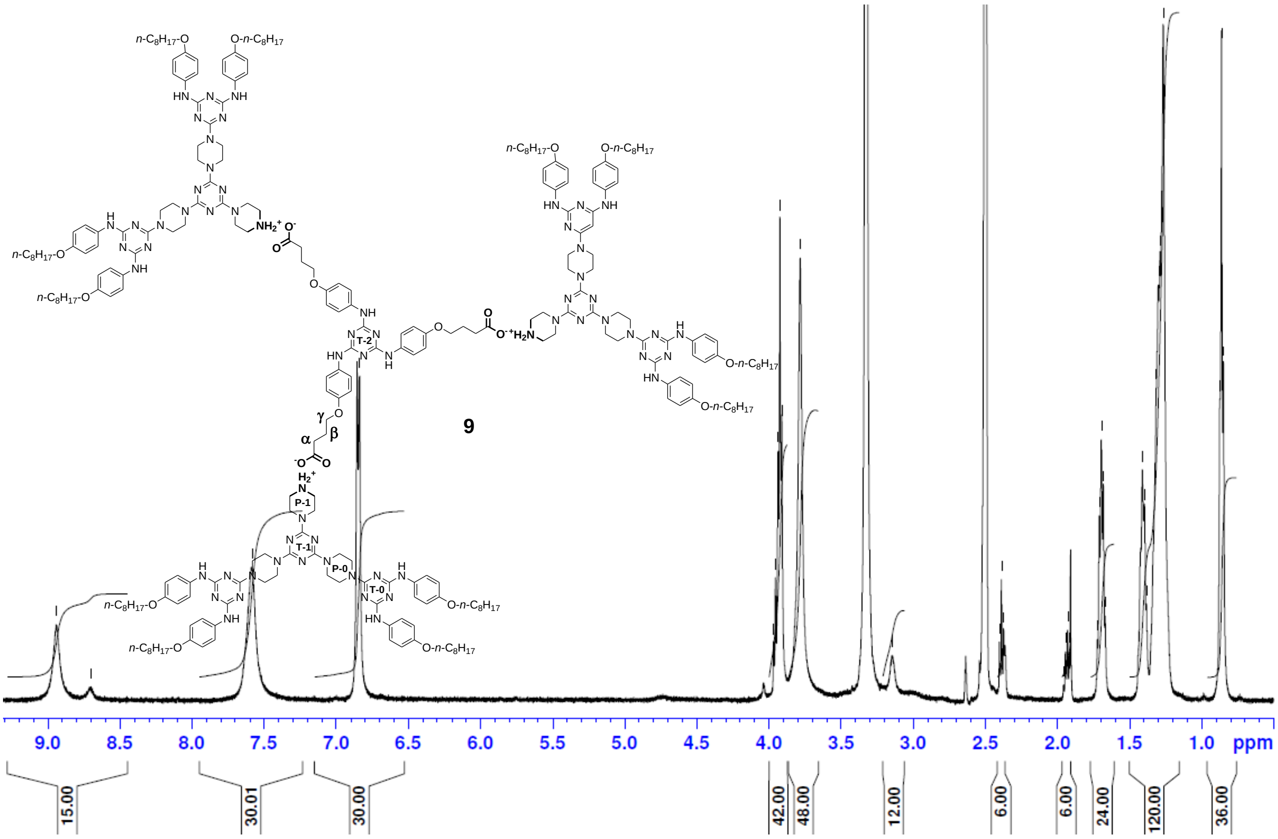

Figure S51: ^{1}H NMR spectrum of compound **9** (500 MHz, 5.0 mM in DMSO-d_6, 298 K).

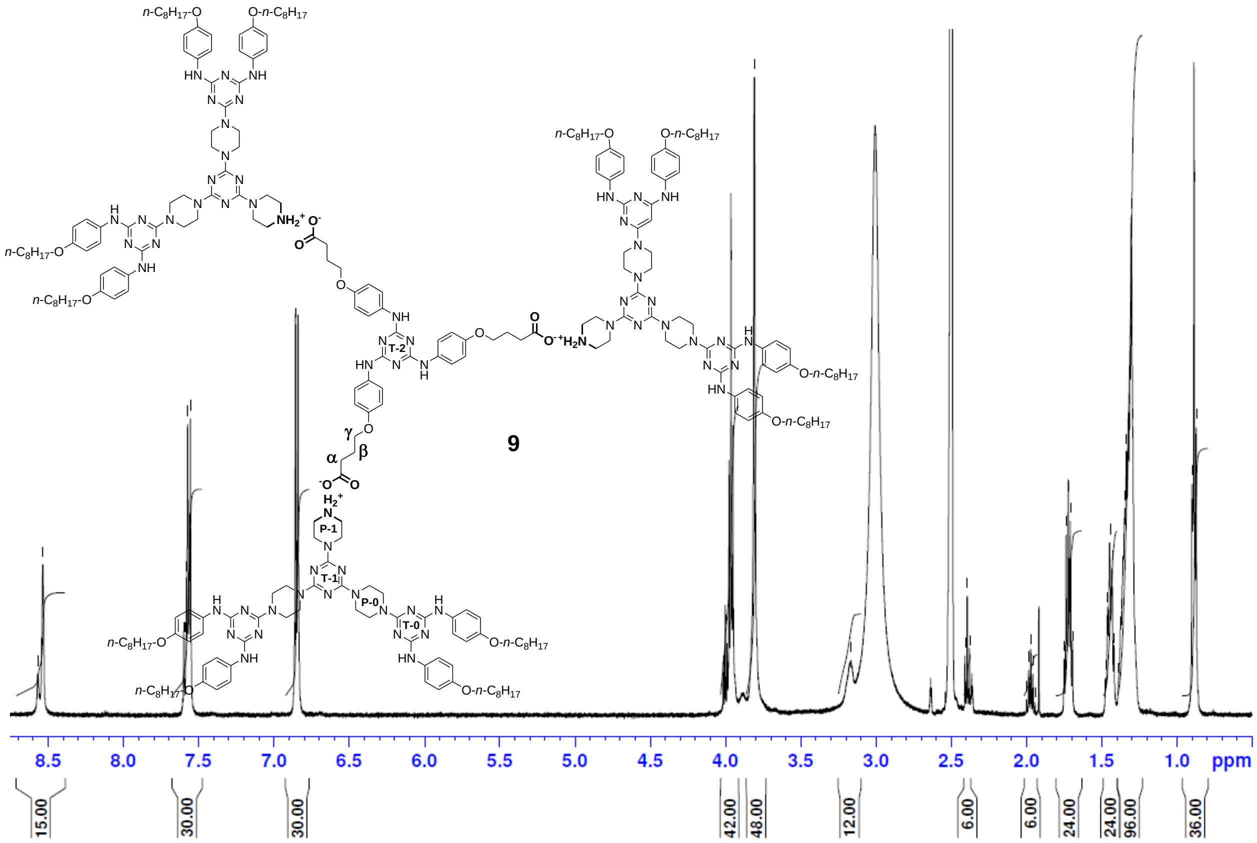

Figure S52: ^{1}H NMR spectrum of compound **9** (500 MHz, 5.0 mM in DMSO-d_6, 363 K).

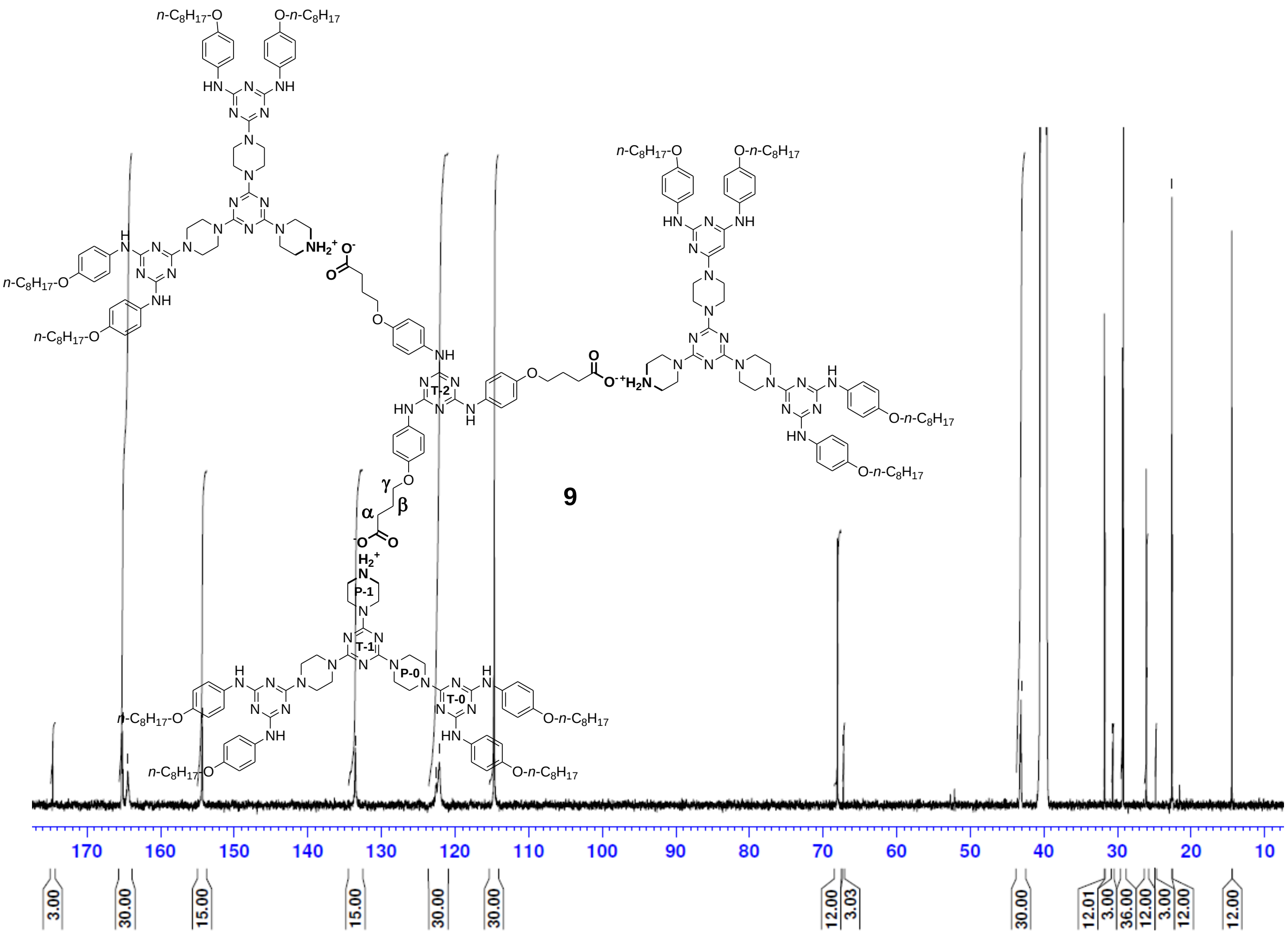

Figure S53: ^{13}QC NMR spectrum of compound **9** (125 MHz, 5.0 mM in DMSO-d_6, 298 K).

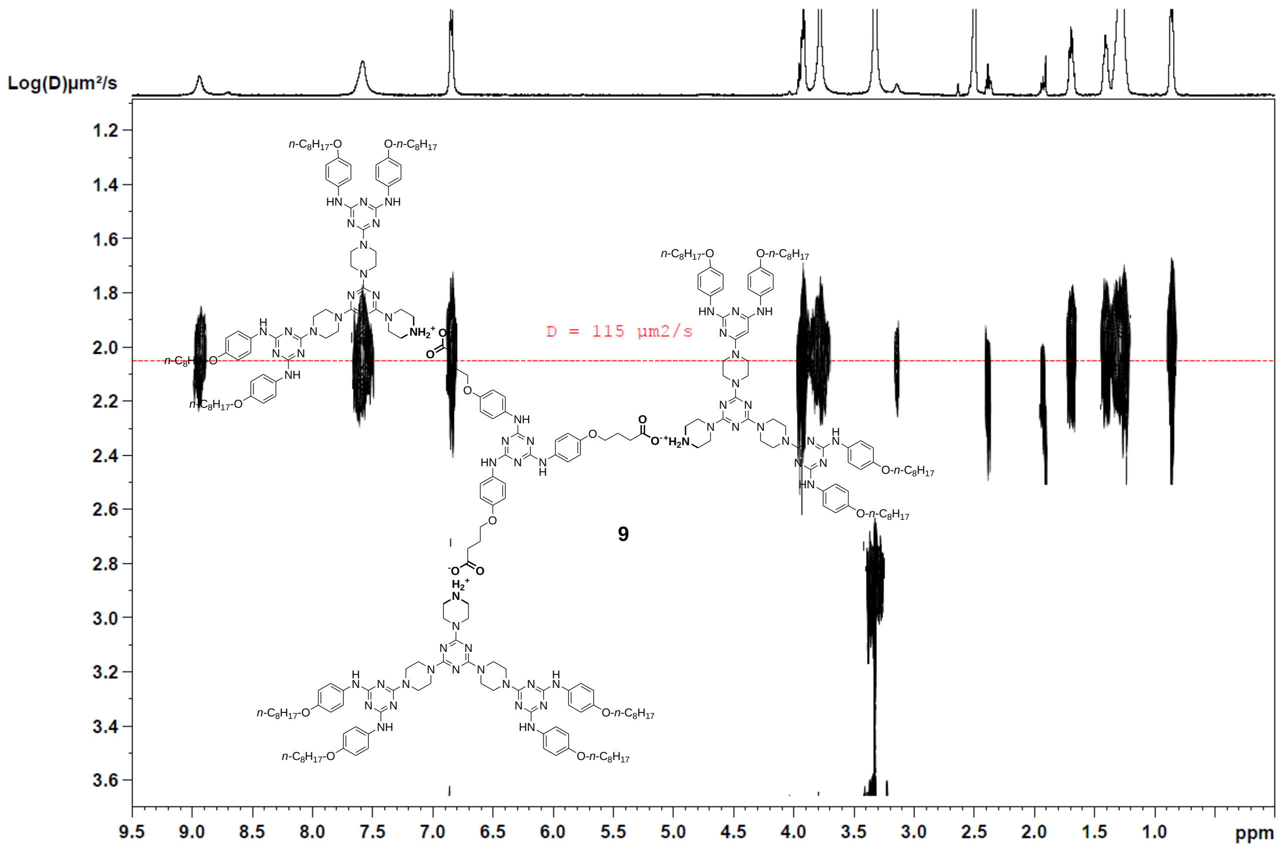

Figure S54: 2D-^{1}H-DOSY NMR chart of compound **9** (500 MHz, 5.0 mM in DMSO-d_6, 298 K).

Extracted data from the output files of the ΔG of solvation calculation for compounds **2a, 3**, **D-Cl** and **D-N<P>NH**

1. Compound **2a** inTHF

(Unpolarised solute)-Solvent (Kcal mol^{-1}) = −15.41
(Polarised solute)-Solvent (Kcal mol^{-1}) = −19.73
Solute polarisation (Kcal mol^{-1}) = +2.36
Total electrostatic (Kcal mol^{-1}) = −17.38

SMD-CDS (non-electrostatic) energy (Kcal mol^{-1}) = −17.60
Total non electrostatic (Kcal mol^{-1}) = −17.60
Δ*G* of solvation (Kcal mol^{-1}) = −34.97
----

2. Compound **2a** in 1,4 Dioxane

(Unpolarised solute)-Solvent (Kcal mol^{-1}) = −9.43
(Polarised solute)-Solvent (Kcal mol^{-1}) = −10.95
Solute polarisation (Kcal mol^{-1}) = +0.80
Total electrostatic (Kcal mol^{-1}) = −10.15

SMD-CDS (non-electrostatic) energy (Kcal mol^{-1}) = −14.34
Total non electrostatic (Kcal mol^{-1}) = −14.34
Δ*G* of solvation (Kcal mol^{-1}) = −24.49
----

3. Compound **3** in THF

(Unpolarised solute)-Solvent (Kcal mol^{-1}) = −17.48
(Polarised solute)-Solvent (Kcal mol^{-1}) = −22.72
Solute polarisation (Kcal mol^{-1}) = +2.89
Total electrostatic (Kcal mol^{-1}) = −19.83

SMD-CDS (non-electrostatic) energy (Kcal mol^{-1}) = −20.21
Total non electrostatic (Kcal mol^{-1}) = −20.21
Δ*G* of solvation (Kcal mol^{-1}) = −40.03
----

4. Compound **3** in 1,4 Dioxane

(Unpolarised solute)-Solvent (Kcal mol^{-1}) = −10.78
(Polarised solute)-Solvent (Kcal mol^{-1}) = −12.55
Solute polarisation (Kcal mol^{-1}) = +0.94
Total electrostatic (Kcal mol^{-1}) = −11.61

SMD-CDS (non-electrostatic) energy (Kcal mol^{-1}) = −17.66
Total non electrostatic (Kcal mol^{-1}) = −17.66
Δ*G* of solvation (Kcal mol^{-1}) = −29.27
----

5. Compound **D-Cl** in THF

(Unpolarised solute)-Solvent (Kcal mol^{-1}) = −36.81
(Polarised solute)-Solvent (Kcal mol^{-1}) = −48.00
Solute polarisation (Kcal mol^{-1}) = +6.19
Total electrostatic (Kcal mol^{-1}) = −41.82

SMD-CDS (non-electrostatic) energy (Kcal mol^{-1}) = −48.66
Total non electrostatic (Kcal mol^{-1}) = −48.66
Δ*G* of solvation (Kcal mol^{-1}) = −90.47

----

6. Compound **D-Cl** in 1,4-Dioxane

(Unpolarised solute)-Solvent (Kcal mol^{-1}) = −22.56
(Polarised solute)-Solvent (Kcal mol^{-1}) = −26.32
Solute polarisation (Kcal mol^{-1}) = +1.99
Total electrostatic (Kcal mol^{-1}) = −24.33

SMD-CDS (non-electrostatic) energy (Kcal mol^{-1}) = −43.60
Total non electrostatic (Kcal mol^{-1}) = −43.60
Δ*G* of solvation (Kcal mol^{-1}) = −67.92

----

7. Compound **D-N<P>NH** in THF

(Unpolarised solute)-Solvent (Kcal mol^{-1}) = −38.87
(Polarised solute)-Solvent (Kcal mol^{-1}) = −50.43
Solute polarisation (Kcal mol^{-1}) = +6.37
Total electrostatic (Kcal mol^{-1}) = −44.07

SMD-CDS (non-electrostatic) energy (Kcal mol^{-1}) = −51.93
Total non electrostatic (Kcal mol^{-1}) = −51.93
Δ*G* of solvation (Kcal mol^{-1}) = −96.00

----

8. Compound **D-N<P>NH** in 1,4-Dioxane

(Unpolarised solute)-Solvent (Kcal mol^{-1}) = −23.84
(Polarised solute)-Solvent (Kcal mol^{-1}) = −27.73
Solute polarisation (Kcal mol^{-1}) = +2.06
Total electrostatic (Kcal mol^{-1}) = −25.67

SMD-CDS (non-electrostatic) energy (Kcal mol^{-1}) = −46.33
Total non electrostatic (Kcal mol^{-1}) = −46.33
Δ*G* of solvation (Kcal mol^{-1}) = −72.00

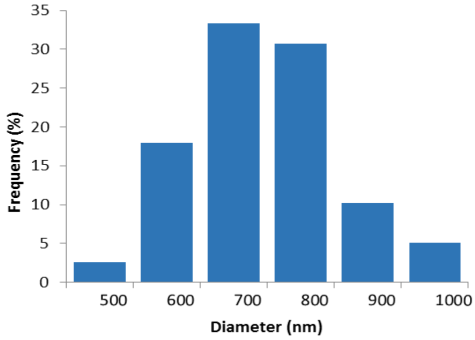

D-N<P>NH

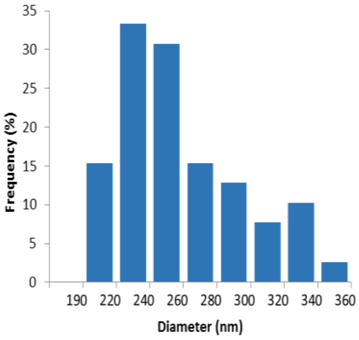

5

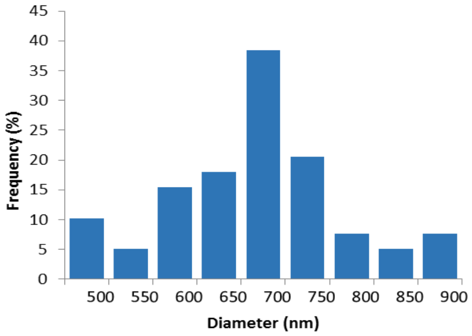

6

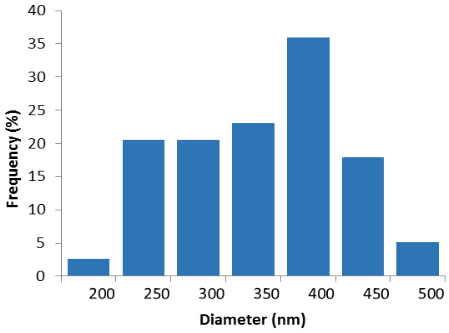

7a

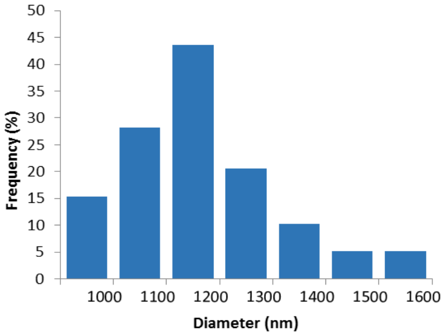

7b

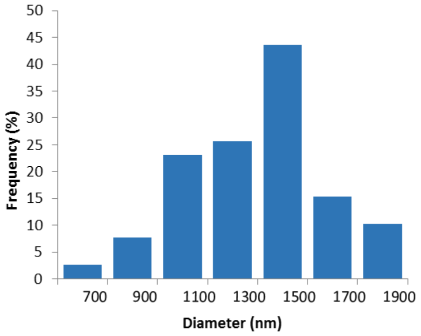

8

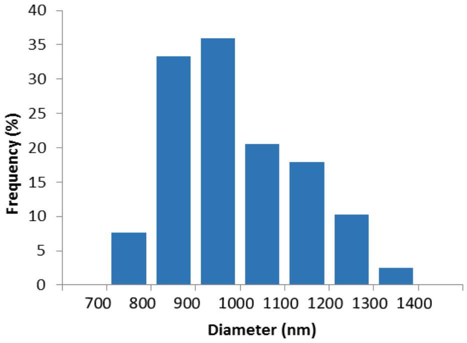

9

Figure S55: Spherical nano-aggregates diameter distributions of compounds **D-N<P>NH** and **5-9** (from DMSO).

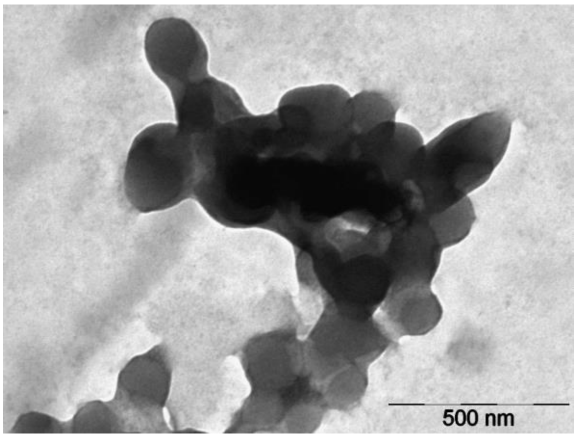

Figure S56: TEM image of compound **4** (from DMSO)
